# Cancer-Selective Targeting of the NF-κB Survival Pathway with GADD45β/MKK7 Inhibitors

**DOI:** 10.1016/j.ccr.2014.07.027

**Published:** 2014-10-13

**Authors:** Laura Tornatore, Annamaria Sandomenico, Domenico Raimondo, Caroline Low, Alberto Rocci, Cathy Tralau-Stewart, Daria Capece, Daniel D’Andrea, Marco Bua, Eileen Boyle, Mark van Duin, Pietro Zoppoli, Albert Jaxa-Chamiec, Anil K. Thotakura, Julian Dyson, Brian A. Walker, Antonio Leonardi, Angela Chambery, Christoph Driessen, Pieter Sonneveld, Gareth Morgan, Antonio Palumbo, Anna Tramontano, Amin Rahemtulla, Menotti Ruvo, Guido Franzoso

**Affiliations:** 1 Department of Medicine, Centre for Cell Signalling and Inflammation, Imperial College London, London W12 0NN, UK; 2 Institute of Biostructures and Bioimages, National Research Council and CIRPeB, 80134 Naples, Italy; 3 Department of Physics, “Sapienza” University, 00185 Rome, Italy; 4 Drug Discovery Centre, Imperial College London, London W6 8RP, UK; 5 Division of Hematology, University of Torino, AOU San Giovanni Battista, 10126 Turin, Italy; 6 Department of Medicine, Centre for Haematology, Imperial College London, London W12 0NN, UK; 7 Section of Haemato-Oncology, The Institute of Cancer Research, London SM2 5NG, UK; 8 Department of Hematology, Erasmus University Medical Center, 3000 CA Rotterdam, the Netherlands; 9 Institute for Cancer Genetics, Columbia University Medical Center, New York, NY 10032, USA; 10 Department of Medicine, Section of Molecular Immunology, Imperial College London, London W12 0NN, UK; 11 Department of Molecular Medicine and Medical Biotechnologies, University of Naples “Federico II,” 80131 Naples, Italy; 12 Department of Environmental, Biological, and Pharmaceutical Sciences and Technologies, Second University of Naples, 81100 Caserta, Italy; 13 IRCCS Multimedica, 20138 Milan, Italy; 14 Department of Oncology/Hematology, Kantonsspital St. Gallen, 9007 St. Gallen, Switzerland; 15 Istituto Pasteur Fondazione Cenci Bolognetti, “Sapienza” University, 00185 Rome, Italy

## Abstract

Constitutive NF-κB signaling promotes survival in multiple myeloma (MM) and other cancers; however, current NF-κB-targeting strategies lack cancer cell specificity. Here, we identify the interaction between the NF-κB-regulated antiapoptotic factor GADD45β and the JNK kinase MKK7 as a therapeutic target in MM. Using a drug-discovery strategy, we developed DTP3, a D-tripeptide, which disrupts the GADD45β/MKK7 complex, kills MM cells effectively, and, importantly, lacks toxicity to normal cells. DTP3 has similar anticancer potency to the clinical standard, bortezomib, but more than 100-fold higher cancer cell specificity in vitro. Notably, DTP3 ablates myeloma xenografts in mice with no apparent side effects at the effective doses. Hence, cancer-selective targeting of the NF-κB pathway is possible and, at least for myeloma patients, promises a profound benefit.

## Highlights

• GADD45β is a critical mediator of the NF-κB antiapoptotic function in MM
• GADD45β binds to MKK7 and promotes MM cell survival by blocking MKK7/JNK signaling
• GADD45β/MKK7 inhibitors display potent activity against MM, in vitro and in vivo
• GADD45β/MKK7 inhibitors are far more cancer selective than IKK/NF-κB inhibitors

## Teaser

NF-κB is implicated in MM and other malignancies, but it is challenging to block NF-κB only in diseased cells. Tornatore et al. identify an interaction between MKK7 and NF-κB-regulated GADD45β as a therapeutic target and develop a peptide that disrupts the complex and selectively kills MM cells.

## Significance

**NF-κB has been implicated in many inflammatory and malignant diseases, such as MM. Yet therapeutically targeting this pathway has proved an insurmountable challenge. The conundrum with current strategies has been how to block NF-κB in a disease-specific manner, given NF-κB’s pleiotropic and ubiquitous functions. Here, we have achieved this goal in the context of MM. Rather than targeting NF-κB, we targeted the downstream module, GADD45β/MKK7, within a pathogenically critical and cancer-restricted axis of the NF-κB pathway. We demonstrate that agents targeting this axis are both highly effective against MM and well tolerated in vivo, with far greater cancer cell specificity than global NF-κB inhibitors. Plausibly, the same principle could be applied for targeting NF-κB disease selectively also in pathologies beyond MM.**

## Introduction

In addition to orchestrating immune and inflammatory responses, NF-κB transcription factors play a crucial role in oncogenesis (Staudt, 2010). NF-κB is aberrantly activated in a wide range of human cancers, in which it promotes survival and malignancy by upregulating antiapoptotic genes (Staudt, 2010, DiDonato et al., 2012). The paradigm of these cancers is multiple myeloma (MM), an incurable malignancy of plasma cells (PCs), accounting for nearly 2% of all cancer deaths (Kuehl and Bergsagel, 2002). The current treatment for MM includes chemotherapy and steroids combined with newer agents, such as proteasome inhibitors and immunomodulatory drugs (IMiDs), whereas stem cell transplantation is an option for select patients. These treatments, however, generally achieve only temporary remissions, and so most patients eventually relapse and/or develop drug resistance (Rajkumar, 2011, Mahindra et al., 2012). Thus, despite the introduction of new treatments, the management of myeloma patients remains a major medical problem. Consequently, there is a need for more effective therapeutic approaches targeting defined oncogenetic events in MM.

Compelling evidence has established the paramount importance of aberrant NF-κB signaling in MM pathogenesis (Staudt, 2010, DiDonato et al., 2012). The most conclusive affirmation of this key role of NF-κB in MM has come from the discovery of a diverse array of genetic alterations targeting components of the NF-κB pathway, such as the upstream activator, NF-κB-inducing kinase and the inhibitor tumor necrosis factor receptor-associated factor 3, in about 20% of MM patients and more than 40% of MM cell lines (Annunziata et al., 2007, Keats et al., 2007, Demchenko et al., 2010, Chapman et al., 2011). Irrespective of their nature, these oncogenic lesions lead to constitutive activation of both main pathways of NF-κB signaling, namely, the classical and alternative pathways (Keats et al., 2007, Annunziata et al., 2007, Staudt, 2010, DiDonato et al., 2012). In fact, even in those patients with no recognizable NF-κB-pathway mutations, MM cells constitutively engage these pathways via stimuli emanating from the tumor microenvironment (Hideshima et al., 2005, Staudt, 2010). Consequently, more than 80% of all primary MM cells and the vast majority of MM cell lines display nuclear accumulation of NF-κB and high NF-κB target gene signature, leading to NF-κB-pathway addiction and sensitivity to apoptosis upon IκBα kinase (IKK) β/NF-κB inhibition (Staudt, 2010).

Collectively, these findings provide a strong rationale for therapeutically targeting the NF-κB pathway in MM. However, despite the pharmaceutical industry’s aggressive effort to develop specific NF-κB or IKKβ inhibitors for indication both within and outside of oncology, no such inhibitor has been clinically approved, because of the preclusive toxicities associated with the global suppression of NF-κB (DiDonato et al., 2012). Similarly, proteasome inhibitors with clinical indication in MM, such as bortezomib, inhibit many essential cellular pathways that rely on proteasome function, among which is the NF-κB pathway, and, furthermore, target these pathways in normal and cancer cells alike, thus resulting in a low therapeutic index and dose-limiting toxicities (Richardson, 2010, Chen et al., 2011). Indeed, it is unclear that the clinical activity of proteasome inhibitors in MM, as well as that of IMiDs, which too have broad molecular specificity and can affect NF-κB signaling, is due to the inhibition of NF-κB (Staudt, 2010, Chen et al., 2011, McCurdy and Lacy, 2013).

The conundrum with conventional NF-κB-targeting strategies has been how to achieve cancer cell specificity, given the ubiquitous nature and pleiotropic physiological functions of NF-κB (DiDonato et al., 2012). Because a key pathogenetic activity of NF-κB in MM is to block apoptosis through the induction of target genes, an attractive alternative to globally targeting NF-κB would be to block the nonredundant, cancer-specific downstream effectors of the NF-κB survival function; these effectors, however, are not known. To develop a strategy for inhibiting the NF-κB pathway in a cancer-selective manner and, thus, exploiting its therapeutic potential, we therefore sought to delineate the mechanism(s) underlying the pathological survival activity of constitutive NF-κB signaling in MM. Further, we sought to develop a pharmacological inhibitor of this mechanism(s) in order to kill MM cells effectively and without toxicity to normal cells.

## Results

### *GADD45B* Expression Denotes More Aggressive Disease in MM

**Figure 1:**
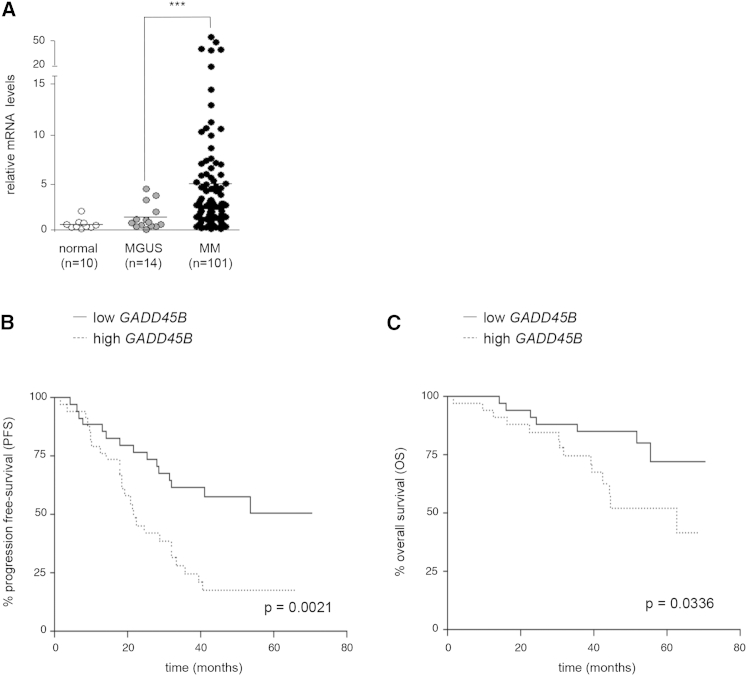
*GADD45B* Is Highly Expressed in MM Cells and Associates with Aggressive Disease (A) qRT-PCR showing the *GADD45B* mRNA levels in monoclonal CD138^+^ cells from MM or MGUS patients and polyclonal CD138^+^ cells (normal). ^∗∗∗^p = 0.00018. (B and C) Progression-free survival (B) and OS (C), as defined by the International Myeloma Working Group Uniform Response Criteria (Kyle and Rajkumar, 2009), in MM patients with low or high *GADD45B* levels, as assessed by qRT-PCR, in CD138^+^ cells. Patients were from the velcade/melphalan/prednisone arm of the trial and stratified at diagnosis, using as a cutoff the median *GADD45B* expression value. See also Figure S1.

Given their key role in oncogenesis, we sought to investigate the downstream mechanisms mediating NF-κB survival signaling in MM. Because this signaling involves the induction of antiapoptotic NF-κB target genes, and we had previously identified the *GADD45*-family gene, *GADD45B*, as a transcriptional target of NF-κB encoding a potent and selective inhibitor of the JNK MAPK pathway and, therefore, of apoptosis (De Smaele et al., 2001, Papa et al., 2004), we investigated the involvement of this gene in MM. *GADD45B* was markedly upregulated in monoclonal CD138^+^ PCs from MM patients compared with monoclonal PCs from patients with monoclonal gammopathy of undetermined significance (MGUS), a premalignant condition (Kyle and Rajkumar, 2009), or healthy polyclonal PCs (Figure 1A), thus establishing a correlation between *GADD45B* mRNA expression and PC malignancy. Strikingly, when MM patients were stratified at diagnosis on the basis of the *GADD45B* mRNA expression in CD138^+^ cells, the cohort of patients expressing high levels of *GADD45B* exhibited dramatically shorter progression-free survival and significantly shorter overall survival (OS) than the cohort of patients expressing low levels of *GADD45B*, despite both groups of patients having been treated with the same velcade/melphalan/prednisone protocol (Palumbo et al., 2010) (Figures 1B and 1C). A similar correlation of *GADD45B* expression with poor clinical outcome was observed using two independent gene expression data sets of newly diagnosed MM patients, thus providing external validation of our findings (Broyl et al., 2010, Dickens et al., 2010) (Figures S1A and S1B available online). Collectively, these results establish a strong correlation between *GADD45B* expression and disease progression in MM and identify GADD45β as a hallmark of more aggressive disease.

### Constitutive NF-κB Activity Promotes the Survival of MM Cells by Inhibiting JNK Signaling

GADD45β inhibits apoptosis by suppressing JNK signaling. It mediates this function by binding to the JNK kinase MKK7 and blocking its enzymatic activity by engaging the kinase catalytic pocket (Papa et al., 2004, Papa et al., 2007). This activity of GADD45β on the JNK pathway and the elevated *GADD45B* mRNA levels observed in monoclonal PCs from MM patients (Figure 1A) prompted us to investigate whether GADD45β mediated an antiapoptotic crosstalk between the NF-κB and JNK pathways in MM cells. We reasoned that cell extrinsic stimulation, as well as intrinsic oncogenic signals and NF-κB-pathway mutations, could result in the activation of signaling pathways beyond the IKK/NF-κB cascade, such as the JNK pathway (Davies and Tournier, 2012), which we and others have previously shown can trigger apoptosis in a manner that can be suppressed by NF-κB (De Smaele et al., 2001, Tang et al., 2001, Papa et al., 2004). Indeed, at least in principle, this mechanism could provide a basis for the addiction of MM cells to NF-κB for survival. In line with this hypothesis, in each of five heterogeneous MM cell lines and a B lymphoblastoid cell line, the silencing of the NF-κB subunit RelA (DiDonato et al., 2012) triggered JNK activation and apoptosis (Figures S2A–S2C), and blocking this activation with the JNK inhibitor SP600125 effectively protected NF-κB/RelA-silenced MM cells from cell death (Figures S2D and S2E). Similarly, the small hairpin RNA (shRNA)-mediated silencing of MKK7 rescued MM cells from the cytotoxic effects of each of two pharmacological inhibitors of IKKβ/NF-κB, and the protective effect of this silencing was virtually complete (Figure S2F). Collectively, these data indicate that constitutive NF-κB activity promotes the survival of MM cells by inhibiting MKK7/JNK signaling.

### GADD45β as a Pivotal Survival Factor Downstream of NF-κB and a Potential Therapeutic Target in MM

**Figure 2:**
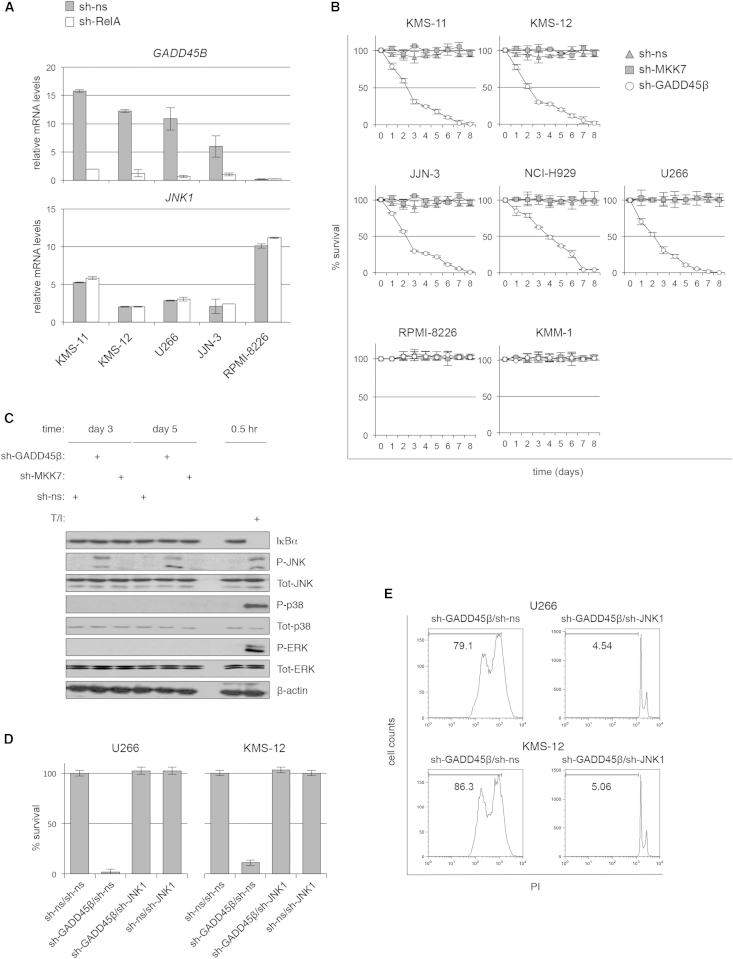
GADD45β Mediates the NF-κB Survival Activity in MM Cells by Suppressing JNK Signaling (A) qRT-PCR showing the *GADD45B* mRNA levels in MM cell lines expressing nonspecific (sh-ns) or RelA-specific (sh-RelA) shRNAs. *JNK1* is shown as control. Values denote mean ± SD (n = 3). (B) Survival of MM cell lines expressing sh-ns, GADD45β-specific (sh-GADD45β), or MKK7-specific (sh-MKK7) shRNAs. Values express the percentage of live eGFP^+^ cells relative to the number of live eGFP^+^ cells in the same culture on day 0. Values denote mean ± SD (n = 3). (C) Western blots showing total and phosphorylated (P) proteins in U266 MM cells from (B). T/I, TPA/ionomycin. (D) Trypan blue exclusion showing the survival of representative MM cell lines coexpressing sh-GADD45β or sh-ns and JNK1-specific (sh-JNK1) or sh-ns shRNAs on day 8 after lentivirus infection. Values denote mean ± SD (n = 3). (E) PI staining showing apoptotic cells (i.e., cells with sub-G_1_ DNA content) in representative MM cell lines from (D). The percentages of apoptotic cells are depicted. See also Figure S2.

To investigate the possible contribution of GADD45β to MM pathogenesis, we examined whether GADD45β mediated the NF-κB-dependent survival function and inhibition of JNK signaling in MM cells. As with PCs from patients (Figure 1A), *GADD45B* was expressed at high levels in MM cell lines compared with most other cancer cell lines tested (Figure S2G, discussed below). Importantly, this high *GADD45B* expression in MM cells markedly diminished upon the silencing of RelA, thus demonstrating its dependence on constitutive NF-κB activity (Figure 2A; Figure S2C). Moreover, similar to the effects of RelA-targeting hairpins (Figures S2A–S2C), the introduction of GADD45β-specific shRNAs, but not of MKK7-specific or of nonspecific shRNAs, induced potent JNK activation and apoptosis in all but two of the MM cell lines tested, namely, the RPMI-8226 and KMM-1 cell lines, which exhibited almost undetectable levels of GADD45β and significantly lower levels of MKK7 than those GADD45β-dependent MM cell lines (Figures 2B and 2C; Figures S2G–S2K, further discussed below). Similar results were observed using additional GADD45β-targeting hairpins, thus confirming the gene-silencing efficiency and specificity of the shRNAs used (Figures S2H, S2J, S2L, and S2M). Strikingly, the extent of JNK activation induced by the silencing of GADD45β in sensitive MM cell lines was similar to that observed with 12-O-tetradecanoylphorbol-13-acetate (TPA)/ionomycin stimulation, which potently induces JNK (Figure 2C; Figures S2I and S2J). By contrast, GADD45β downregulation had no effect on IKK/NF-κB, ERK, or p38 activity. As seen with the inhibition of IKKβ/NF-κB (Figures S2D and S2E), both the silencing of JNK1 and the treatment with SP600125 effectively reversed apoptosis in GADD45β-silenced MM cells (Figures 2D and 2E; Figures S2N–S2P). Hence, GADD45β promotes the survival of MM cells by inhibiting JNK-mediated apoptosis. Collectively, these findings identify GADD45β as an essential NF-κB-regulated survival factor and selective MKK7/JNK-axis inhibitor and, therefore, as a potential therapeutic target in MM.

### Development of D-Tetrapeptide Inhibitors of the GADD45β/MKK7 Complex

**Figure 3:**
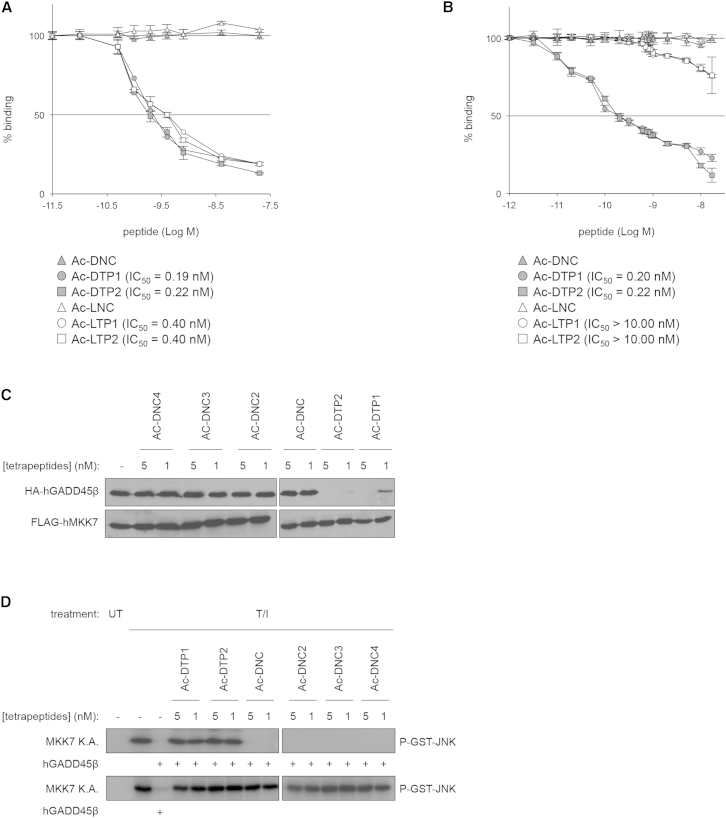
The Potent Activity and Stability of D-Peptide Inhibitors of GADD45β/MKK7 In Vitro (A and B) ELISA GADD45β/MKK7 competition assays showing the IC_50_ values of the active L-tetrapeptides (Ac-LTP1, Ac-LTP2) and D-tetrapeptides (Ac-DTP1, Ac-DTP2), before (A) and after (B) a 48 hr preincubation with human serum. Values express the percentage of inhibition of GADD45β binding to MKK7 relative to the binding in the absence of peptide and denote means ± SD (n = 3). Ac-LNC, acetylated (Ac) negative control L-tetrapeptide (LNC); Ac-DNC, Ac negative control D-tetrapeptide (DNC). (C) Coimmunoprecipitations (IP) performed with cell lysates prepared from human embryonic kidney 293T cells expressing ectopic HA-tagged GADD45β (HA-hGADD45β) and FLAG-tagged MKK7 (FLAG-hMKK7) and incubated with anti-FLAG antibody in the presence or absence of bioactive (Ac-DTP1 and Ac-DTP2) or inactive (Ac-DNC, Ac-DNC2, Ac-DNC3 and Ac-DNC4) D-tetrapeptides, as indicated. Western blots were developed using anti-HA or anti-MKK7 antibodies. -, incubation without D-tetrapeptides. (D) Kinase assays (K.A.) showing MKK7 activity before (-) and after incubation with Ac-DTP1, Ac-DTP2 or control D-tetrapeptides as in (C), in the presence (+) or absence of recombinant human (h)GADD45β. T/I, TPA/ionomycin; UT, untreated. See also Figure S3 and Tables S1 and S2.

Given the essential antiapoptotic role of GADD45β in MM and our previous results showing that GADD45β suppresses JNK signaling and apoptosis by blocking MKK7 via direct physical interaction (De Smaele et al., 2001, Papa et al., 2004, Papa et al., 2007), we aimed to develop selective inhibitors of this protein-protein interaction in order to induce cytotoxic JNK signaling in MM cells. We screened a simplified combinatorial library of 20,736 L-tetrapeptides to select compounds capable of disrupting the GADD45β/MKK7 complex (Figure S3A and Table S1). Iterative deconvolution of this library in ELISA competition assays, followed by secondary screening and optimization of the resulting hits, yielded two *ac*etylated L-*t*etra*p*eptides of similar structure, namely, Ac-LTP1 and Ac-LTP2, which disrupted the GADD45β/MKK7 complex, in vitro, with remarkable half-maximal inhibitory concentration (IC_50_) values in the subnanomolar range (Figure 3A; Figure S3B and Table S2), in line with the top-end potencies of other hits isolated from similar peptide library screens (Houghten et al., 1999).

Next, we examined whether these two L-tetrapeptides retained in vitro potency upon synthesis in the D configuration, a strategy used successfully in some cases to render small peptides resistant to proteolysis (Zhou et al., 2002, Nickl et al., 2010). Strikingly, as shown in Figures 3A and 3B, the D-enantiomers of Ac-LTP1 and Ac-LTP2 (termed Ac-DTP1 and Ac-DTP2, respectively) displayed no loss of activity in vitro and, unlike their L counterparts, were highly stable in human serum, even after prolonged incubation. Coimmunoprecipitation assays confirmed the potent and specific inhibitory effect of Ac-DTP1 and Ac-DTP2 on GADD45β/MKK7 complex stability in vitro (Figure 3C). By contrast, control D-tetrapeptides had no such effect on stability, thus demonstrating the specificity of the inhibitory activities of Ac-DTP1 and Ac-DTP2. Crucially, the disruption of the complex by these two active D-peptides completely reversed the GADD45β-mediated inhibition of MKK7, fully restoring the kinase catalytic activity (Figure 3D, top). Importantly, none of Ac-DTP1, Ac-DTP2, or any of the control D-tetrapeptides affected MKK7 activity in the absence of GADD45β (Figure 3D, bottom). Collectively, these findings identify a downstream, drug-targetable module in the NF-κB pathway and confirm the potential of our pharmacological approach for inducing cytotoxic MKK7/JNK signaling in cells that rely on GADD45β for restraining MKK7 activation.

**Figure 4:**
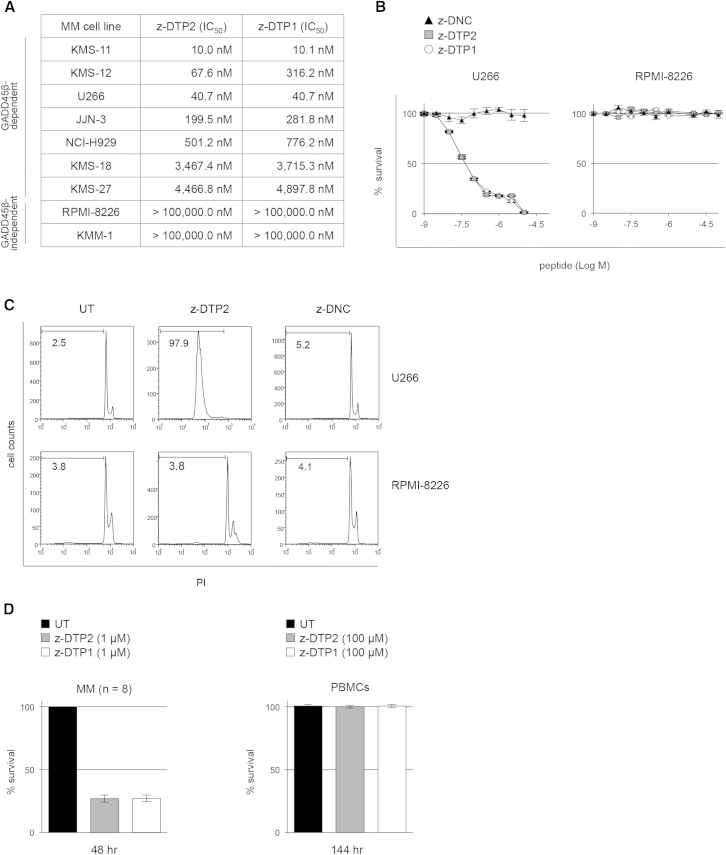
The Potent and Cancer-Selective Activity of D-Peptide Inhibitors of GADD45β/MKK7 in MM Cells (A) IC_50_ values of z-DTP1 and z-DTP2 at 144 hr, as determined by [^3^H]thymidine incorporation, in MM cell lines that depend or do not depend on GADD45β for survival. (B) [^3^H]Thymidine incorporation showing the survival of representative sensitive (U266) and resistant (RPMI-8226) MM cell lines from (A) after a 6-day treatment with the indicated concentrations of z-DTP1, z-DTP2, or Z-protected (z)-DNC. (C) PI staining showing apoptotic cells in representative MM cell lines from (B), after treatment with 10 μM of z-DTP1, z-DTP2 or z-DNC for 6 days. The percentages of apoptotic cells are indicated. (D) Trypan blue exclusion showing the survival of CD138^+^ cells from MM patients (n = 8) and healthy human PBMCs after treatment with z-DTP1 or z-DTP2 for 48 and 144 hr, respectively. (B and D) Values express the percentage of live cells present in the treated cultures relative to the live cells present in the respective untreated cultures, represented as 100%, and denote means ± SD (B), or SEM (D) [PBMCs] (n = 3), (D) [MM] (n = 8). See also Figure S4 and Table S3.

To verify the therapeutic potential of GADD45β/MKK7 inhibitors, we aimed to improve their cell penetration. We replaced the N-terminal acetyl group of Ac-DTP1 and Ac-DTP2 with a benzyloxycarbonyl [Z] group, thereby generating z-DTP1 and z-DTP2, respectively (Table S3), which retained high activity and stability in vitro (Figures S4A and S4B) and, importantly, also exhibited potent cytotoxic activity across a panel of genetically heterogeneous MM cell lines (Figures 4A and 4B; Figures S4C–S4F and Table S3). Significantly, z-DTP1 and z-DTP2, but not a control D-tetrapeptide, induced potent and dose-dependent toxicity in all of the MM cell lines tested, except the two expressing nearly undetectable levels of *GADD45B* and low levels of MKK7 (further discussed below), exhibiting IC_50_ values in the sensitive MM cell lines in the low nanomolar to low micromolar range (Figures 4A and 4B; Figures S2G, S4E, and S4F). Consistent with the protective mechanism mediated by GADD45β (Figure S2G), the z-DTP1- and z-DTP2-afforded killing of sensitive MM cells was due to the induction of apoptosis (Figure 4C; Figures S4G and S4H). Importantly, both active D-peptides retained potent and cancer-selective activity in primary PCs from MM patients (Figure 4D, left). Crucially, these D-peptides also exhibited an apparently complete lack of toxicity to normal cells, even when used at very high concentrations (i.e., 100 μM; Figure 4D, right; Figure S4I). Hence, D-tetrapeptide antagonists of the GADD45β/MKK7 complex show exceptionally high activity and cancer cell specificity in terms of apoptosis induction in MM cells, without displaying any apparent toxicity to normal cells.

### The Development of DTP3: A GADD45β/MKK7 Inhibitor with Improved Bioavailability

**Figure 5:**
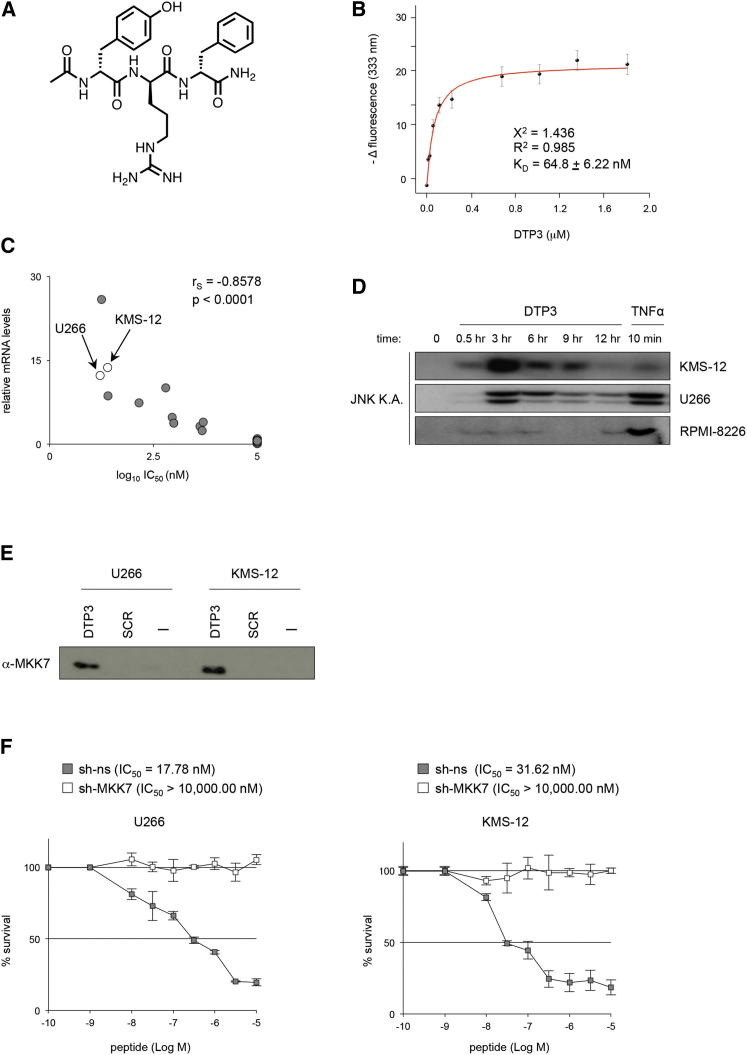
The High Target Specificity of DTP3 in Cells (A) The structure of DTP3. (B) Tryptophan fluorescence quenching analysis showing the dose-response curve of the -Δfluorescence values of GST-hMKK7 at 333 nm plotted against the concentration values of DTP3. The stoichiometry and K_D_ value of the DTP3/MKK7 interaction on the basis of these data were 1:1 and 64.81 ± 6.22 nM, respectively. Values denote means ± SD (n = 3). (C) Correlation plot of the relative *GADD45B* mRNA levels (qRT-PCR) and the DTP3 IC_50_ at 144 hr ([^3^H]thymidine incorporation) in cancer cell lines of different tissues of origin. Values on the x axis express the logarithm to base 10 (log_10_) of the IC_50_. r_S_, Spearman correlation coefficient. (D) K.A. showing JNK activity in representative sensitive (KMS-12, U266) and resistant (RPMI-8226) MM cell lines after treatment with DTP3 (10 μM). TNFα is shown as a positive control. (E) Peptide pull-down showing the physical association of DTP3 with endogenous MKK7 in U266 and KMS-12 MM cells. SCRB D-peptide is used as a negative control. -, pull-down without D-tripeptide. (F) [^3^H]Thymidine incorporation showing the survival of U266 and KMS-12 MM cells expressing sh-ns or sh-MKK7 shRNAs after a 6-day treatment with the indicated concentrations of DTP3. The IC_50_ values of DTP3 are depicted. Values express the percentage of the counts per minute (cpm) measured with the treated cultures relative to the cpm measured with the respective untreated cultures and denote mean ± SD (n = 3). See also Figure S5 and Tables S4 and S5.

To improve the bioavailability of D-peptides in vivo, while retaining high cellular activity and specificity toward the GADD45β/MKK7 complex, we used a chemical optimization strategy based on structure-activity relationship and pharmacophore analyses (Figures S5A–S5C and Table S4). By combining these methods, we developed DTP3, a D-tripeptide with a molecular weight of 525 Da (Figure 5A), which retained all the main characteristics of the parental D-tetrapeptides in terms of bioactivity and specificity, including subnanomolar activity and high stability in vitro and potent and selective capacity to kill MM cells via apoptosis (Figures S5D–S5H), while exhibiting a superior pharmacokinetic profile compared with the parent molecules (discussed below).

We further evaluated the modality and specificity of the binding of DTP3 to the GADD45β/MKK7 complex. Circular dichroism (CD) studies demonstrated that DTP3, but not a control scrambled (SCRB) D-tripeptide, can physically interact with and cause a significant conformational change of the structure of this complex, as well as of the isolated MKK7 protein, in a dose-dependent manner (Figure S5I). By contrast, DTP3 had no effect on the CD signal of GADD45β (Figure S5I). MALDI-TOF mass spectrometry confirmed the ability of DTP3 to bind to MKK7, but not to GADD45β (Figure S5J). Spectrofluorimetric analyses yielded similar results and, furthermore, established the 1:1 stoichiometry and low equilibrium dissociation constant (*K*_D_) value of the DTP3 interaction with MKK7 (Figure 5B; Figure S5K), thus underscoring the strength and specificity of this interaction. Conversely, the SCRB D-tripeptide had no impact on the fluorescence emission spectrum of MKK7, thus reaffirming the specificity of the effects of DTP3. Computational analyses predicted the presence of a pocket of MKK7, also found on the GADD45β/MKK7 complex, that can bind to DTP3 (Figures S5L–S5T). Collectively, these results demonstrate that DTP3 has the capacity to physically interact with MKK7, both in isolation and within the complex with GADD45β, and support a model whereby, upon binding to MKK7, DTP3 dissociates the GADD45β/MKK7 complex via an allosteric mechanism, potentially involving a conformational rearrangement of the kinase.

### The High Target Specificity of DTP3 for the GADD45β/MKK7 Complex

In a panel of tumor cell lines of different tissues of origin, the sensitivity to DTP3-induced killing correlated with a very high degree of significance with the mRNA levels of *GADD45B* (Figure 5C; Figures S2G and S5U), which our data have shown is a pivotal NF-κB-regulated inhibitor of MKK7/JNK signaling and apoptosis in MM cells (Figures 2B–2E; Figures S2H–S2J and S2N–S2P). Significantly, DTP3 displayed potent and selective activity in both MM and non-MM cell lines exhibiting high levels of *GADD45B* expression, whereas it was completely inactive, even at high micromolar concentrations, in tumor cell lines featuring low *GADD45B* expression (Figures S2G and S5F–S5H). In the context of MM, the RPMI-8226 and KMM-1 cell lines, which express very low levels of GADD45β and low levels of MKK7 and are consequently unaffected by GADD45β-targeting hairpins, were completely refractory to DTP3-induced killing (Figure 2B; Figures S2G–S2I, S2K, and S5F–S5H).

As seen with GADD45β-silencing shRNAs (Figure 2C; Figures S2H and S2I), treatment with DTP3 effectively activated JNK but not p38, ERK, or IKK/NF-κB signaling in sensitive, GADD45β-dependent MM cell lines, whereas it did not affect any of these pathways in DTP3-resistant, GADD45β-independent MM cell lines, such as RPMI-8226 (Figure 5D; Figure S5V; see also Figure S4H, z-DTP2). Strikingly, the magnitude of the effects of DTP3 on the JNK pathway was similar to that observed with tumor necrosis factor α (TNFα), a potent inducer of JNK (Figure 5D; Figures S4H and S5V, TPA/ionomycin). By contrast, a negative control D-peptide had no effect on JNK signaling (Figure S5V). As expected from the in vitro data (Figure 5B; Figure S5I, middle, bottom; Figure S5J, left), these activating effects of DTP3 on the JNK pathway hinged upon the ability of this D-tripeptide to bind to MKK7 in cells, as shown by DTP3 pull-down assays (Figure 5E). These effects were not observed with the SCRB control peptide, nor were they observed with a pull-down control in the absence of peptide, thus demonstrating the specificity of the binding of DTP3 to endogenous MKK7. Importantly, knocking down the GADD45β target kinase, MKK7, or its downstream effector, JNK1, completely abolished the cytotoxic activity of DTP3 in sensitive MM cell lines (Figure 5F; Figures S5W and S5X), thus excluding any off-target toxicity of DTP3 in cells. DTP3 also lacked any off-target effect when profiled in kinase assays against a panel of 142 human kinases (Table S5). Hence, bioactive D-peptides selectively induce apoptosis in MM cells with functional MKK7 and elevated GADD45β expression by activating JNK signaling via MKK7. These findings also correlate the pharmacological activity of DTP3 with a tumor cell dependence on GADD45β for survival and establish the exceptionally high target specificity of this D-tripeptide for the GADD45β/MKK7 complex in cells.

### The Potent and Cancer-Selective Activity of DTP3 in MM Cells from Patients

**Figure 6:**
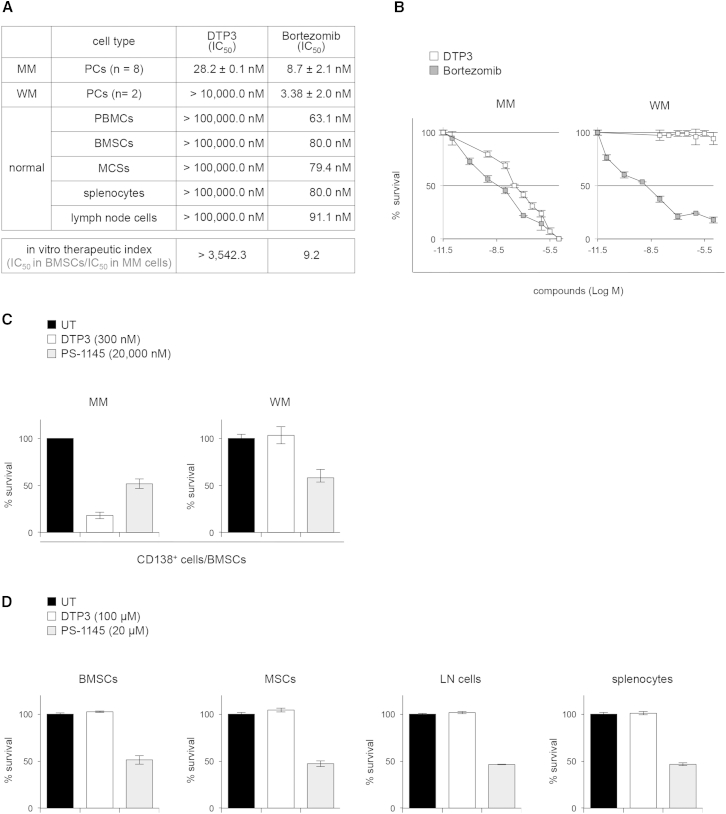
The Potent Activity of DTP3 in Primary MM Cells and Its Far Superior Cancer Cell Selectivity Compared with IKKβ and Proteasome Inhibitors (A) IC_50_ values of DTP3 and bortezomib at 48 hr in CD138^+^ cells from MM patients (n = 8), CD138^+^ cells from WM patients (n = 2), human PBMCs, human BMSCs, human mesenchymal stem cells (MSCs), and mouse splenocytes and lymph node (LN) cells (top), as determined by trypan blue exclusion. In vitro therapeutic indices (IC_50_ in BMSCs/IC_50_ in MM PCs) are depicted (bottom). MM and WM PC values denote means ± SEM. Other values denote means (n = 3). (B) Trypan blue exclusion showing the survival of CD138^+^ cells from representative patients from (A), after treatment with DTP3 or bortezomib for 48 hr. (C) Trypan blue exclusion showing the survival of CD138^+^ cells from MM or WM patients after treatment with DTP3 (300 nM) or PS-1145 (20 μM) for 48 hr, in the presence of BMSCs. (D) Trypan blue exclusion showing the survival of primary human BMSCs, human MSCs, and mouse LN cells and splenocytes after treatment with DTP3 (100 μM) or PS-1145 (20 μM) for 144 hr. (B–D) Values express the percentage of live cells present in the treated cultures relative to the live cells present in the respective untreated cultures and denote means ± SD (B), (C) [WM], (D) (n = 3) or SEM (C) [MM] (n = 9). See also Figure S6.

To verify the suitability of DTP3 for the treatment of human diseases, we tested its activity and selectivity in MM PCs from patients. As seen with the active D-tetrapeptides (Figure 4D, left), DTP3 effectively killed primary MM PCs at low nanomolar concentrations (Figures 6A and 6B). Importantly, it retained potent and selective cytotoxic activity in these cells upon stimulation with interleukin-6 or insulin growth factor 1 or coculture with bone marrow stromal cells (BMSCs) (Figure 6C; Figures S6A and S6B), which promote the survival of MM cells (Hideshima et al., 2005). In order to compare the efficacy and cancer cell specificity of DTP3 with the corresponding parameters of bortezomib, the current gold-standard treatment for MM (Chen et al., 2011), we defined an “in vitro therapeutic index” (Figure 6A, bottom). As shown in Figure 6A (top), DTP3 had a similar IC_50_ value to bortezomib in primary MM PCs but, importantly, unlike bortezomib, which barely discriminated between malignant and normal cells, had no toxicity to normal cells. Strikingly, because of this cancer cell selective target specificity, DTP3 had an in vitro therapeutic index that was greater, by more than two orders of magnitude, than that of bortezomib (Figure 6A, bottom). DTP3, in fact, could also distinguish between PCs from MM and Waldenström’s macroglobulinemia (WM) patients (Kyle and Rajkumar, 2009), in line with the *GADD45B* expression levels in these cells, whereas bortezomib could not (Figures 6A and 6B; Figure S6C). Similarly, DTP3 displayed far more potent activity in primary MM PCs and far less toxicity to normal cells than the IKKβ inhibitor, PS-1145 (Figures 6C and 6D; Figures S6A and S6B; note the different concentrations of DTP3 and PS-1145 used). Because the standard MM treatment consists of combination therapy, and nearly all patients will relapse and/or develop drug resistance at some point, we evaluated the potential of DTP3 to operate in these settings. As shown in Figures S6D and S6E, DTP3 displayed synergistic activity with bortezomib in two different MM cell lines, exhibiting a combination index of 0.21 in U266 cells and of 0.56 in KMS-12 cells, suggesting that it could find indication in the clinic in combination with bortezomib (Chou, 2006). Importantly, DTP3 also retained full therapeutic efficacy in MM cell lines that were resistant to conventional MM treatments, such as dexamethasone, bortezomib, and lenalidomide (Bjorklund et al., 2011, Bjorklund et al., 2014, Rückrich et al., 2009) (Figures S6F and S6G). Together, these results provide compelling evidence of the high therapeutic potential of DTP3 in MM patients.

### The Potent Therapeutic Efficacy and Excellent Tolerability of DTP3 against MM In Vivo

**Figure 7:**
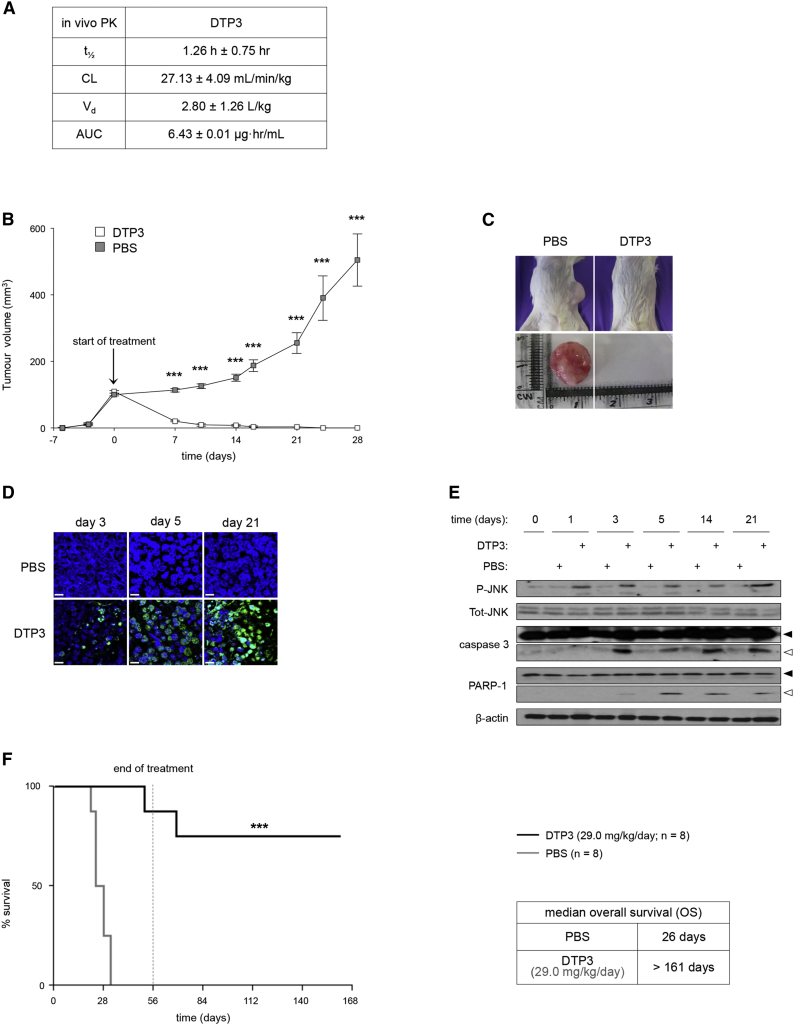
DTP3 Exhibits Potent Therapeutic Activity against MM, In Vivo, in the Absence of Any Apparent Side Effects (A) Pharmacokinetic (PK) values of DTP3 after single intravenous injection at the dose of 10 mg/kg. AUC, area under the plasma concentration versus time curve; CL, plasma clearance; t_1/2_, terminal half-life; V_d_, volume of distribution. Values denote means ± SD (n = 3). (B) Volumes of subcutaneous U266 myeloma xenografts in mice treated by continual infusion with DTP3 at a dose of 14.5 mg/kg/day or PBS for the times shown. Values denote means ± SEM (n = 16). ^∗∗∗^p < 0.001. (C) Images of representative myeloma-bearing mice (top) and isolated tumors (bottom) from (B) at day 28. (D) Images of TUNEL assays showing apoptotic cells in representative tumors from (B). Scale bars represent 10 μM. Green, TUNEL; blue, DAPI. (E) Western blots showing total and phosphorylated (P) JNK, and the unprocessed (filled arrowheads) and cleaved (open arrowheads) forms of caspase-3 and its proteolytic substrate, PARP-1, in representative tumors from (B). (F) Percentage survival of mice bearing medullary KMS-12 MM xenografts and treated intermittently by infusion with DTP3 at a dose of 29.0 mg/kg/day or PBS (left; n = 8, each group) for 8 weeks. Also shown is the median OS of each animal cohort (right). ^∗∗∗^p < 0.0001. See also Figure S7 and Table S6.

DTP3 showed high aqueous solubility and very high stability in human serum, owing to its resistance to proteolysis, with a good pharmacokinetic profile and excellent in vivo tolerability, suitable for a therapeutic purpose (Figure 7A; Figure S5D and Table S6).

Remarkably, in a plasmacytoma model, treatment with DTP3 at the dose of 14.5 mg/kg/day virtually eradicated established subcutaneous myeloma xenografts in mice, in the absence of any apparent side effects (Figures 7B and 7C; Figure S7A and Table S6). Similar results were obtained in a second plasmacytoma model, generated using a different MM cell line (Figures S7C and S7D). At the experimental end point, on day 28, all the control mice had developed large local tumors, whereas all the mice in the DTP3-treated cohort had shown a dramatic shrinkage of the tumors (Figures 7B and 7C; Figures S7C and S7D). This therapeutic effect of DTP3 was due to the potent and tumor-selective induction of JNK activation and apoptosis (Figures 7D and 7E; Figure S7B), as shown by the appearance of phosphorylated JNK, as early as 24 hr of the onset of treatment with DTP3, but not with PBS, followed by the appearance, starting on day 3, of caspase-3 and PARP-1 proteolysis products. Coincident with these events, apoptotic cells became evident in the tumor tissue at day 3 (Figure 7D; Figure S7B). As well as the tumor-ablative effects of DTP3 (Figure 7B; Figure S7A), the extent of this JNK-associated, tumor cell apoptosis markedly increased in magnitude over time (Figure 7D; Figure S7B).

**Figure 8:**
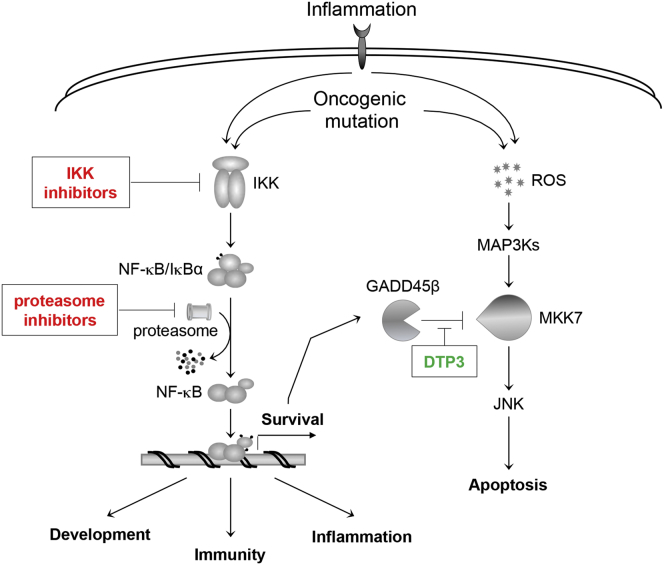
Schematic Representation of the Therapeutic GADD45β/MKK7-Targeting Strategy in Cancer Depicted is the crosstalk between the NF-κB and JNK pathways promoting the survival of cancer cells and cells exposed to inflammatory stimuli. Also illustrated is the NF-κB-dependent, Gadd45β/MKK7-mediated mechanism suppressing apoptotic JNK signaling in MM cells. Our therapeutic strategy to block the NF-κB survival function in a cancer-selective manner with DTP3 (green) is compared with conventional therapeutic approaches (e.g., IKKβ and proteasome inhibitors; red) also aimed at inhibiting the NF-κB pathway in cancer. MAP3Ks, mitogen-activated protein kinase kinase kinases; ROS, reactive oxygen species.

Importantly, DTP3 retained potent anticancer activity in an orthotopic xenograft model of MM, which more faithfully recapitulates the human disease. All the control mice developed severe limb paralysis and died within 32 days of treatment start, resulting in a median OS of 26 days (Figure 7F). Strikingly, DTP3 administration over a period of 8 weeks, at a dose of 29 mg/kg/day, extended the median OS of the mice past the experimental end point on day 161, without producing any apparent side effect, thus demonstrating the potent therapeutic efficacy of DTP3 against MM, in vivo, and the excellent tolerability of this agent at doses that achieve full therapeutic efficacy. Collectively, together with the data in primary MM cells (Figures 6A–6D; Figures S6A and S6B), these results underscore the potency, safety, and cancer cell specificity of the pharmacological approach targeting the GADD45β/MKK7 complex in MM and identify DTP3 as a therapeutic selectively inhibiting the NF-κB survival pathway in cancer (Figure 8).

## Discussion

We identified the GADD45β/MKK7 complex as a functionally critical survival module downstream of NF-κB and a therapeutic target in MM. Further, we developed a corresponding D-tripeptide inhibitor of this complex, DTP3, which effectively kills MM cells by inducing MKK7/JNK-dependent apoptosis and, at the same time, does not appear to be toxic to normal tissues. Because of this cancer cell specificity, DTP3 displays an exceptionally high therapeutic index in vitro and potent and cancer-selective activity against MM in vivo. Future studies will clarify the precise mechanism by which DTP3 dissociates the GADD45β/MKK7 complex. Notwithstanding, our findings uncover a mechanism for the pathogenetic survival activity of NF-κB in MM. Crucially, they also demonstrate that cancer-selective inhibition of the NF-κB survival pathway is possible and provide a promising therapy with no preclusive toxicity that could be of profound benefit for patients with this cancer and, potentially, others for which NF-κB promotes survival via GADD45β (Lam et al., 2005, Ngo et al., 2006, Tracey et al., 2005).

The therapeutic targeting of the NF-κB pathway has been aggressively pursued for the treatment of a wide range of inflammatory and malignant pathologies, including MM (DiDonato et al., 2012). However, it has proved so far an insurmountable challenge. Current therapeutic approaches, such as IKKβ inhibitors, target core components of this pathway, and so, although they are potentially capable of abrogating the cancer-promoting activities of NF-κB, they fail to preserve its pleiotropic physiological functions, such as functions in immunity and inflammation (Staudt, 2010, DiDonato et al., 2012). Because the best documented function of NF-κB in cancer is to induce genes that block apoptosis and, despite its ubiquitous nature, NF-κB signaling elicits highly tissue- and context-specific transcriptional programs (DiDonato et al., 2012), we sought to develop a therapeutic approach capable of inhibiting the NF-κB antiapoptotic activity, in a cancer-selective manner. We reasoned that agents targeting a nonredundant, downstream module within this critical survival axis of the NF-κB pathway and having functional restriction to the cancer cells (e.g., the GADD45β/MKK7 module in the case of MM) would provide a more selective and, therefore, more effective therapy, lacking the dose-limiting toxicities of conventional drugs globally targeting NF-κB. It is noteworthy in this regard that most normal cells do not express *GADD45B* constitutively (Zhang et al., 2005). Furthermore, unlike mice lacking RelA or any subunit of the IKK complex (DiDonato et al., 2012), *Gadd45b*^−/−^ mice are viable, are fertile, and die of old age (Papa et al., 2008, Lu et al., 2004), indicating that, in contrast to global NF-κB blockade, complete GADD45β inactivation is well tolerated in vivo.

Our approach also aims at exploiting the general good safety of peptide therapeutics and the greater selectivity afforded by inhibiting a protein-protein interaction, rather than the entire function of a receptor or an enzyme, as is the case for most drugs. Because of this property and their unique mode of action, it is expected that the side effects of GADD45β/MKK7 inhibitors in patients will be milder than the phenotypes of *Gadd45b*^−/−^ mice (Papa et al., 2008, Lu et al., 2004), both because of the transient nature of chemotherapeutic treatments and because these agents would enable GADD45β to retain its MKK7-independent functions. The partially impaired T_H_1 T cell response reported in *Gadd45b*^−/−^ mice (Lu et al., 2004), for example, was shown to depend on the Gadd45β-afforded regulation of the MAP3K, MEKK4 (Chi et al., 2004), but not of MKK7. Similarly, such agents would enable MKK7 to retain its enzymatic function and GADD45β-independent modalities of regulation. Nevertheless, future studies will be required to address the potential side effects of DTP3 in patients.

Many cancers beyond MM rely on constitutive NF-κB signaling for survival (Staudt, 2010, DiDonato et al., 2012). Together, the high expression of *GADD45B* in a subset of these cancers, including diffuse large B cell lymphoma (DLBCL) and other types of lymphoma (Lam et al., 2005, Ngo et al., 2006, Tracey et al., 2005), and the selective toxicity of DTP3 in DLBCL, Burkitt’s lymphoma and promonocytic leukemia cell lines, imply that GADD45β/MKK7 antagonists may have broader therapeutic potential beyond MM, in other areas of unmet need within oncology. By contrast, certain MM cell lines express low levels of GADD45β, as well as of MKK7, and are refractory to DTP3-induced killing. This suggests the existence of GADD45β-independent mechanisms for NF-κB-dependent survival in certain subtypes of MM and, most likely, in other types of malignancy. For instance, BCL-2 family members are possible mediators of such mechanisms, at least in the context of MM (Gomez-Bougie and Amiot, 2013). Therefore, on the basis of the principle we describe here of targeting an axis of the NF-κB pathway with cancer-restricted function, rather than NF-κB globally, delineating these mechanisms could provide new strategies for targeting NF-κB in a disease-specific manner also in GADD45β-independent, NF-κB-addicted malignancies and, perhaps, nonmalignant NF-κB-driven pathologies.

## Experimental Procedures

### Cell Purification and Culture

Cells were cultured according to standard protocols (Mauro et al., 2011, Piva et al., 2008). BMSCs and peripheral blood mononuclear cells (PBMCs) were purified from MM patients or the blood of healthy volunteers, respectively, as reported in Piva et al. (2008). CD138^+^ cells were purified from the bone marrow aspirates of patients using CD138 MicroBeads (Miltenyi Biotech). Patients were recruited at the Hematology Division of “Ospedale San Giovanni Battista” (Turin, Italy) and the Haematology Clinic at Imperial College Healthcare NHS Trust, under the approval of the Ethics Committee of “Ospedale San Giovanni Battista” (VMP-VMPT trial 163) and the London Harrow Research Ethics Committee (11/LO/1628), respectively. Written consent was documented for all subjects. Additional details are provided in Supplemental Experimental Procedures.

### Biochemical Assays

Quantitative RT-PCR (qRT-PCR) assays were carried out using the TaqMan Gene Expression Assays kit (Applied Biosystems). The kinase profiling of DTP3 across 142 human kinases was outsourced. Western blots, coimmunoprecipitations, and kinase assays were performed as described previously (Papa et al., 2004, Papa et al., 2008, Mauro et al., 2011). Additional details are provided in Supplemental Experimental Procedures.

### Cellular Assays

Lentiviral infections were performed using pLentiLox.3.7, as described by Mauro et al. (2011). Enhanced GFP (eGFP)^+^ cells were purified, when necessary, by fluorescence-activated cell sorting. [^3^H]Thymidine incorporation and trypan blue exclusion assays were performed using standard methods (Mauro et al., 2011). IC_50_ values were defined as the mean concentration of compound inducing 50% inhibition of cell viability relative to the viability in the untreated cultures. Apoptosis analyses were performed using propidium iodide (PI) staining, as described by Mauro et al. (2011). Additional details are provided in Supplemental Experimental Procedures.

### Peptide Synthesis and ELISA

Proteins were purified as described by Tornatore et al. (2008). The combinatorial L-tetrapeptide libraries and individual peptides were synthesized as reported by Sandomenico et al. (2012). The methods used to assess peptide identity and purity and deconvolute the tetrapeptide libraries and the ELISA GADD45β/MKK7 competition assays were described previously (Sandomenico et al., 2012, Tornatore et al., 2008). IC_50_ values were defined as the mean concentration of peptide inducing 50% inhibition of GADD45β binding to MKK7 relative to the binding measured in the absence of peptide. Additional details are provided in Supplemental Experimental Procedures.

### DTP3 Binding Assays

The stoichiometry and *K*_D_ value of the DTP3/MKK7 interaction were determined by tryptophan fluorescence quenching analysis, after fitting the fluorescence data with a nonlinear regression algorithm, as described by Williamson (2013). Additional details are provided in Supplemental Experimental Procedures, along with a description of the methods used for CD and MALDI-TOF mass spectrometry analyses and the pull-down of MKK7 from cells with DTP3.

### Pharmacophore Analyses and Modeling

See Supplemental Experimental Procedures.

### Pharmacokinetic Analyses

The pharmacokinetic analyses of DTP3 and z-DTP2 were outsourced. Additional details are provided in Supplemental Experimental Procedures.

### Animal Studies

Mice were housed in the animal facilities at Hammersmith. All experiments were conducted under Procedure Project License (PPL) 70/6874, after approval by the Imperial College Ethical Review Process and the Home Office. For the plasmacytoma model, nonobese diabetic/severe combined immunodeficiency mice (NOD.CB17-Prkdc^scid^/IcrCrl; Charles River) were injected subcutaneously with 1.0 × 10^7^ U266 or KMS-11 MM cells and then randomized into treatment groups and treated by infusion, as shown. Tumor volumes were measured as described by Mauro et al. (2011). For the orthotopic MM model, mice of the same strain were sublethally irradiated and then injected intravenously with 1.0 × 10^7^ KMS-12-BM MM cells, as described by Rabin et al. (2007). Mice were then randomized into treatment groups and treated by infusion for 8 weeks, as shown. Animals were monitored daily and euthanized on day 161 of treatment start or when they reached any of the end points in the PPL. Additional details are provided in Supplemental Experimental Procedures.

### Statistical Analyses

See Supplemental Experimental Procedures.

## Author Contributions

L.T., G.F., M.R., D.R., C.L. and A.R. designed experiments. L.T., A.S., D.R., C.L., D.C., A.K.T., and A.C. performed experiments. L.T., G.F., M.R., A.S., D.R., C.L., A.R., C.T.-S., D.C., D.D., E.B., M.v.D., P.Z., A.J.-C., A.C., A.T., and A.R. analyzed data. M.B., B.A.W., A.L., C.D., P.S., G.M., A.P., and A.R. contributed clinical samples or key reagents. L.T., A.K.T., and J.D. contributed to mouse studies. G.F. wrote the paper. M.R., L.T., D.R., C.L., A.R., and A.T. contributed to writing the paper. A.S., D.R., and C.L. contributed equally to this work.

## Supplemental Information

Document S1. Supplemental Experimental Procedures, Figures S1–S7, Tables S1–S6

Document S2. Article plus Supplemental Information
