# Cancer-Selective Targeting of the NF-κB Survival Pathway with GADD45β/MKK7 Inhibitors

**DOI:** 10.1016/j.ccell.2014.11.021

**Published:** 2014-12-08

**Authors:** Laura Tornatore, Annamaria Sandomenico, Domenico Raimondo, Caroline Low, Alberto Rocci, Cathy Tralau-Stewart, Daria Capece, Daniel D’Andrea, Marco Bua, Eileen Boyle, Mark van Duin, Pietro Zoppoli, Albert Jaxa-Chamiec, Anil K. Thotakura, Julian Dyson, Brian A. Walker, Antonio Leonardi, Angela Chambery, Christoph Driessen, Pieter Sonneveld, Gareth Morgan, Antonio Palumbo, Anna Tramontano, Amin Rahemtulla, Menotti Ruvo, Guido Franzoso

(Cancer Cell *26*, 495–508; October 13, 2014)

Following publication of this manuscript, the authors discovered some panels that had been incorrectly cropped in Figures S4H and S5V in the Supplemental Information.

As a result of this error, the 0 hr time points of the top PARP-1 panel of Figure S4H, U266, the Tot-JNK and Tot-ERK panels of Figure S5V, U266, and the P-p38 panel of Figure S5V, RPMI-8226, as well as the 0 hr and 0.5 hr, DTP3, time points of the P-ERK panel of Figure S5V, RPMI-8226, were accidentally omitted from these panels. An extra time point (i.e., a 1.5 hr time point, T/I) was also accidentally included in the P-ERK panel of this figure. Due to an analogous error, the P-JNK panel of Figure S5V, RPMI-8226, was incorrectly aligned.

The authors also discovered an antibody that had been erroneously omitted from the Supplemental Experimental Procedures. This antibody, anti-PARP-1 (9532S; 1:1,000), should have been included in the section “Antibodies, Reagents and Western Blots.”

A corrected version of the Supplemental Information is now available online.

