# Supplementary material for: Cancer-Selective Targeting of the NF-κB Survival Pathway with GADD45β/MKK7 Inhibitors
Source: Cancer Cell. 2014 Oct 13;26(4):495–508. doi: 10.1016/j.ccr.2014.07.027 (PMC4197335; doi:10.1016/j.ccr.2014.07.027)
Supplement: Document S2. Article plus Supplemental Information [file mmc2.pdf]

Cancer Cell
Article

Cancer-Selective Targeting of the NF-κB Survival Pathway with GADD45β/MKK7 Inhibitors

Laura Tornatore,[1] Annamaria Sandomenico,[2] Domenico Raimondo,[3] Caroline Low,[4] Alberto Rocci,[5] Cathy Tralau-Stewart,[4] Daria Capece,[1] Daniel D'Andrea,[3] Marco Bua,[6] Eileen Boyle,[7] Mark van Duin,[8] Pietro Zoppoli,[9] Albert Jaxa-Chamiec,[4] Anil K. Thotakura,[1] Julian Dyson,[10] Brian A. Walker,[7] Antonio Leonardi,[11] Angela Chambery,[12,13] Christoph Driessen,[14] Pieter Sonneveld,[8] Gareth Morgan,[7] Antonio Palumbo,[5] Anna Tramontano,[3,15] Amin Rahemtulla,[6] Menotti Ruvo,[2,*] and Guido Franzoso[1,*]

[1]Department of Medicine, Centre for Cell Signalling and Inflammation, Imperial College London, London W12 0NN, UK
[2]Institute of Biostructures and Bioimages, National Research Council and CIRPeB, 80134 Naples, Italy
[3]Department of Physics, "Sapienza" University, 00185 Rome, Italy
[4]Drug Discovery Centre, Imperial College London, London W6 8RP, UK
[5]Division of Hematology, University of Torino, AOU San Giovanni Battista, 10126 Turin, Italy
[6]Department of Medicine, Centre for Haematology, Imperial College London, London W12 0NN, UK
[7]Section of Haemato-Oncology, The Institute of Cancer Research, London SM2 5NG, UK
[8]Department of Hematology, Erasmus University Medical Center, 3000 CA Rotterdam, the Netherlands
[9]Institute for Cancer Genetics, Columbia University Medical Center, New York, NY 10032, USA
[10]Department of Medicine, Section of Molecular Immunology, Imperial College London, London W12 0NN, UK
[11]Department of Molecular Medicine and Medical Biotechnologies, University of Naples "Federico II," 80131 Naples, Italy
[12]Department of Environmental, Biological, and Pharmaceutical Sciences and Technologies, Second University of Naples, 81100 Caserta, Italy
[13]IRCCS Multimedica, 20138 Milan, Italy
[14]Department of Oncology/Hematology, Kantonsspital St. Gallen, 9007 St. Gallen, Switzerland
[15]Istituto Pasteur Fondazione Cenci Bolognetti, "Sapienza" University, 00185 Rome, Italy
*Correspondence: menotti.ruvo@unina.it (M.R.), g.franzoso@imperial.ac.uk (G.F.)
http://dx.doi.org/10.1016/j.ccr.2014.07.027

SUMMARY

Constitutive NF-κB signaling promotes survival in multiple myeloma (MM) and other cancers; however, current NF-κB-targeting strategies lack cancer cell specificity. Here, we identify the interaction between the NF-κB-regulated antiapoptotic factor GADD45β and the JNK kinase MKK7 as a therapeutic target in MM. Using a drug-discovery strategy, we developed DTP3, a D-tripeptide, which disrupts the GADD45β/MKK7 complex, kills MM cells effectively, and, importantly, lacks toxicity to normal cells. DTP3 has similar anticancer potency to the clinical standard, bortezomib, but more than 100-fold higher cancer cell specificity in vitro. Notably, DTP3 ablates myeloma xenografts in mice with no apparent side effects at the effective doses. Hence, cancer-selective targeting of the NF-κB pathway is possible and, at least for myeloma patients, promises a profound benefit.

INTRODUCTION

In addition to orchestrating immune and inflammatory responses, NF-κB transcription factors play a crucial role in oncogenesis (Staudt, 2010). NF-κB is aberrantly activated in a wide range of human cancers, in which it promotes survival and malignancy by upregulating antiapoptotic genes (Staudt, 2010; DiDonato et al., 2012). The paradigm of these cancers is multiple myeloma (MM), an incurable malignancy of plasma cells (PCs), accounting for nearly 2% of all cancer deaths (Kuehl and Bergsagel, 2002). The current treatment for MM includes chemotherapy and steroids combined with newer agents, such as proteasome inhibitors and immunomodulatory drugs (IMiDs), whereas stem cell transplantation is an option for select

Significance

NF-κB has been implicated in many inflammatory and malignant diseases, such as MM. Yet therapeutically targeting this pathway has proved an insurmountable challenge. The conundrum with current strategies has been how to block NF-κB in a disease-specific manner, given NF-κB's pleiotropic and ubiquitous functions. Here, we have achieved this goal in the context of MM. Rather than targeting NF-κB, we targeted the downstream module, GADD45β/MKK7, within a pathogenically critical and cancer-restricted axis of the NF-κB pathway. We demonstrate that agents targeting this axis are both highly effective against MM and well tolerated in vivo, with far greater cancer cell specificity than global NF-κB inhibitors. Plausibly, the same principle could be applied for targeting NF-κB disease selectively also in pathologies beyond MM.

 ©2014 Elsevier Inc.

patients. These treatments, however, generally achieve only temporary remissions, and so most patients eventually relapse and/or develop drug resistance (Rajkumar, 2011; Mahindra et al., 2012). Thus, despite the introduction of new treatments, the management of myeloma patients remains a major medical problem. Consequently, there is a need for more effective therapeutic approaches targeting defined oncogenetic events in MM.

Compelling evidence has established the paramount importance of aberrant NF-κB signaling in MM pathogenesis (Staudt, 2010; DiDonato et al., 2012). The most conclusive affirmation of this key role of NF-κB in MM has come from the discovery of a diverse array of genetic alterations targeting components of the NF-κB pathway, such as the upstream activator, NF-κB-inducing kinase and the inhibitor tumor necrosis factor receptor-associated factor 3, in about 20% of MM patients and more than 40% of MM cell lines (Annunziata et al., 2007; Keats et al., 2007; Demchenko et al., 2010; Chapman et al., 2011). Irrespective of their nature, these oncogenic lesions lead to constitutive activation of both main pathways of NF-κB signaling, namely, the classical and alternative pathways (Keats et al., 2007; Annunziata et al., 2007; Staudt, 2010; DiDonato et al., 2012). In fact, even in those patients with no recognizable NF-κB-pathway mutations, MM cells constitutively engage these pathways via stimuli emanating from the tumor microenvironment (Hideshima et al., 2005; Staudt, 2010). Consequently, more than 80% of all primary MM cells and the vast majority of MM cell lines display nuclear accumulation of NF-κB and high NF-κB target gene signature, leading to NF-κB-pathway addiction and sensitivity to apoptosis upon IκBα kinase (IKK) β/NF-κB inhibition (Staudt, 2010).

Collectively, these findings provide a strong rationale for therapeutically targeting the NF-κB pathway in MM. However, despite the pharmaceutical industry's aggressive effort to develop specific NF-κB or IKKβ inhibitors for indication both within and outside of oncology, no such inhibitor has been clinically approved, because of the preclusive toxicities associated with the global suppression of NF-κB (DiDonato et al., 2012). Similarly, proteasome inhibitors with clinical indication in MM, such as bortezomib, inhibit many essential cellular pathways that rely on proteasome function, among which is the NF-κB pathway, and, furthermore, target these pathways in normal and cancer cells alike, thus resulting in a low therapeutic index and dose-limiting toxicities (Richardson, 2010; Chen et al., 2011). Indeed, it is unclear that the clinical activity of proteasome inhibitors in MM, as well as that of IMiDs, which too have broad molecular specificity and can affect NF-κB signaling, is due to the inhibition of NF-κB (Staudt, 2010; Chen et al., 2011; McCurdy and Lacy, 2013).

The conundrum with conventional NF-κB-targeting strategies has been how to achieve cancer cell specificity, given the ubiquitous nature and pleiotropic physiological functions of NF-κB (DiDonato et al., 2012). Because a key pathogenetic activity of NF-κB in MM is to block apoptosis through the induction of target genes, an attractive alternative to globally targeting NF-κB would be to block the nonredundant, cancer-specific downstream effectors of the NF-κB survival function; these effectors, however, are not known. To develop a strategy for inhibiting the NF-κB pathway in a cancer-selective manner and, thus, exploiting its therapeutic potential, we therefore sought to delineate the mechanism(s) underlying the pathological survival activity of constitutive NF-κB signaling in MM. Further, we sought to develop a pharmacological inhibitor of this mechanism(s) in order to kill MM cells effectively and without toxicity to normal cells.

RESULTS

GADD45B Expression Denotes More Aggressive Disease in MM

Given their key role in oncogenesis, we sought to investigate the downstream mechanisms mediating NF-κB survival signaling in MM. Because this signaling involves the induction of antiapoptotic NF-κB target genes, and we had previously identified the *GADD45*-family gene, *GADD45B*, as a transcriptional target of NF-κB encoding a potent and selective inhibitor of the JNK MAPK pathway and, therefore, of apoptosis (De Smaele et al., 2001; Papa et al., 2004), we investigated the involvement of this gene in MM. *GADD45B* was markedly upregulated in monoclonal CD138$^+$ PCs from MM patients compared with monoclonal PCs from patients with monoclonal gammopathy of undetermined significance (MGUS), a premalignant condition (Kyle and Rajkumar, 2009), or healthy polyclonal PCs (Figure 1A), thus establishing a correlation between *GADD45B* mRNA expression and PC malignancy. Strikingly, when MM patients were stratified at diagnosis on the basis of the *GADD45B* mRNA expression in CD138$^+$ cells, the cohort of patients expressing high levels of *GADD45B* exhibited dramatically shorter progression-free survival and significantly shorter overall survival (OS) than the cohort of patients expressing low levels of *GADD45B*, despite both groups of patients having been treated with the same velcade/melphalan/prednisone protocol (Palumbo et al., 2010) (Figures 1B and 1C). A similar correlation of *GADD45B* expression with poor clinical outcome was observed using two independent gene expression data sets of newly diagnosed MM patients, thus providing external validation of our findings (Broyl et al., 2010; Dickens et al., 2010) (Figures S1A and S1B available online). Collectively, these results establish a strong correlation between *GADD45B* expression and disease progression in MM and identify GADD45β as a hallmark of more aggressive disease.

Constitutive NF-κB Activity Promotes the Survival of MM Cells by Inhibiting JNK Signaling

GADD45β inhibits apoptosis by suppressing JNK signaling. It mediates this function by binding to the JNK kinase MKK7 and blocking its enzymatic activity by engaging the kinase catalytic pocket (Papa et al., 2004, 2007). This activity of GADD45β on the JNK pathway and the elevated *GADD45B* mRNA levels observed in monoclonal PCs from MM patients (Figure 1A) prompted us to investigate whether GADD45β mediated an antiapoptotic crosstalk between the NF-κB and JNK pathways in MM cells. We reasoned that cell extrinsic stimulation, as well as intrinsic oncogenic signals and NF-κB-pathway mutations, could result in the activation of signaling pathways beyond the IKK/NF-κB cascade, such as the JNK pathway (Davies and Tournier, 2012), which we and others have previously shown can trigger apoptosis in a manner that can be suppressed by

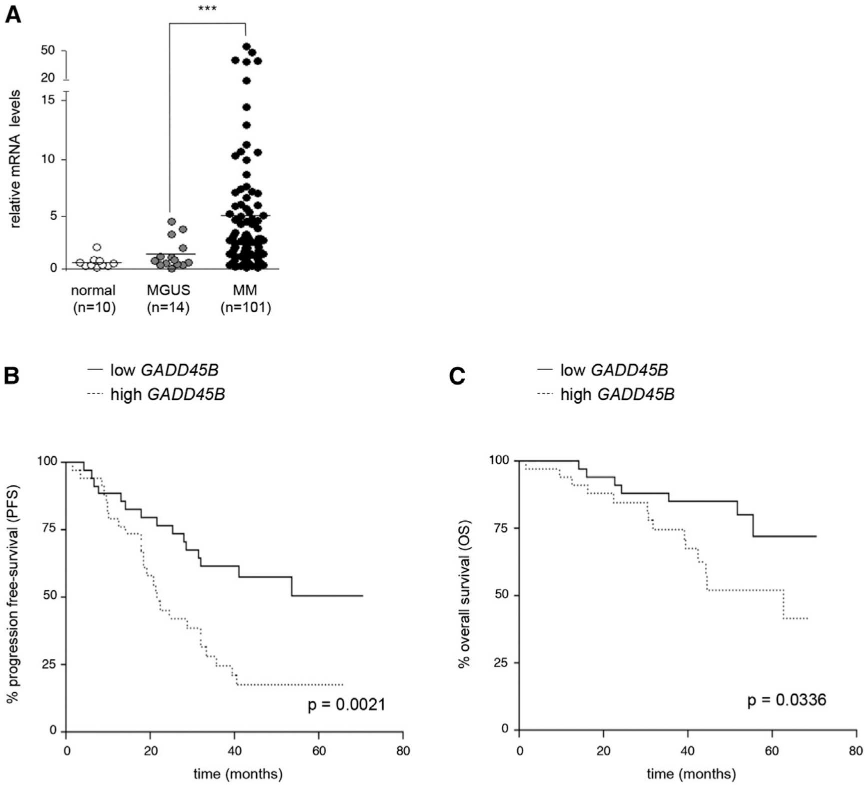

Figure 1. *GADD45B* Is Highly Expressed in MM Cells and Associates with Aggressive Disease

(A) qRT-PCR showing the *GADD45B* mRNA levels in monoclonal CD138$^+$ cells from MM or MGUS patients and polyclonal CD138$^+$ cells (normal). ***p = 0.00018.

(B and C) Progression-free survival (B) and OS (C), as defined by the International Myeloma Working Group Uniform Response Criteria (Kyle and Rajkumar, 2009), in MM patients with low or high *GADD45B* levels, as assessed by qRT-PCR, in CD138$^+$ cells. Patients were from the velcade/melphalan/prednisone arm of the trial and stratified at diagnosis, using as a cutoff the median *GADD45B* expression value.

See also Figure S1.

NF-κB (De Smaele et al., 2001; Tang et al., 2001; Papa et al., 2004). Indeed, at least in principle, this mechanism could provide a basis for the addiction of MM cells to NF-κB for survival. In line with this hypothesis, in each of five heterogeneous MM cell lines and a B lymphoblastoid cell line, the silencing of the NF-κB subunit RelA (DiDonato et al., 2012) triggered JNK activation and apoptosis (Figures S2A–S2C), and blocking this activation with the JNK inhibitor SP600125 effectively protected NF-κB/RelA-silenced MM cells from cell death (Figures S2D and S2E). Similarly, the small hairpin RNA (shRNA)-mediated silencing of MKK7 rescued MM cells from the cytotoxic effects of each of two pharmacological inhibitors of IKKβ/NF-κB, and the protective effect of this silencing was virtually complete (Figure S2F). Collectively, these data indicate that constitutive NF-κB activity promotes the survival of MM cells by inhibiting MKK7/JNK signaling.

GADD45β as a Pivotal Survival Factor Downstream of NF-κB and a Potential Therapeutic Target in MM

To investigate the possible contribution of GADD45β to MM pathogenesis, we examined whether GADD45β mediated the NF-κB-dependent survival function and inhibition of JNK signaling in MM cells. As with PCs from patients (Figure 1A), *GADD45B* was expressed at high levels in MM cell lines compared with most other cancer cell lines tested (Figure S2G, discussed below). Importantly, this high *GADD45B* expression in MM cells markedly diminished upon the silencing of RelA, thus demonstrating its dependence on constitutive NF-κB activity (Figure 2A; Figure S2C). Moreover, similar to the effects of RelA-targeting hairpins (Figures S2A–S2C), the introduction of GADD45β-specific shRNAs, but not of MKK7-specific or of nonspecific shRNAs, induced potent JNK activation and apoptosis in all but two of the MM cell lines tested, namely, the RPMI-8226 and KMM-1 cell lines, which exhibited almost undetectable levels of GADD45β and significantly lower levels of MKK7 than those GADD45β-dependent MM cell lines (Figures 2B and 2C; Figures S2G–S2K, further discussed below). Similar results were observed using additional GADD45β-targeting hairpins, thus confirming the gene-silencing efficiency and specificity of the shRNAs used (Figures S2H, S2J, S2L, and S2M). Strikingly, the extent of JNK activation induced by the silencing of GADD45β in sensitive MM cell lines was similar to that observed with 12-O-tetradecanoylphorbol-13-acetate (TPA)/ionomycin stimulation, which potently induces JNK (Figure 2C; Figures S2I and S2J). By contrast, GADD45β downregulation had no effect on IKK/NF-κB, ERK, or p38 activity. As seen with the inhibition of IKKβ/NF-κB (Figures S2D and S2E), both the silencing of JNK1 and the treatment with SP600125 effectively reversed apoptosis in GADD45β-silenced MM cells (Figures 2D and 2E; Figures S2N–S2P). Hence, GADD45β promotes the survival of MM cells by inhibiting JNK-mediated apoptosis. Collectively, these findings identify GADD45β as an essential NF-κB-regulated survival factor and selective MKK7/JNK-axis inhibitor and, therefore, as a potential therapeutic target in MM.

Development of D-Tetrapeptide Inhibitors of the GADD45β/MKK7 Complex

Given the essential antiapoptotic role of GADD45β in MM and our previous results showing that GADD45β suppresses JNK signaling and apoptosis by blocking MKK7 via direct physical interaction (De Smaele et al., 2001; Papa et al., 2004, 2007), we aimed to develop selective inhibitors of this protein-protein interaction in order to induce cytotoxic JNK signaling in MM cells. We screened a simplified combinatorial library of 20,736 L-tetrapeptides to select compounds capable of disrupting the GADD45β/MKK7 complex (Figure S3A and Table S1). Iterative deconvolution of this library in ELISA competition assays,

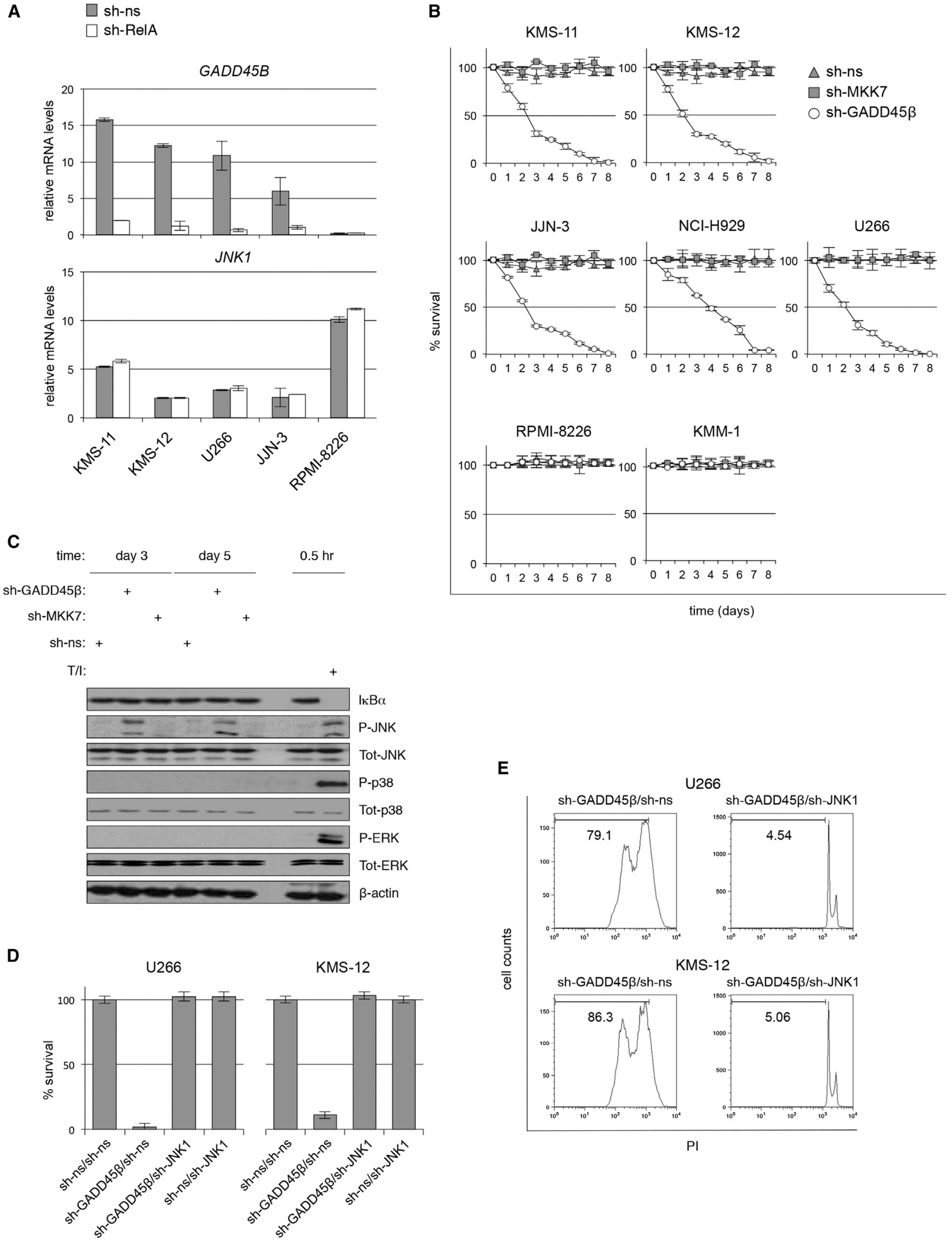

(legend on next page)

followed by secondary screening and optimization of the resulting hits, yielded two *ac*etylated L-*tetrap*eptides of similar structure, namely, Ac-LTP1 and Ac-LTP2, which disrupted the GADD45β/MKK7 complex, in vitro, with remarkable half-maximal inhibitory concentration (IC_{50}) values in the subnanomolar range (Figure 3A; Figure S3B and Table S2), in line with the top-end potencies of other hits isolated from similar peptide library screens (Houghten et al., 1999).

Next, we examined whether these two L-tetrapeptides retained in vitro potency upon synthesis in the D configuration, a strategy used successfully in some cases to render small peptides resistant to proteolysis (Zhou et al., 2002; Nickl et al., 2010). Strikingly, as shown in Figures 3A and 3B, the D-enantiomers of Ac-LTP1 and Ac-LTP2 (termed Ac-DTP1 and Ac-DTP2, respectively) displayed no loss of activity in vitro and, unlike their L counterparts, were highly stable in human serum, even after prolonged incubation. Coimmunoprecipitation assays confirmed the potent and specific inhibitory effect of Ac-DTP1 and Ac-DTP2 on GADD45β/MKK7 complex stability in vitro (Figure 3C). By contrast, control D-tetrapeptides had no such effect on stability, thus demonstrating the specificity of the inhibitory activities of Ac-DTP1 and Ac-DTP2. Crucially, the disruption of the complex by these two active D-peptides completely reversed the GADD45β-mediated inhibition of MKK7, fully restoring the kinase catalytic activity (Figure 3D, top). Importantly, none of Ac-DTP1, Ac-DTP2, or any of the control D-tetrapeptides affected MKK7 activity in the absence of GADD45β (Figure 3D, bottom). Collectively, these findings identify a downstream, drug-targetable module in the NF-κB pathway and confirm the potential of our pharmacological approach for inducing cytotoxic MKK7/JNK signaling in cells that rely on GADD45β for restraining MKK7 activation.

To verify the therapeutic potential of GADD45β/MKK7 inhibitors, we aimed to improve their cell penetration. We replaced the N-terminal acetyl group of Ac-DTP1 and Ac-DTP2 with a benzyloxycarbonyl [Z] group, thereby generating z-DTP1 and z-DTP2, respectively (Table S3), which retained high activity and stability in vitro (Figures S4A and S4B) and, importantly, also exhibited potent cytotoxic activity across a panel of genetically heterogeneous MM cell lines (Figures 4A and 4B; Figures S4C–S4F and Table S3). Significantly, z-DTP1 and z-DTP2, but not a control D-tetrapeptide, induced potent and dose-dependent toxicity in all of the MM cell lines tested, except the two expressing nearly undetectable levels of *GADD45B* and low levels of MKK7 (further discussed below), exhibiting IC_{50} values in the sensitive MM cell lines in the low nanomolar to low micromolar range (Figures 4A and 4B; Figures S2G, S4E, and S4F). Consistent with the protective mechanism mediated by GADD45β (Figure S2G), the z-DTP1- and z-DTP2-afforded killing of sensitive MM cells was due to the induction of apoptosis (Figure 4C; Figures S4G and S4H). Importantly, both active D-peptides retained potent and cancer-selective activity in primary PCs from MM patients (Figure 4D, left). Crucially, these D-peptides also exhibited an apparently complete lack of toxicity to normal cells, even when used at very high concentrations (i.e., 100 μM; Figure 4D, right; Figure S4I). Hence, D-tetrapeptide antagonists of the GADD45β/MKK7 complex show exceptionally high activity and cancer cell specificity in terms of apoptosis induction in MM cells, without displaying any apparent toxicity to normal cells.

The Development of DTP3: A GADD45β/MKK7 Inhibitor with Improved Bioavailability

To improve the bioavailability of D-peptides in vivo, while retaining high cellular activity and specificity toward the GADD45β/MKK7 complex, we used a chemical optimization strategy based on structure-activity relationship and pharmacophore analyses (Figures S5A–S5C and Table S4). By combining these methods, we developed DTP3, a D-tripeptide with a molecular weight of 525 Da (Figure 5A), which retained all the main characteristics of the parental D-tetrapeptides in terms of bioactivity and specificity, including subnanomolar activity and high stability in vitro and potent and selective capacity to kill MM cells via apoptosis (Figures S5D–S5H), while exhibiting a superior pharmacokinetic profile compared with the parent molecules (discussed below).

We further evaluated the modality and specificity of the binding of DTP3 to the GADD45β/MKK7 complex. Circular dichroism (CD) studies demonstrated that DTP3, but not a control scrambled (SCRB) D-tripeptide, can physically interact with and cause a significant conformational change of the structure of this complex, as well as of the isolated MKK7 protein, in a dose-dependent manner (Figure S5I). By contrast, DTP3 had no effect on the CD signal of GADD45β (Figure S5I). MALDI-TOF mass spectrometry confirmed the ability of DTP3 to bind to MKK7, but not to GADD45β (Figure S5J). Spectrofluorimetric analyses yielded similar results and, furthermore, established the 1:1 stoichiometry and low equilibrium dissociation constant (K_D) value of the DTP3 interaction with MKK7 (Figure 5B; Figure S5K), thus underscoring the strength and specificity of this interaction. Conversely, the SCRB D-tripeptide had no impact on the fluorescence emission spectrum of MKK7, thus reaffirming the specificity of the effects of DTP3. Computational analyses predicted the presence of a pocket of MKK7, also found on the GADD45β/MKK7 complex, that can bind to DTP3 (Figures S5L–S5T). Collectively, these results demonstrate that

Figure 2. GADD45β Mediates the NF-κB Survival Activity in MM Cells by Suppressing JNK Signaling

(A) qRT-PCR showing the *GADD45B* mRNA levels in MM cell lines expressing nonspecific (sh-ns) or RelA-specific (sh-RelA) shRNAs. *JNK1* is shown as control. Values denote mean ± SD (n = 3).

(B) Survival of MM cell lines expressing sh-ns, GADD45β-specific (sh-GADD45β), or MKK7-specific (sh-MKK7) shRNAs. Values express the percentage of live $eGFP^+$ cells relative to the number of live $eGFP^+$ cells in the same culture on day 0. Values denote mean ± SD (n = 3).

(C) Western blots showing total and phosphorylated (P) proteins in U266 MM cells from (B). T/I, TPA/ionomycin.

(D) Trypan blue exclusion showing the survival of representative MM cell lines coexpressing sh-GADD45β or sh-ns and JNK1-specific (sh-JNK1) or sh-ns shRNAs on day 8 after lentivirus infection. Values denote mean ± SD (n = 3).

(E) PI staining showing apoptotic cells (i.e., cells with sub-G_1 DNA content) in representative MM cell lines from (D). The percentages of apoptotic cells are depicted.

See also Figure S2.

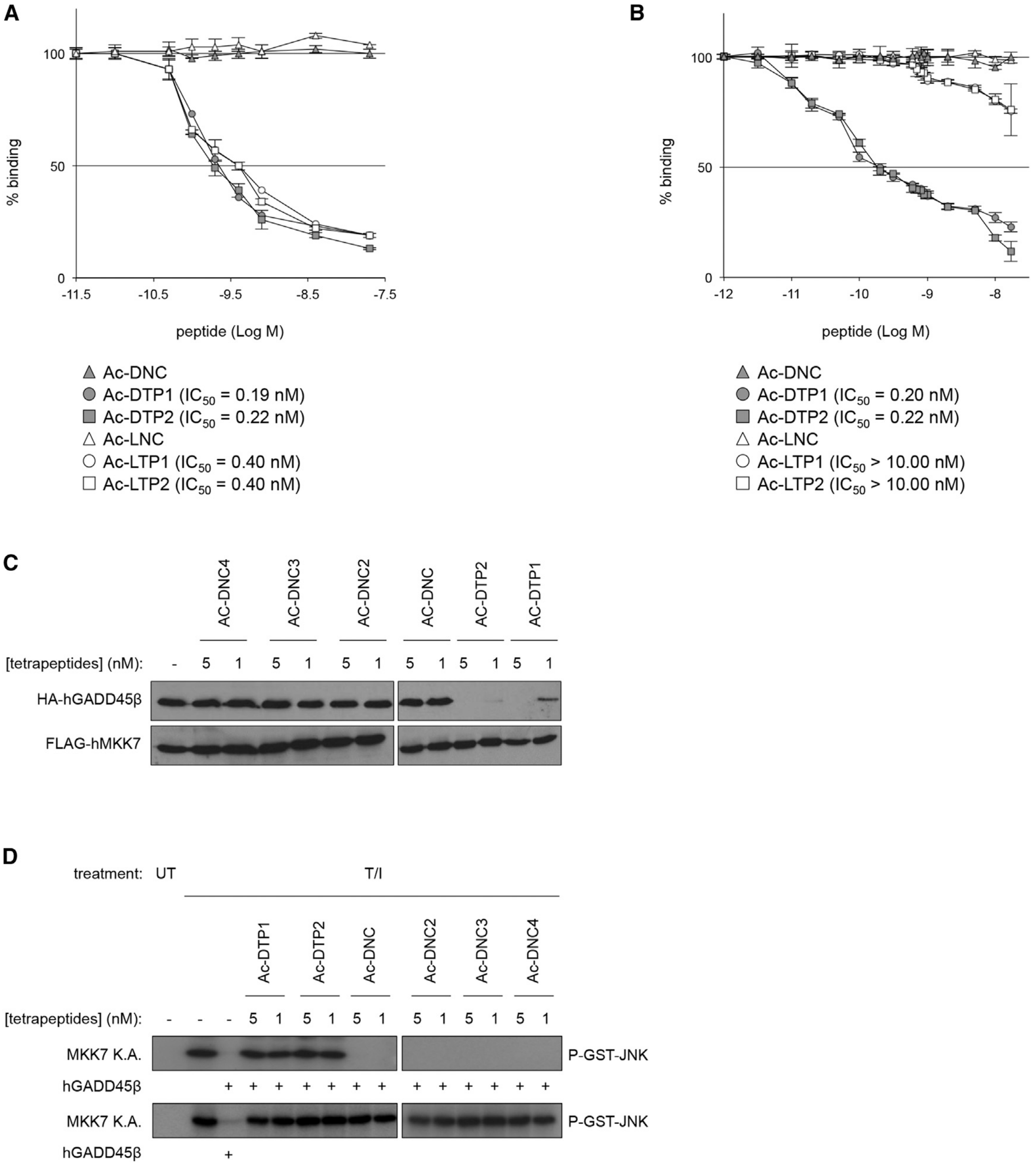

Figure 3. The Potent Activity and Stability of D-Peptide Inhibitors of GADD45β/MKK7 In Vitro

(A and B) ELISA GADD45β/MKK7 competition assays showing the IC_{50} values of the active L-tetrapeptides (Ac-LTP1, Ac-LTP2) and D-tetrapeptides (Ac-DTP1, Ac-DTP2), before (A) and after (B) a 48 hr preincubation with human serum. Values express the percentage of inhibition of GADD45β binding to MKK7 relative to the binding in the absence of peptide and denote means ± SD (n = 3). Ac-LNC, acetylated (Ac) negative control L-tetrapeptide (LNC); Ac-DNC, Ac negative control D-tetrapeptide (DNC).

(C) Coimmunoprecipitations (IP) performed with cell lysates prepared from human embryonic kidney 293T cells expressing ectopic HA-tagged GADD45β (HA-hGADD45β) and FLAG-tagged MKK7 (FLAG-hMKK7) and incubated with anti-FLAG antibody in the presence or absence of bioactive (Ac-DTP1 and Ac-DTP2) or inactive (Ac-DNC, Ac-DNC2, Ac-DNC3 and Ac-DNC4) D-tetrapeptides, as indicated. Western blots were developed using anti-HA or anti-MKK7 antibodies. -, incubation without D-tetrapeptides.

(D) Kinase assays (K.A.) showing MKK7 activity before (-) and after incubation with Ac-DTP1, Ac-DTP2 or control D-tetrapeptides as in (C), in the presence (+) or absence of recombinant human (h)GADD45β. T/I, TPA/ionomycin; UT, untreated.

See also Figure S3 and Tables S1 and S2.

DTP3 has the capacity to physically interact with MKK7, both in isolation and within the complex with GADD45β, and support a model whereby, upon binding to MKK7, DTP3 dissociates the GADD45β/MKK7 complex via an allosteric mechanism, potentially involving a conformational rearrangement of the kinase.

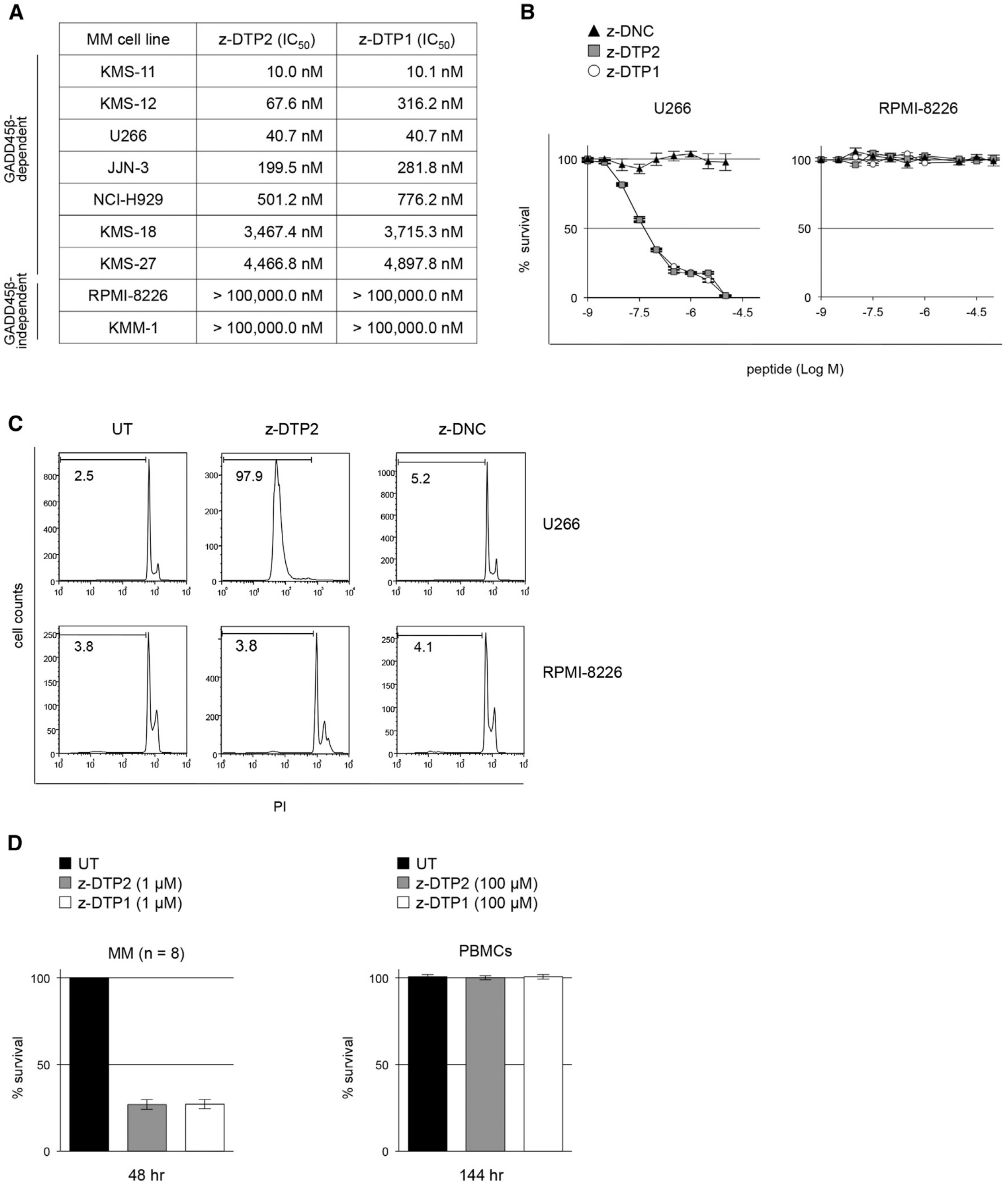

Figure 4. The Potent and Cancer-Selective Activity of D-Peptide Inhibitors of GADD45β/MKK7 in MM Cells

(A) IC_{50} values of z-DTP1 and z-DTP2 at 144 hr, as determined by [^{3}H]thymidine incorporation, in MM cell lines that depend or do not depend on GADD45β for survival.

(B) [^{3}H]Thymidine incorporation showing the survival of representative sensitive (U266) and resistant (RPMI-8226) MM cell lines from (A) after a 6-day treatment with the indicated concentrations of z-DTP1, z-DTP2, or Z-protected (z)-DNC.

(C) PI staining showing apoptotic cells in representative MM cell lines from (B), after treatment with 10 μM of z-DTP1, z-DTP2 or z-DNC for 6 days. The percentages of apoptotic cells are indicated.

(D) Trypan blue exclusion showing the survival of CD138$^+$ cells from MM patients (n = 8) and healthy human PBMCs after treatment with z-DTP1 or z-DTP2 for 48 and 144 hr, respectively.

(B and D) Values express the percentage of live cells present in the treated cultures relative to the live cells present in the respective untreated cultures, represented as 100%, and denote means ± SD (B), or SEM (D) [PBMCs] (n = 3), (D) [MM] (n = 8).

See also Figure S4 and Table S3.

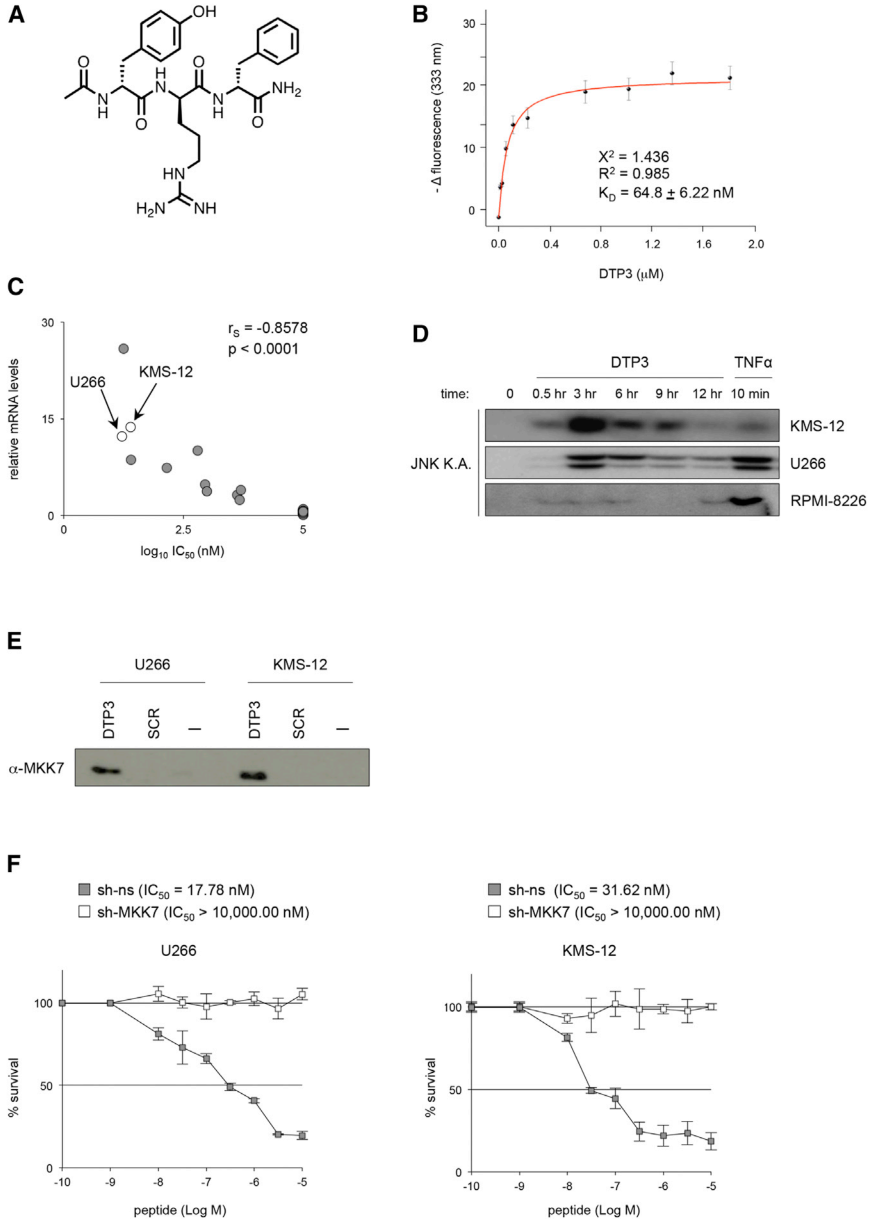

Figure 5. The High Target Specificity of DTP3 in Cells

(A) The structure of DTP3.

(B) Tryptophan fluorescence quenching analysis showing the dose-response curve of the -Δfluorescence values of GST-hMKK7 at 333 nm plotted against the concentration values of DTP3. The stoichiometry and K_D value of the DTP3/MKK7 interaction on the basis of these data were 1:1 and 64.81 ± 6.22 nM, respectively. Values denote means ± SD (n = 3).

(C) Correlation plot of the relative *GADD45B* mRNA levels (qRT-PCR) and the DTP3 IC_{50} at 144 hr ([^{3}H]thymidine incorporation) in cancer cell lines of different tissues of origin. Values on the x axis express the logarithm to base 10 (log_{10}) of the IC_{50}. r_S, Spearman correlation coefficient.

(D) K.A. showing JNK activity in representative sensitive (KMS-12, U266) and resistant (RPMI-8226) MM cell lines after treatment with DTP3 (10 μM). TNFα is shown as a positive control.

(E) Peptide pull-down showing the physical association of DTP3 with endogenous MKK7 in U266 and KMS-12 MM cells. SCRB D-peptide is used as a negative control. -, pull-down without D-tripeptide.

(F) [^{3}H]Thymidine incorporation showing the survival of U266 and KMS-12 MM cells expressing sh-ns or sh-MKK7 shRNAs after a 6-day treatment with the indicated concentrations of DTP3. The IC_{50} values of DTP3 are depicted. Values express the percentage of the counts per minute (cpm) measured with the treated cultures relative to the cpm measured with the respective untreated cultures and denote mean ± SD (n = 3).

See also Figure S5 and Tables S4 and S5.

The High Target Specificity of DTP3 for the GADD45β/MKK7 Complex

In a panel of tumor cell lines of different tissues of origin, the sensitivity to DTP3-induced killing correlated with a very high degree of significance with the mRNA levels of *GADD45B* (Figure 5C; Figures S2G and S5U), which our data have shown is a pivotal NF-κB-regulated inhibitor of MKK7/JNK signaling and apoptosis in MM cells (Figures 2B–2E; Figures S2H–S2J and S2N–S2P). Significantly, DTP3 displayed potent and selective activity in both MM and non-MM cell lines exhibiting high levels of *GADD45B* expression, whereas it was completely inactive, even at high micromolar concentrations, in tumor cell lines featuring low *GADD45B* expression (Figures S2G and S5F–S5H). In the context of MM, the RPMI-8226 and KMM-1 cell lines, which express very low levels of GADD45β and low levels of MKK7 and are consequently unaffected by GADD45β-targeting hairpins, were completely refractory to DTP3-induced killing (Figure 2B; Figures S2G–S2I, S2K, and S5F–S5H).

As seen with GADD45β-silencing shRNAs (Figure 2C; Figures S2H and S2I), treatment with DTP3 effectively activated JNK but not p38, ERK, or IKK/NF-κB signaling in sensitive, GADD45β-dependent MM cell lines, whereas it did not affect any of these pathways in DTP3-resistant, GADD45β-independent MM cell lines, such as RPMI-8226 (Figure 5D; Figure S5V; see also Figure S4H, z-DTP2). Strikingly, the magnitude of the effects of DTP3 on the JNK pathway was similar to that observed with tumor necrosis factor α (TNFα), a potent inducer of JNK (Figure 5D; Figures S4H and S5V, TPA/ionomycin). By contrast, a negative control D-peptide had no effect on JNK signaling (Figure S5V). As expected from the in vitro data (Figure 5B; Figure S5I, middle, bottom; Figure S5J, left), these activating effects of DTP3 on the JNK pathway hinged upon the ability of this D-tripeptide to bind to MKK7 in cells, as shown by DTP3 pull-down assays (Figure 5E). These effects were not observed with the SCRB control peptide, nor were they observed with a pull-down control in the absence of peptide, thus demonstrating the specificity of the

binding of DTP3 to endogenous MKK7. Importantly, knocking down the GADD45β target kinase, MKK7, or its downstream effector, JNK1, completely abolished the cytotoxic activity of DTP3 in sensitive MM cell lines (Figure 5F; Figures S5W and S5X), thus excluding any off-target toxicity of DTP3 in cells. DTP3 also lacked any off-target effect when profiled in kinase assays against a panel of 142 human kinases (Table S5). Hence, bioactive D-peptides selectively induce apoptosis in MM cells with functional MKK7 and elevated GADD45β expression by activating JNK signaling via MKK7. These findings also correlate the pharmacological activity of DTP3 with a tumor cell dependence on GADD45β for survival and establish the exceptionally high target specificity of this D-tripeptide for the GADD45β/MKK7 complex in cells.

The Potent and Cancer-Selective Activity of DTP3 in MM Cells from Patients

To verify the suitability of DTP3 for the treatment of human diseases, we tested its activity and selectivity in MM PCs from patients. As seen with the active D-tetrapeptides (Figure 4D, left), DTP3 effectively killed primary MM PCs at low nanomolar concentrations (Figures 6A and 6B). Importantly, it retained potent and selective cytotoxic activity in these cells upon stimulation with interleukin-6 or insulin growth factor 1 or coculture with bone marrow stromal cells (BMSCs) (Figure 6C; Figures S6A and S6B), which promote the survival of MM cells (Hideshima et al., 2005). In order to compare the efficacy and cancer cell specificity of DTP3 with the corresponding parameters of bortezomib, the current gold-standard treatment for MM (Chen et al., 2011), we defined an “in vitro therapeutic index” (Figure 6A, bottom). As shown in Figure 6A (top), DTP3 had a similar IC_{50} value to bortezomib in primary MM PCs but, importantly, unlike bortezomib, which barely discriminated between malignant and normal cells, had no toxicity to normal cells. Strikingly, because of this cancer cell selective target specificity, DTP3 had an in vitro therapeutic index that was greater, by more than two orders of magnitude, than that of bortezomib (Figure 6A, bottom). DTP3, in fact, could also distinguish between PCs from MM and Waldenström’s macroglobulinemia (WM) patients (Kyle and Rajkumar, 2009), in line with the *GADD45B* expression levels in these cells, whereas bortezomib could not (Figures 6A and 6B; Figure S6C). Similarly, DTP3 displayed far more potent activity in primary MM PCs and far less toxicity to normal cells than the IKKβ inhibitor, PS-1145 (Figures 6C and 6D; Figures S6A and S6B; note the different concentrations of DTP3 and PS-1145 used). Because the standard MM treatment consists of combination therapy, and nearly all patients will relapse and/or develop drug resistance at some point, we evaluated the potential of DTP3 to operate in these settings. As shown in Figures S6D and S6E, DTP3 displayed synergistic activity with bortezomib in two different MM cell lines, exhibiting a combination index of 0.21 in U266 cells and of 0.56 in KMS-12 cells, suggesting that it could find indication in the clinic in combination with bortezomib (Chou, 2006). Importantly, DTP3 also retained full therapeutic efficacy in MM cell lines that were resistant to conventional MM treatments, such as dexamethasone, bortezomib, and lenalidomide (Bjorklund et al., 2011, 2014, Rückrich et al., 2009) (Figures S6F and S6G). Together, these results provide compelling evidence of the high therapeutic potential of DTP3 in MM patients.

The Potent Therapeutic Efficacy and Excellent Tolerability of DTP3 against MM In Vivo

DTP3 showed high aqueous solubility and very high stability in human serum, owing to its resistance to proteolysis, with a good pharmacokinetic profile and excellent in vivo tolerability, suitable for a therapeutic purpose (Figure 7A; Figure S5D and Table S6).

Remarkably, in a plasmacytoma model, treatment with DTP3 at the dose of 14.5 mg/kg/day virtually eradicated established subcutaneous myeloma xenografts in mice, in the absence of any apparent side effects (Figures 7B and 7C; Figure S7A and Table S6). Similar results were obtained in a second plasmacytoma model, generated using a different MM cell line (Figures S7C and S7D). At the experimental end point, on day 28, all the control mice had developed large local tumors, whereas all the mice in the DTP3-treated cohort had shown a dramatic shrinkage of the tumors (Figures 7B and 7C; Figures S7C and S7D). This therapeutic effect of DTP3 was due to the potent and tumor-selective induction of JNK activation and apoptosis (Figures 7D and 7E; Figure S7B), as shown by the appearance of phosphorylated JNK, as early as 24 hr of the onset of treatment with DTP3, but not with PBS, followed by the appearance, starting on day 3, of caspase-3 and PARP-1 proteolysis products. Coincident with these events, apoptotic cells became evident in the tumor tissue at day 3 (Figure 7D; Figure S7B). As well as the tumor-ablative effects of DTP3 (Figure 7B; Figure S7A), the extent of this JNK-associated, tumor cell apoptosis markedly increased in magnitude over time (Figure 7D; Figure S7B).

Importantly, DTP3 retained potent anticancer activity in an orthotopic xenograft model of MM, which more faithfully recapitulates the human disease. All the control mice developed severe limb paralysis and died within 32 days of treatment start, resulting in a median OS of 26 days (Figure 7F). Strikingly, DTP3 administration over a period of 8 weeks, at a dose of 29 mg/kg/day, extended the median OS of the mice past the experimental end point on day 161, without producing any apparent side effect, thus demonstrating the potent therapeutic efficacy of DTP3 against MM, in vivo, and the excellent tolerability of this agent at doses that achieve full therapeutic efficacy. Collectively, together with the data in primary MM cells (Figures 6A–6D; Figures S6A and S6B), these results underscore the potency, safety, and cancer cell specificity of the pharmacological approach targeting the GADD45β/MKK7 complex in MM and identify DTP3 as a therapeutic selectively inhibiting the NF-κB survival pathway in cancer (Figure 8).

DISCUSSION

We identified the GADD45β/MKK7 complex as a functionally critical survival module downstream of NF-κB and a therapeutic target in MM. Further, we developed a corresponding D-tripeptide inhibitor of this complex, DTP3, which effectively kills MM cells by inducing MKK7/JNK-dependent apoptosis and, at the same time, does not appear to be toxic to normal tissues. Because of this cancer cell specificity, DTP3 displays an exceptionally high therapeutic index in vitro and potent and cancer-selective activity against MM in vivo. Future studies will clarify the precise mechanism by which DTP3 dissociates the

A

	cell type	DTP3 (IC_{50})	Bortezomib (IC_{50})
MM	PCs (n = 8)	28.2 ± 0.1 nM	8.7 ± 2.1 nM
WM	PCs (n= 2)	> 10,000.0 nM	3.38 ± 2.0 nM
normal	PBMCs	> 100,000.0 nM	63.1 nM
	BMSCs	> 100,000.0 nM	80.0 nM
	MCSs	> 100,000.0 nM	79.4 nM
	splenocytes	> 100,000.0 nM	80.0 nM
	lymph node cells	> 100,000.0 nM	91.1 nM
in vitro therapeutic index (IC_{50} in BMSCs/IC_{50} in MM cells)		> 3,542.3	9.2

B

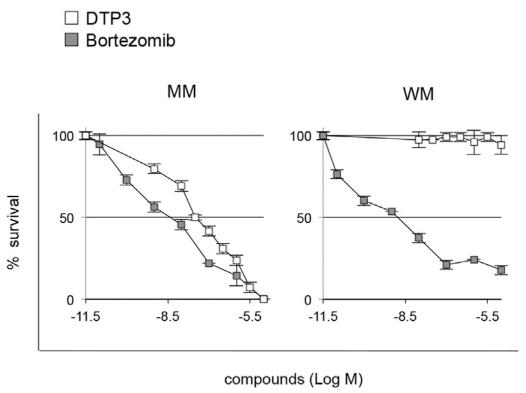

C

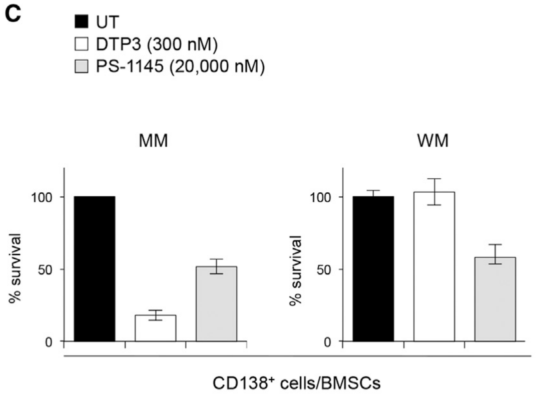

D

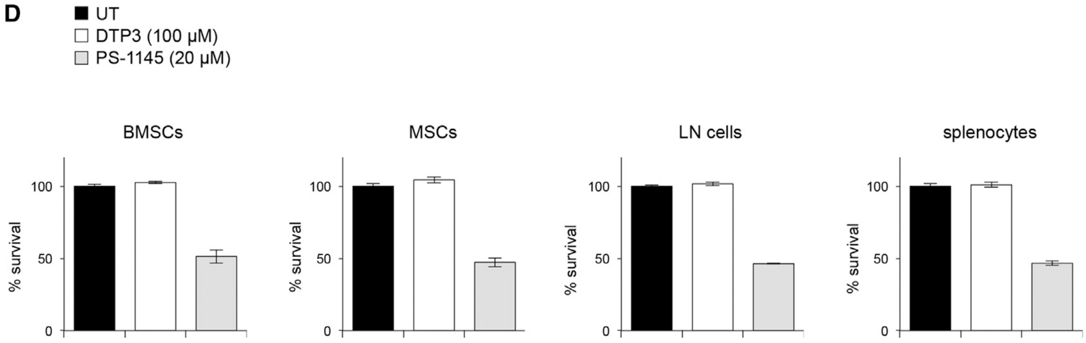

Figure 6. The Potent Activity of DTP3 in Primary MM Cells and Its Far Superior Cancer Cell Selectivity Compared with IKKβ and Proteasome Inhibitors

(A) IC_{50} values of DTP3 and bortezomib at 48 hr in $CD138^+$ cells from MM patients (n = 8), $CD138^+$ cells from WM patients (n = 2), human PBMCs, human BMSCs, human mesenchymal stem cells (MSCs), and mouse splenocytes and lymph node (LN) cells (top), as determined by trypan blue exclusion. In vitro therapeutic indices (IC_{50} in BMSCs/IC_{50} in MM PCs) are depicted (bottom). MM and WM PC values denote means ± SEM. Other values denote means (n = 3).
(B) Trypan blue exclusion showing the survival of $CD138^+$ cells from representative patients from (A), after treatment with DTP3 or bortezomib for 48 hr.
(C) Trypan blue exclusion showing the survival of $CD138^+$ cells from MM or WM patients after treatment with DTP3 (300 nM) or PS-1145 (20 μM) for 48 hr, in the presence of BMSCs.
(D) Trypan blue exclusion showing the survival of primary human BMSCs, human MSCs, and mouse LN cells and splenocytes after treatment with DTP3 (100 μM) or PS-1145 (20 μM) for 144 hr.
(B–D) Values express the percentage of live cells present in the treated cultures relative to the live cells present in the respective untreated cultures and denote means ± SD (B), (C) [WM], (D) (n = 3) or SEM (C) [MM] (n = 9).
See also Figure S6.

A

in vivo PK	DTP3
$t_{1/2}$	1.26 h ± 0.75 hr
CL	27.13 ± 4.09 mL/min/kg
V_d	2.80 ± 1.26 L/kg
AUC	6.43 ± 0.01 µg·hr/mL

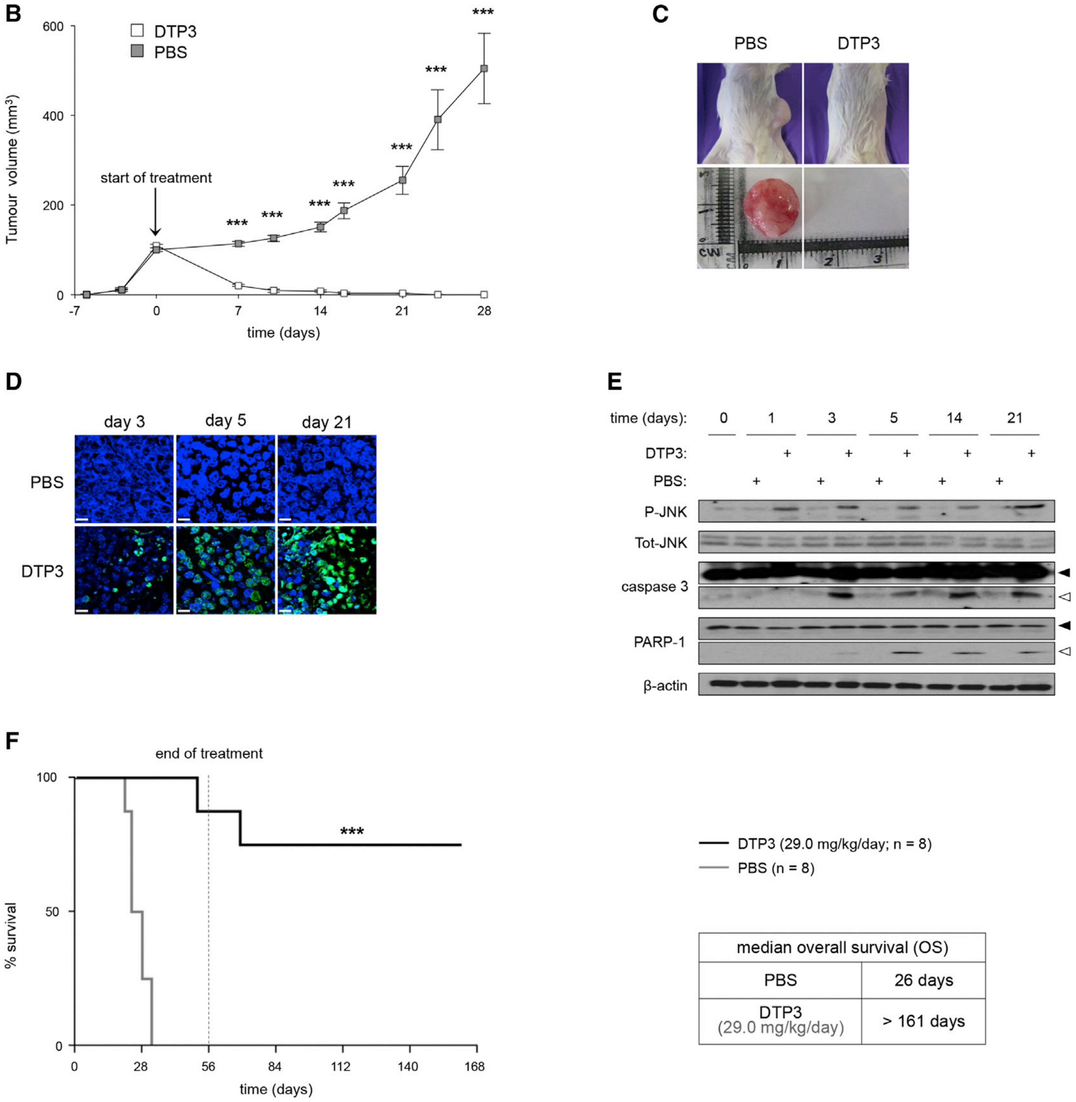

median overall survival (OS)	
PBS	26 days
DTP3 (29.0 mg/kg/day)	> 161 days

Figure 7. DTP3 Exhibits Potent Therapeutic Activity against MM, In Vivo, in the Absence of Any Apparent Side Effects

(A) Pharmacokinetic (PK) values of DTP3 after single intravenous injection at the dose of 10 mg/kg. AUC, area under the plasma concentration versus time curve; CL, plasma clearance; $t_{1/2}$, terminal half-life; V_d, volume of distribution. Values denote means ± SD (n = 3).

(B) Volumes of subcutaneous U266 myeloma xenografts in mice treated by continual infusion with DTP3 at a dose of 14.5 mg/kg/day or PBS for the times shown. Values denote means ± SEM (n = 16). ***$p < 0.001$.

(C) Images of representative myeloma-bearing mice (top) and isolated tumors (bottom) from (B) at day 28.

(D) Images of TUNEL assays showing apoptotic cells in representative tumors from (B). Scale bars represent 10 µM. Green, TUNEL; blue, DAPI.

(E) Western blots showing total and phosphorylated (P) JNK, and the unprocessed (filled arrowheads) and cleaved (open arrowheads) forms of caspase-3 and its proteolytic substrate, PARP-1, in representative tumors from (B).

(F) Percentage survival of mice bearing medullary KMS-12 MM xenografts and treated intermittently by infusion with DTP3 at a dose of 29.0 mg/kg/day or PBS (left; n = 8, each group) for 8 weeks. Also shown is the median OS of each animal cohort (right). ***$p < 0.0001$.

See also Figure S7 and Table S6.

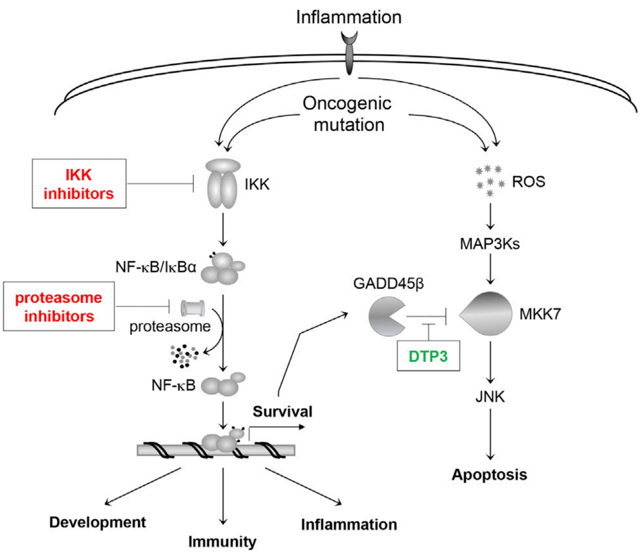

Figure 8. Schematic Representation of the Therapeutic GADD45β/MKK7-Targeting Strategy in Cancer

Depicted is the crosstalk between the NF-κB and JNK pathways promoting the survival of cancer cells and cells exposed to inflammatory stimuli. Also illustrated is the NF-κB-dependent, Gadd45β/MKK7-mediated mechanism suppressing apoptotic JNK signaling in MM cells. Our therapeutic strategy to block the NF-κB survival function in a cancer-selective manner with DTP3 (green) is compared with conventional therapeutic approaches (e.g., IKKβ and proteasome inhibitors; red) also aimed at inhibiting the NF-κB pathway in cancer. MAP3Ks, mitogen-activated protein kinase kinase kinases; ROS, reactive oxygen species.

GADD45β/MKK7 complex. Notwithstanding, our findings uncover a mechanism for the pathogenetic survival activity of NF-κB in MM. Crucially, they also demonstrate that cancer-selective inhibition of the NF-κB survival pathway is possible and provide a promising therapy with no preclusive toxicity that could be of profound benefit for patients with this cancer and, potentially, others for which NF-κB promotes survival via GADD45β (Lam et al., 2005; Ngo et al., 2006; Tracey et al., 2005).

The therapeutic targeting of the NF-κB pathway has been aggressively pursued for the treatment of a wide range of inflammatory and malignant pathologies, including MM (DiDonato et al., 2012). However, it has proved so far an insurmountable challenge. Current therapeutic approaches, such as IKKβ inhibitors, target core components of this pathway, and so, although they are potentially capable of abrogating the cancer-promoting activities of NF-κB, they fail to preserve its pleiotropic physiological functions, such as functions in immunity and inflammation (Staudt, 2010; DiDonato et al., 2012). Because the best documented function of NF-κB in cancer is to induce genes that block apoptosis and, despite its ubiquitous nature, NF-κB signaling elicits highly tissue- and context-specific transcriptional programs (DiDonato et al., 2012), we sought to develop a therapeutic approach capable of inhibiting the NF-κB antiapoptotic activity, in a cancer-selective manner. We reasoned that agents targeting a nonredundant, downstream module within this critical survival axis of the NF-κB pathway and having functional restriction to the cancer cells (e.g., the GADD45β/MKK7 module in the case of MM) would provide a more selective and, therefore, more effective therapy, lacking the dose-limiting toxicities of conventional drugs globally targeting NF-κB. It is noteworthy in this regard that most normal cells do not express *GADD45B* constitutively (Zhang et al., 2005). Furthermore, unlike mice lacking RelA or any subunit of the IKK complex (DiDonato et al., 2012), *Gadd45b*$^{-/-}$ mice are viable, are fertile, and die of old age (Papa et al., 2008; Lu et al., 2004), indicating that, in contrast to global NF-κB blockade, complete GADD45β inactivation is well tolerated in vivo.

Our approach also aims at exploiting the general good safety of peptide therapeutics and the greater selectivity afforded by inhibiting a protein-protein interaction, rather than the entire function of a receptor or an enzyme, as is the case for most drugs. Because of this property and their unique mode of action, it is expected that the side effects of GADD45β/MKK7 inhibitors in patients will be milder than the phenotypes of *Gadd45b*$^{-/-}$ mice (Papa et al., 2008; Lu et al., 2004), both because of the transient nature of chemotherapeutic treatments and because these agents would enable GADD45β to retain its MKK7-independent functions. The partially impaired T_H1 T cell response reported in *Gadd45b*$^{-/-}$ mice (Lu et al., 2004), for example, was shown to depend on the Gadd45β-afforded regulation of the MAP3K, MEKK4 (Chi et al., 2004), but not of MKK7. Similarly, such agents would enable MKK7 to retain its enzymatic function and GADD45β-independent modalities of regulation. Nevertheless, future studies will be required to address the potential side effects of DTP3 in patients.

Many cancers beyond MM rely on constitutive NF-κB signaling for survival (Staudt, 2010; DiDonato et al., 2012). Together, the high expression of *GADD45B* in a subset of these cancers, including diffuse large B cell lymphoma (DLBCL) and other types of lymphoma (Lam et al., 2005; Ngo et al., 2006; Tracey et al., 2005), and the selective toxicity of DTP3 in DLBCL, Burkitt's lymphoma and promonocytic leukemia cell lines, imply that GADD45β/MKK7 antagonists may have broader therapeutic potential beyond MM, in other areas of unmet need within oncology. By contrast, certain MM cell lines express low levels of GADD45β, as well as of MKK7, and are refractory to DTP3-induced killing. This suggests the existence of GADD45β-independent mechanisms for NF-κB-dependent survival in certain subtypes of MM and, most likely, in other types of malignancy. For instance, BCL-2 family members are possible mediators of such mechanisms, at least in the context of MM (Gomez-Bougie and Amiot, 2013). Therefore, on the basis of the principle we describe here of targeting an axis of the NF-κB pathway with cancer-restricted function, rather than NF-κB globally, delineating these mechanisms could provide new strategies for targeting NF-κB in a disease-specific manner also in GADD45-β-independent, NF-κB-addicted malignancies and, perhaps, nonmalignant NF-κB-driven pathologies.

EXPERIMENTAL PROCEDURES

Cell Purification and Culture

Cells were cultured according to standard protocols (Mauro et al., 2011; Piva et al., 2008). BMSCs and peripheral blood mononuclear cells (PBMCs) were purified from MM patients or the blood of healthy volunteers, respectively, as reported in Piva et al. (2008). CD138$^+$ cells were purified from the bone marrow aspirates of patients using CD138 MicroBeads (Miltenyi Biotech). Patients were recruited at the Hematology Division of "Ospedale San Giovanni

Battista" (Turin, Italy) and the Haematology Clinic at Imperial College Healthcare NHS Trust, under the approval of the Ethics Committee of "Ospedale San Giovanni Battista" (VMP-VMPT trial 163) and the London Harrow Research Ethics Committee (11/LO/1628), respectively. Written consent was documented for all subjects. Additional details are provided in Supplemental Experimental Procedures.

Biochemical Assays
Quantitative RT-PCR (qRT-PCR) assays were carried out using the TaqMan Gene Expression Assays kit (Applied Biosystems). The kinase profiling of DTP3 across 142 human kinases was outsourced. Western blots, coimmunoprecipitations, and kinase assays were performed as described previously (Papa et al., 2004, 2008; Mauro et al., 2011). Additional details are provided in Supplemental Experimental Procedures.

Cellular Assays
Lentiviral infections were performed using pLentiLox.3.7, as described by Mauro et al. (2011). Enhanced GFP (eGFP)$^+$ cells were purified, when necessary, by fluorescence-activated cell sorting. [^{3}H]Thymidine incorporation and trypan blue exclusion assays were performed using standard methods (Mauro et al., 2011). IC_{50} values were defined as the mean concentration of compound inducing 50% inhibition of cell viability relative to the viability in the untreated cultures. Apoptosis analyses were performed using propidium iodide (PI) staining, as described by Mauro et al. (2011). Additional details are provided in Supplemental Experimental Procedures.

Peptide Synthesis and ELISA
Proteins were purified as described by Tornatore et al. (2008). The combinatorial L-tetrapeptide libraries and individual peptides were synthesized as reported by Sandomenico et al. (2012). The methods used to assess peptide identity and purity and deconvolute the tetrapeptide libraries and the ELISA GADD45β/MKK7 competition assays were described previously (Sandomenico et al., 2012; Tornatore et al., 2008). IC_{50} values were defined as the mean concentration of peptide inducing 50% inhibition of GADD45β binding to MKK7 relative to the binding measured in the absence of peptide. Additional details are provided in Supplemental Experimental Procedures.

DTP3 Binding Assays
The stoichiometry and K_D value of the DTP3/MKK7 interaction were determined by tryptophan fluorescence quenching analysis, after fitting the fluorescence data with a nonlinear regression algorithm, as described by Williamson (2013). Additional details are provided in Supplemental Experimental Procedures, along with a description of the methods used for CD and MALDI-TOF mass spectrometry analyses and the pull-down of MKK7 from cells with DTP3.

Pharmacophore Analyses and Modeling
See Supplemental Experimental Procedures.

Pharmacokinetic Analyses
The pharmacokinetic analyses of DTP3 and z-DTP2 were outsourced. Additional details are provided in Supplemental Experimental Procedures.

Animal Studies
Mice were housed in the animal facilities at Hammersmith. All experiments were conducted under Procedure Project License (PPL) 70/6874, after approval by the Imperial College Ethical Review Process and the Home Office. For the plasmacytoma model, nonobese diabetic/severe combined immunodeficiency mice (NOD.CB17-Prkdc^{scid}/IcrCrl; Charles River) were injected subcutaneously with 1.0×10^7 U266 or KMS-11 MM cells and then randomized into treatment groups and treated by infusion, as shown. Tumor volumes were measured as described by Mauro et al. (2011). For the orthotopic MM model, mice of the same strain were sublethally irradiated and then injected intravenously with 1.0×10^7 KMS-12-BM MM cells, as described by Rabin et al. (2007). Mice were then randomized into treatment groups and treated by infusion for 8 weeks, as shown. Animals were monitored daily and euthanized on day 161 of treatment start or when they reached any of the end points in the PPL. Additional details are provided in Supplemental Experimental Procedures.

Statistical Analyses
See Supplemental Experimental Procedures.

SUPPLEMENTAL INFORMATION

Supplemental Information includes Supplemental Experimental Procedures, seven figures, and six tables and can be found with this article online at http://dx.doi.org/10.1016/j.ccr.2014.07.027.

AUTHOR CONTRIBUTIONS

L.T., G.F., M.R., D.R., C.L. and A.R. designed experiments. L.T., A.S., D.R., C.L., D.C., A.K.T., and A.C. performed experiments. L.T., G.F., M.R., A.S., D.R., C.L., A.R., C.T.-S., D.C., D.D., E.B., M.v.D., P.Z., A.J.-C., A.C., A.T., and A.R. analyzed data. M.B., B.A.W., A.L., C.D., P.S., G.M., A.P., and A.R. contributed clinical samples or key reagents. L.T., A.K.T., and J.D. contributed to mouse studies. G.F. wrote the paper. M.R., L.T., D.R., C.L., A.R., and A.T. contributed to writing the paper. A.S., D.R., and C.L. contributed equally to this work.

ACKNOWLEDGMENTS

We thank G. Inghirami, F. Dazzi, R. Orlowski, I. Kuiatse, L. Bergsagel, H. Auner, K. Parzych, and A. Karadimitris for cell lines; L. De Colibus, A. De Simone, E. Bellone, and M. Gambella for analytical or technical assistance; G. Cruciani for access to FLAP; and P. Omedè for primary PC samples. We also thank A. Iavarone for critical experimental advice and U. Siebenlist, K. Kelly, M. Lenardo, M. Pagano, H. Walczak, G. Acton, L. Busino, A. De Simone, and E. Tate for critical reading of the manuscript. The work was supported in part by Medical Research Council grants G0901436 and MR/L005069/1, NIH grants CA084040 and CA098583, Cancer Research UK grants A8839 and A15115, and an Imperial Innovations grant to G.F.; Project MERIT FIRB No. RBNE08NKH7_003 to M.R.; King Abdullah University of Science and Technology award KUK-I1-012-43 and PRIN 20108XYHJS to A.T.; and a PRIN 2009 grant to A.P.

G.F., L.T., M.R., C.L., C.T.S., A.J.C., A.P. and A.R. are named inventors on patent applications that have arisen from this research. Some of these authors hold shares in, or retain financial interests relating to, a for-profit company, Kesios Therapeutics, Ltd. G.F. is a Director of Kesios Therapeutics, Ltd. M.R. and L.T. are consultants for Kesios Therapeutics, Ltd.

Received: February 21, 2014
Revised: May 26, 2014
Accepted: July 29, 2014
Published: October 13, 2014

REFERENCES

Annunziata, C.M., Davis, R.E., Demchenko, Y., Bellamy, W., Gabrea, A., Zhan, F., Lenz, G., Hanamura, I., Wright, G., Xiao, W., et al. (2007). Frequent engagement of the classical and alternative NF-kappaB pathways by diverse genetic abnormalities in multiple myeloma. Cancer Cell *12*, 115–130.

Bjorklund, C.C., Ma, W., Wang, Z.Q., Davis, R.E., Kuhn, D.J., Kornblau, S.M., Wang, M., Shah, J.J., and Orlowski, R.Z. (2011). Evidence of a role for activation of Wnt/beta-catenin signaling in the resistance of plasma cells to lenalidomide. J. Biol. Chem. *286*, 11009–11020.

Bjorklund, C.C., Baladandayuthapani, V., Lin, H.Y., Jones, R.J., Kuiatse, I., Wang, H., Yang, J., Shah, J.J., Thomas, S.K., Wang, M., et al. (2014). Evidence of a role for CD44 and cell adhesion in mediating resistance to lenalidomide in multiple myeloma: therapeutic implications. Leukemia *28*, 373–383.

Broyl, A., Hose, D., Lokhorst, H., de Knegt, Y., Peeters, J., Jauch, A., Bertsch, U., Buijs, A., Stevens-Kroef, M., Beverloo, H.B., et al. (2010). Gene expression profiling for molecular classification of multiple myeloma in newly diagnosed patients. Blood *116*, 2543–2553.

Chapman, M.A., Lawrence, M.S., Keats, J.J., Cibulskis, K., Sougnez, C., Schinzel, A.C., Harview, C.L., Brunet, J.P., Ahmann, G.J., Adli, M., et al. (2011). Initial genome sequencing and analysis of multiple myeloma. Nature *471*, 467–472.

Chen, D., Frezza, M., Schmitt, S., Kanwar, J., and Dou, Q.P. (2011). Bortezomib as the first proteasome inhibitor anticancer drug: current status and future perspectives. Curr. Cancer Drug Targets *11*, 239–253.

Chi, H., Lu, B., Takekawa, M., Davis, R.J., and Flavell, R.A. (2004). GADD45β/GADD45γ and MEKK4 comprise a genetic pathway mediating STAT4-independent IFNγ production in T cells. EMBO J. *23*, 1576–1586.

Chou, T.C. (2006). Theoretical basis, experimental design, and computerized simulation of synergism and antagonism in drug combination studies. Pharmacol. Rev. *58*, 621–681.

Davies, C., and Tournier, C. (2012). Exploring the function of the JNK (c-Jun N-terminal kinase) signalling pathway in physiological and pathological processes to design novel therapeutic strategies. Biochem. Soc. Trans. *40*, 85–89.

Demchenko, Y.N., Glebov, O.K., Zingone, A., Keats, J.J., Bergsagel, P.L., and Kuehl, W.M. (2010). Classical and/or alternative NF-kappaB pathway activation in multiple myeloma. Blood *115*, 3541–3552.

De Smaele, E., Zazzeroni, F., Papa, S., Nguyen, D.U., Jin, R., Jones, J., Cong, R., and Franzoso, G. (2001). Induction of gadd45β by NF-kappaB downregulates pro-apoptotic JNK signalling. Nature *414*, 308–313.

Dickens, N.J., Walker, B.A., Leone, P.E., Johnson, D.C., Brito, J.L., Zeisig, A., Jenner, M.W., Boyd, K.D., Gonzalez, D., Gregory, W.M., et al. (2010). Homozygous deletion mapping in myeloma samples identifies genes and an expression signature relevant to pathogenesis and outcome. Clin. Cancer Res. *16*, 1856–1864.

DiDonato, J.A., Mercurio, F., and Karin, M. (2012). NF-κB and the link between inflammation and cancer. Immunol. Rev. *246*, 379–400.

Gomez-Bougie, P., and Amiot, M. (2013). Apoptotic machinery diversity in multiple myeloma molecular subtypes. Front. Immunol. *4*, 467.

Hideshima, T., Chauhan, D., Richardson, P., and Anderson, K.C. (2005). Identification and validation of novel therapeutic targets for multiple myeloma. J. Clin. Oncol. *23*, 6345–6350.

Houghten, R.A., Pinilla, C., Appel, J.R., Blondelle, S.E., Dooley, C.T., Eichler, J., Nefzi, A., and Ostresh, J.M. (1999). Mixture-based synthetic combinatorial libraries. J. Med. Chem. *42*, 3743–3778.

Keats, J.J., Fonseca, R., Chesi, M., Schop, R., Baker, A., Chng, W.J., Van Wier, S., Tiedemann, R., Shi, C.X., Sebag, M., et al. (2007). Promiscuous mutations activate the noncanonical NF-kappaB pathway in multiple myeloma. Cancer Cell *12*, 131–144.

Kuehl, W.M., and Bergsagel, P.L. (2002). Multiple myeloma: evolving genetic events and host interactions. Nat. Rev. Cancer *2*, 175–187.

Kyle, R.A., and Rajkumar, S.V. (2009). Criteria for diagnosis, staging, risk stratification and response assessment of multiple myeloma. Leukemia *23*, 3–9.

Lam, L.T., Davis, R.E., Pierce, J., Hepperle, M., Xu, Y., Hottelet, M., Nong, Y., Wen, D., Adams, J., Dang, L., and Staudt, L.M. (2005). Small molecule inhibitors of IkappaB kinase are selectively toxic for subgroups of diffuse large B-cell lymphoma defined by gene expression profiling. Clin. Cancer Res. *11*, 28–40.

Lu, B., Ferrandino, A.F., and Flavell, R.A. (2004). Gadd45β is important for perpetuating cognate and inflammatory signals in T cells. Nat. Immunol. *5*, 38–44.

Mahindra, A., Laubach, J., Raje, N., Munshi, N., Richardson, P.G., and Anderson, K. (2012). Latest advances and current challenges in the treatment of multiple myeloma. Nat Rev Clin Oncol *9*, 135–143.

Mauro, C., Leow, S.C., Anso, E., Rocha, S., Thotakura, A.K., Tornatore, L., Moretti, M., De Smaele, E., Beg, A.A., Tergaonkar, V., et al. (2011). NF-κB controls energy homeostasis and metabolic adaptation by upregulating mitochondrial respiration. Nat. Cell Biol. *13*, 1272–1279.

McCurdy, A.R., and Lacy, M.Q. (2013). Pomalidomide and its clinical potential for relapsed or refractory multiple myeloma: an update for the hematologist. Ther Adv Hematol *4*, 211–216.

Ngo, V.N., Davis, R.E., Lamy, L., Yu, X., Zhao, H., Lenz, G., Lam, L.T., Dave, S., Yang, L., Powell, J., and Staudt, L.M. (2006). A loss-of-function RNA interference screen for molecular targets in cancer. Nature *441*, 106–110.

Nickl, C.K., Raidas, S.K., Zhao, H., Sausbier, M., Ruth, P., Tegge, W., Brayden, J.E., and Dostmann, W.R. (2010). (D)-Amino acid analogues of DT-2 as highly selective and superior inhibitors of cGMP-dependent protein kinase Ialpha. Biochim. Biophys. Acta *1804*, 524–532.

Palumbo, A., Bringhen, S., Rossi, D., Cavalli, M., Larocca, A., Ria, R., Offidani, M., Patriarca, F., Nozzoli, C., Guglielmelli, T., et al. (2010). Bortezomib-melphalan-prednisone-thalidomide followed by maintenance with bortezomib-thalidomide compared with bortezomib-melphalan-prednisone for initial treatment of multiple myeloma: a randomized controlled trial. J. Clin. Oncol. *28*, 5101–5109.

Papa, S., Zazzeroni, F., Bubici, C., Jayawardena, S., Alvarez, K., Matsuda, S., Nguyen, D.U., Pham, C.G., Nelsbach, A.H., Melis, T., et al. (2004). Gadd45 β mediates the NF-κ B suppression of JNK signalling by targeting MKK7/JNKK2. Nat. Cell Biol. *6*, 146–153.

Papa, S., Monti, S.M., Vitale, R.M., Bubici, C., Jayawardena, S., Alvarez, K., De Smaele, E., Dathan, N., Pedone, C., Ruvo, M., and Franzoso, G. (2007). Insights into the structural basis of the GADD45β-mediated inactivation of the JNK kinase, MKK7/JNKK2. J. Biol. Chem. *282*, 19029–19041.

Papa, S., Zazzeroni, F., Fu, Y.X., Bubici, C., Alvarez, K., Dean, K., Christiansen, P.A., Anders, R.A., and Franzoso, G. (2008). Gadd45β promotes hepatocyte survival during liver regeneration in mice by modulating JNK signaling. J. Clin. Invest. *118*, 1911–1923.

Piva, R., Ruggeri, B., Williams, M., Costa, G., Tamagno, I., Ferrero, D., Giai, V., Coscia, M., Peola, S., Massaia, M., et al. (2008). CEP-18770: A novel, orally active proteasome inhibitor with a tumor-selective pharmacologic profile competitive with bortezomib. Blood *111*, 2765–2775.

Rabin, N., Kyriakou, C., Coulton, L., Gallagher, O.M., Buckle, C., Benjamin, R., Singh, N., Glassford, J., Otsuki, T., Nathwani, A.C., et al. (2007). A new xenograft model of myeloma bone disease demonstrating the efficacy of human mesenchymal stem cells expressing osteoprotegerin by lentiviral gene transfer. Leukemia *21*, 2181–2191.

Rajkumar, S.V. (2011). Treatment of multiple myeloma. Nat. Rev. Clin. Oncol. *8*, 479–491.

Richardson, P.G. (2010). Improving the therapeutic index in myeloma. Blood *116*, 4733–4734.

Rückrich, T., Kraus, M., Gogel, J., Beck, A., Ovaa, H., Verdoes, M., Overkleeft, H.S., Kalbacher, H., and Driessen, C. (2009). Characterization of the ubiquitin-proteasome system in bortezomib-adapted cells. Leukemia *23*, 1098–1105.

Sandomenico, A., Russo, A., Palmieri, G., Bergamo, P., Gogliettino, M., Falcigno, L., and Ruvo, M. (2012). Small peptide inhibitors of acetyl-peptide hydrolase having an uncommon mechanism of inhibition and a stable bent conformation. J. Med. Chem. *55*, 2102–2111.

Staudt, L.M. (2010). Oncogenic activation of NF-kappaB. Cold Spring Harb. Perspect. Biol. *2*, a000109.

Tang, G., Minemoto, Y., Dibling, B., Purcell, N.H., Li, Z., Karin, M., and Lin, A. (2001). Inhibition of JNK activation through NF-kappaB target genes. Nature *414*, 313–317.

Tornatore, L., Marasco, D., Dathan, N., Vitale, R.M., Benedetti, E., Papa, S., Franzoso, G., Ruvo, M., and Monti, S.M. (2008). Gadd45 β forms a homodimeric complex that binds tightly to MKK7. J. Mol. Biol. *378*, 97–111.

Tracey, L., Pérez-Rosado, A., Artiga, M.J., Camacho, F.I., Rodríguez, A., Martínez, N., Ruiz-Ballesteros, E., Mollejo, M., Martinez, B., Cuadros, M., et al. (2005). Expression of the NF-kappaB targets BCL2 and BIRC5/Survivin characterizes small B-cell and aggressive B-cell lymphomas, respectively. J. Pathol. *206*, 123–134.

Williamson, M.P. (2013). Using chemical shift perturbation to characterise ligand binding. Prog. Nucl. Magn. Reson. Spectrosc. *73*, 1–16.

Zhang, N., Ahsan, M.H., Zhu, L., Sambucetti, L.C., Purchio, A.F., and West, D.B. (2005). NF-kappaB and not the MAPK signaling pathway regulates GADD45β expression during acute inflammation. J. Biol. Chem. *280*, 21400–21408.

Zhou, N., Luo, Z., Luo, J., Fan, X., Cayabyab, M., Hiraoka, M., Liu, D., Han, X., Pesavento, J., Dong, C.Z., et al. (2002). Exploring the stereochemistry of CXCR4-peptide recognition and inhibiting HIV-1 entry with D-peptides derived from chemokines. J. Biol. Chem. *277*, 17476–17485.

Cancer Cell, Volume *26*

Supplemental Information

Cancer-Selective Targeting of the NF-κB Survival Pathway with GADD45β/MKK7 Inhibitors

Laura Tornatore, Annamaria Sandomenico, Domenico Raimondo, Caroline Low, Alberto Rocci, Cathy Tralau-Stewart, Daria Capece, Daniel D'Andrea, Marco Bua, Eileen Boyle, Mark van Duin, Pietro Zoppoli, Albert Jaxa-Chamiec, Anil K. Thotakura, Julian Dyson, Brian A. Walker, Antonio Leonardi, Angela Chambery, Christoph Driessen, Pieter Sonneveld, Gareth Morgan, Antonio Palumbo, Anna Tramontano, Amin Rahemtulla, Menotti Ruvo, and Guido Franzoso

SUPPLEMENTAL DATA

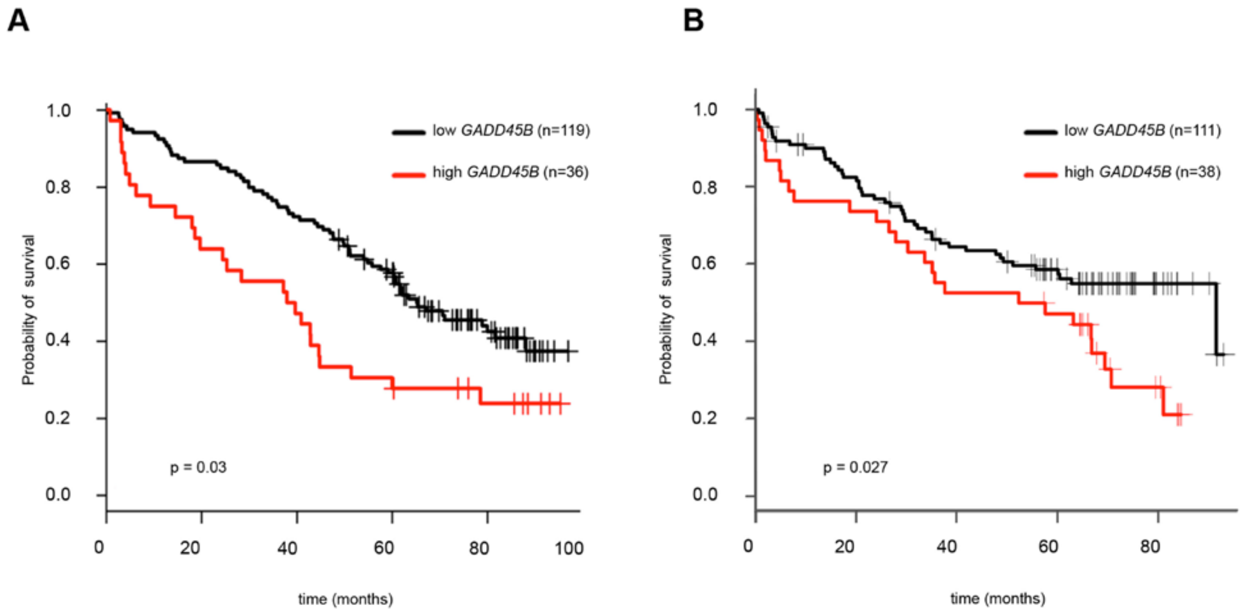

Figure S1, related to Figure 1. Correlation between high *GADD45B* expression and poor clinical outcome in two independent datasets of newly diagnosed MM patients

(**A**, **B**) OS in MM patients from the intensive arm of the MRC Myeloma IX trial (GSE15695; n=155) (Dickens et al., 2010; Wu et al., 2011) (**A**) and the VAD treatment arm of the HOVON-65/GMMG-HD4 trial (GSE19784; n=149) (Broyl et al., 2010, Sonneveld et al., 2008) (**B**) stratified in each case at diagnosis using k-mean clustering according to the *GADD45B* expression value (Hartigan and Wong, 1979).

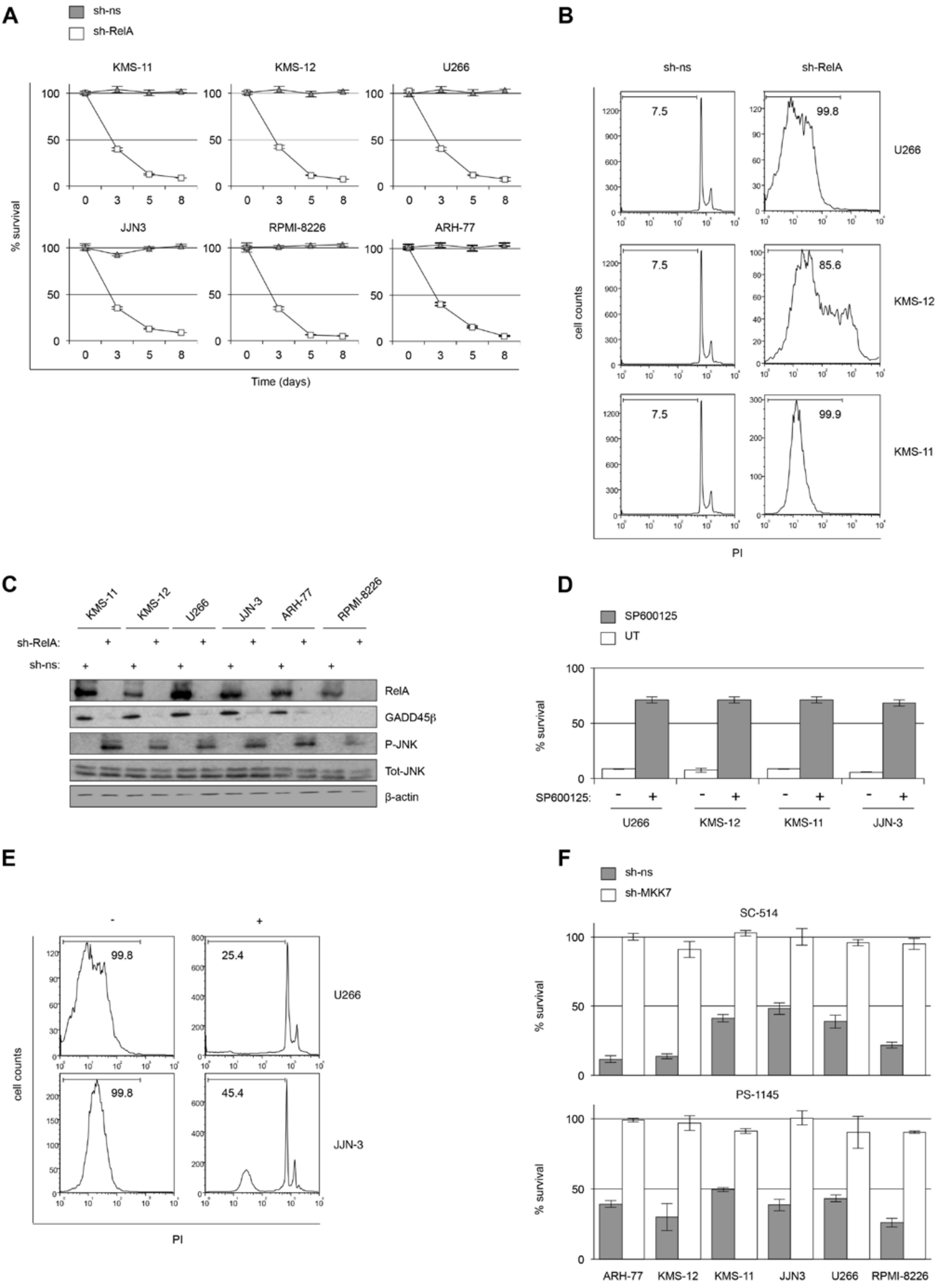
A
sh-ns
sh-RelA
KMS-11
KMS-12
U266
JJN3
RPMI-8226
ARH-77
% survival
Time (days)
B
sh-ns
sh-RelA
U266
KMS-12
KMS-11
7.5
99.8
85.6
99.9
cell counts
PI
C
KMS-11
KMS-12
U266
JJN-3
ARH-77
RPMI-8226
sh-RelA:
sh-ns:
RelA
GADD45β
P-JNK
Tot-JNK
β-actin
D
SP600125
UT
% survival
SP600125:
U266
KMS-12
KMS-11
JJN-3
E
U266
JJN-3
99.8
25.4
45.4
cell counts
PI
F
sh-ns
sh-MKK7
SC-514
PS-1145
% survival
ARH-77
KMS-12
KMS-11
JJN3
U266
RPMI-8226

G

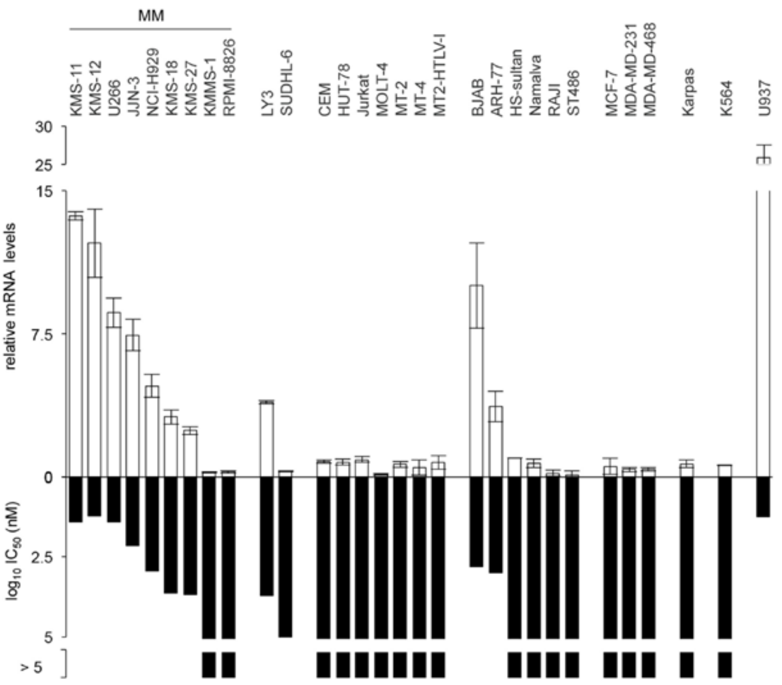
MM
KMS-11
KMS-12
U266
JJN-3
NCI-H929
KMS-18
KMS-27
KMMS-1
RPMI-8826
LY3
SUDHL-6
CEM
HUT-78
Jurkat
MOLT-4
MT-2
MT-4
MT2-HTLV-I
BJAB
ARH-77
HS-sultan
Namalva
RAJI
ST486
MCF-7
MDA-MD-231
MDA-MD-468
Karpas
K564
U937
relative mRNA levels
log_{10} IC_{50} (nM)
30
25
15
7.5
0
2.5
5
> 5

H

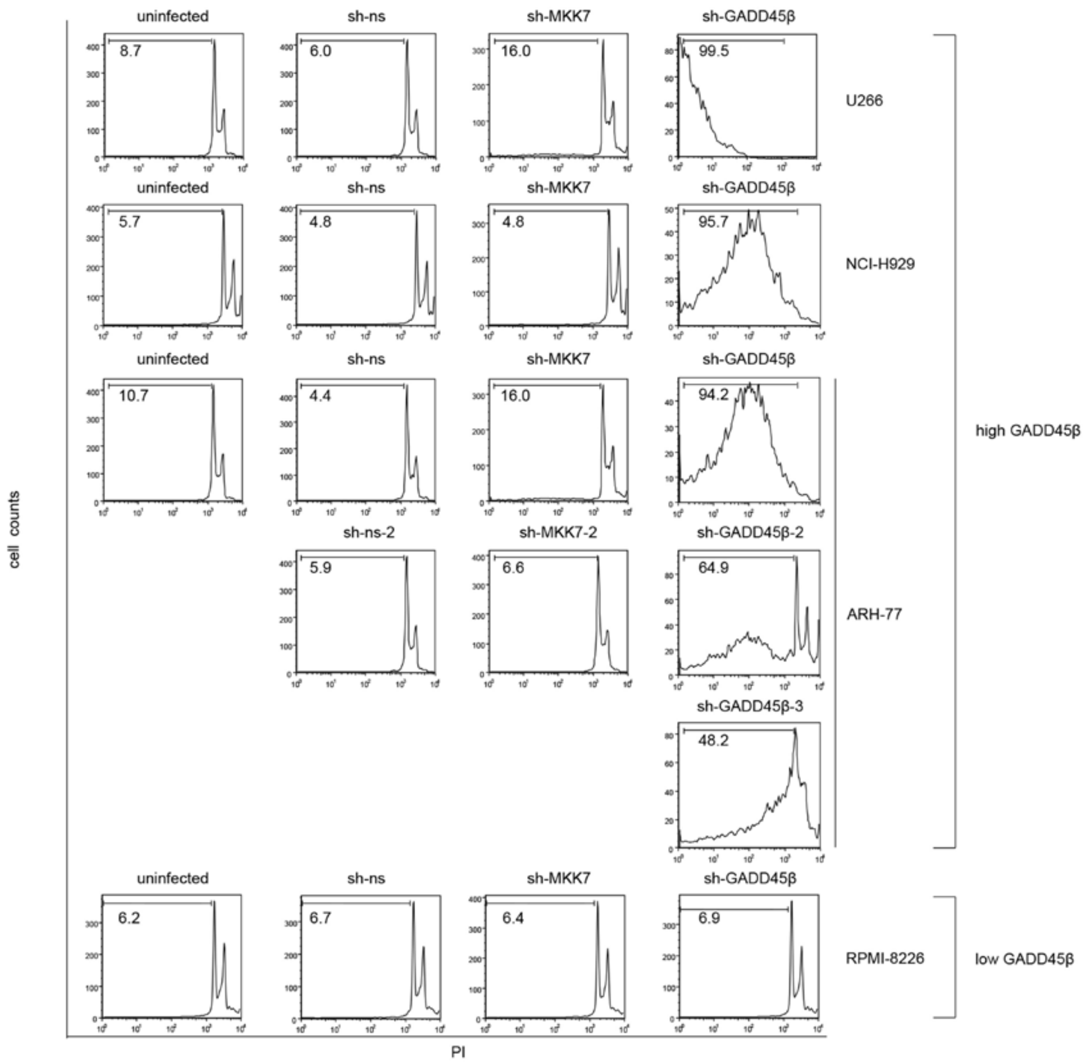
uninfected
sh-ns
sh-MKK7
sh-GADD45β
8.7
6.0
16.0
99.5
U266
5.7
4.8
4.8
95.7
NCI-H929
10.7
4.4
16.0
94.2
sh-ns-2
sh-MKK7-2
sh-GADD45β-2
5.9
6.6
64.9
ARH-77
sh-GADD45β-3
48.2
high GADD45β
6.2
6.7
6.4
6.9
RPMI-8226
low GADD45β
cell counts
PI

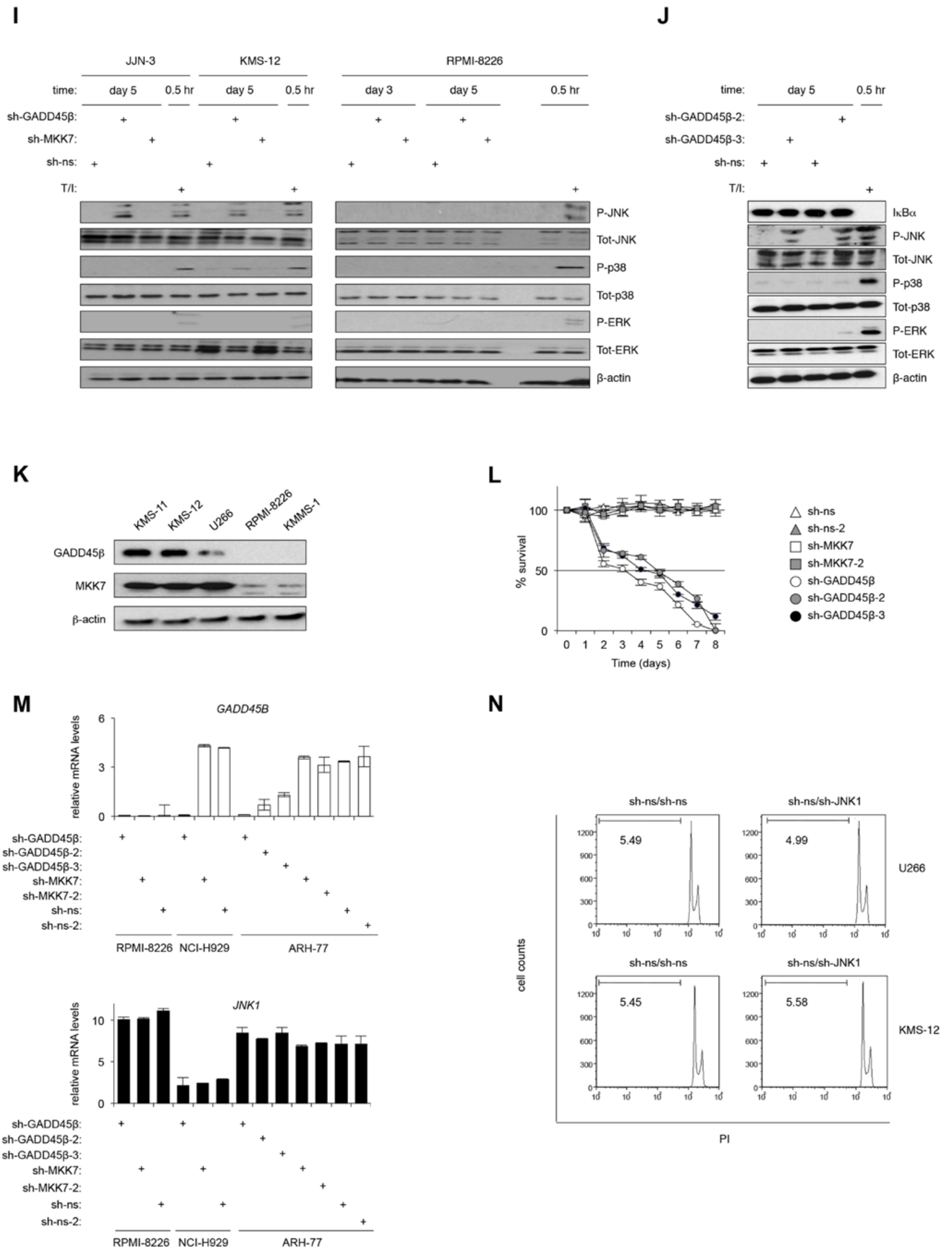
I
J
JJN-3
KMS-12
RPMI-8226
time:
day 5
0.5 hr
day 3
sh-GADD45β:
sh-MKK7:
sh-ns:
T/I:
P-JNK
Tot-JNK
P-p38
Tot-p38
P-ERK
Tot-ERK
β-actin
sh-GADD45β-2:
sh-GADD45β-3:
IκBα
K
KMS-11
U266
KMMS-1
GADD45β
MKK7
L
% survival
Time (days)
sh-ns
sh-ns-2
sh-MKK7
sh-MKK7-2
sh-GADD45β
sh-GADD45β-2
sh-GADD45β-3
M
GADD45B
relative mRNA levels
sh-MKK7-2:
NCI-H929
ARH-77
JNK1
N
sh-ns/sh-ns
sh-ns/sh-JNK1
5.49
4.99
5.45
5.58
U266
cell counts
PI

O

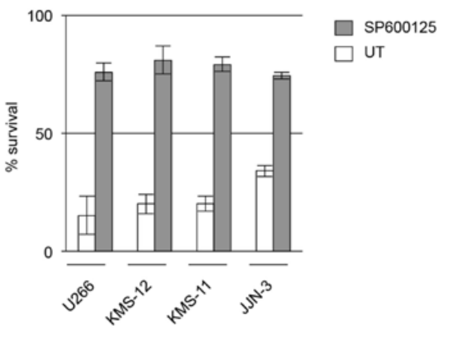
100
50
0
% survival
SP600125
UT
U266
KMS-12
KMS-11
JJN-3

P

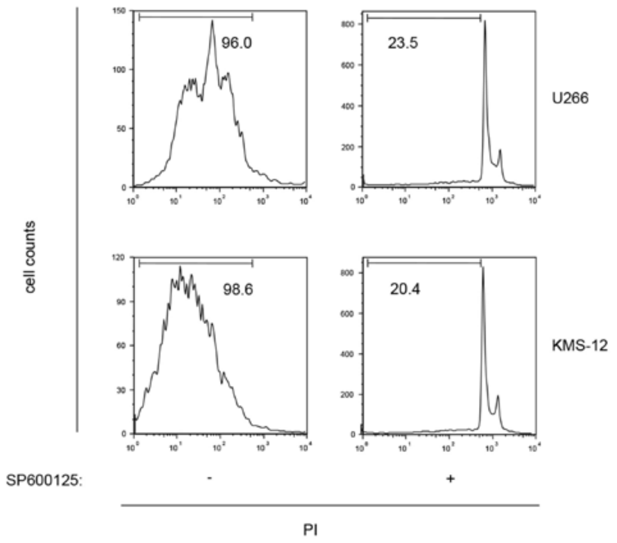
cell counts
96.0
23.5
U266
98.6
20.4
KMS-12
SP600125:
-
+
PI

Figure S2, related to Figure 2. NF-κB promotes the survival of MM cells by inhibiting JNK signalling and apoptosis by means of GADD45β

(**A**) Trypan blue exclusion assays showing the survival of MM cell lines expressing non-specific (sh-ns) or RelA-specific (sh-RelA) sh-RNAs.

(**B**) Propidium iodide (PI) nuclear staining assays showing apoptotic cells (i.e. cells exhibiting sub-G_1 DNA content) in representative cell lines from (**A**) at day 6.

(**C**) Western blots showing RelA, GADD45β and total and phosphorylated (P) JNK in MM cell lines. Also shown are the knockdown efficiency (RelA) and specificity (total JNK, β-actin) of the sh-RNAs.

(**D**) Trypan blue exclusion assays showing the survival of representative MM cell lines expressing sh-RelA and cultured in the presence (+) or absence (-) of SP600125 for 6 days. UT, untreated.

(**E**) PI nuclear staining assays showing apoptotic cells in representative cell lines from (**D**).

(**F**) [^{3}H]Thymidine incorporation assays showing the survival of MM cell lines expressing sh-ns or sh-MKK7 after treatment for 6 days with the IKKβ inhibitors, SC-514 (top) or PS-1145 (bottom). Values express the percentage of the counts per minute (cpm) measured with the treated cultures relative to the cpm measured with the respective untreated cultures.

(**G**) Shown are the relative *GADD45B* mRNA expression levels (qRT-PCR assays; top) and the IC_{50}s of DTP3 at 144 hr ([^{3}H]thymidine incorporation assays; bottom) in a panel of tumor cell lines of different tissues of origin. Values express the logarithm to base 10 (log_{10}) of the IC_{50} (bottom). A similar correlation between D-peptide cellular activity and *GADD45B* mRNA expression in cancer cells was observed with the active precursors of DTP3, z-DTP2 and z-DTP1.

(**H**) PI nuclear staining assays showing apoptotic cells in representative MM cell lines (day 8). Also shown are the results with uninfected cells. The experiment with the ARH-77 B lymphoblastoid cell line further shows the results obtained with one additional non-specific sh-RNA (sh-ns-2), one additional MKK7-specific sh-RNA (sh-MKK7-2), and two additional GADD45β-specific sh-RNAs (sh-GADD45β-2, sh-GADD45β-3).

(**I**) Western blots showing total and phosphorylated (P) proteins in representative MM cell lines, expressing sh-ns, sh-MKK7 or sh-GADD45β sh-RNAs.

(**J**) Western blots showing total and phosphorylated (P) proteins in U266 MM cells expressing sh-ns, sh-GADD45β-2 or sh-GADD45β-3.

(**K**) Western blots showing the expression of GADD45β and MKK7 in representative GADD45β-dependent (KMS-11, KMS-12, U266) and GADD45β-independent (RPMI-8226, KMM-1) MM cell lines.

(**L**) Trypan blue exclusion assays showing the survival of ARH-77 B lymphoblastoid cells expressing different non-specific (sh-ns, sh-ns-2), GADD45β-specific (sh-GADD45β, sh-GADD45β-2, sh-GADD45β-3) or MKK7-specific (sh-MKK7, sh-MKK7-2) sh-RNAs.

(**M**) qRT-PCR assays showing the relative *GADD45B* mRNA expression levels in the MM (NCI-H929, RPMI-8226) and non-MM (ARH-77) cell lines expressing sh-ns, sh-GADD45β or sh-MKK7 sh-RNAs (top). JNK1 is shown as specificity control (bottom).

(**N**) PI nuclear labelling assays showing apoptotic cells in representative MM cell lines co-expressing sh-ns and sh-ns (sh-ns/sh-ns) or sh-ns and sh-JNK1 (sh-ns/sh-JNK1) on day 8 after lentivirus infection.

(**O**) Trypan blue exclusion assays showing the survival of representative MM cell lines expressing sh-GADD45β and left untreated (UT; -) or treated with SP600125 (+) for 6 days.

(**P**) PI nuclear labelling assays showing apoptotic cells in representative MM cell lines from (**O**) expressing sh-GADD45β sh-RNAs and left untreated (-) or treated (+) with SP600125 for 6 days.

(**A**, **C**, **F**) Note that the ARH-77 cell line is an EBV transformed B lymphoblastoid cell line, and not a MM cell line, and is shown for the purpose of establishing the dependence of GADD45β expression on constitutive NF-κB activity also in non-MM tumor cell lines.

(**A**, **D**, **F**, **G**, **M**, **O**) Values denote means ± SD (n=3).

(**A**, **D**, **L**, **O**) Values express the percentages of live enhanced green fluorescent protein $(eGFP)^+$ cells present in the cultures at the times shown (**A**, **L**) or at day 6 (**D**, **O**) relative to the number of live $eGFP^+$ cells present in the respective cultures at day 0.

(**B**, **E**, **H**, **N**, **P**) The percentages of apoptotic cells are depicted.

(**C**, **I**, **J**, **K**) β-actin is shown as control.

(**I**, **J**) T/I, 12-O-tetradecanoylphorbol-13-acetate (TPA)/ionomycin.

A

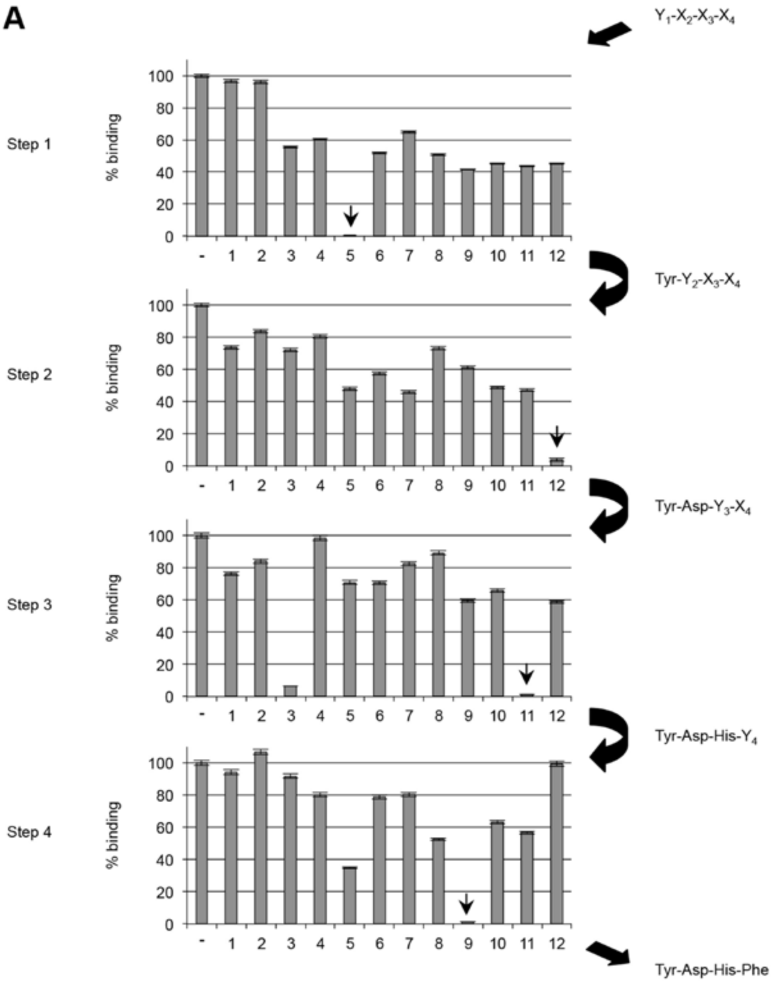

Y_1-X_2-X_3-X_4
Step 1
Tyr-Y_2-X_3-X_4
Step 2
Tyr-Asp-Y_3-X_4
Step 3
Tyr-Asp-His-Y_4
Step 4
Tyr-Asp-His-Phe
% binding

B

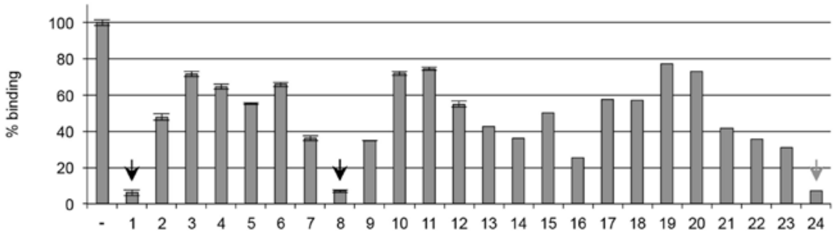

% binding

Figure S3, related to Figure 3. The iterative ELISA screen used for the isolation of the bioactive L-tetrapeptides, Ac-LTP1 and Ac-LTP2

(**A**) Antagonists of the GADD45β/MKK7 interaction were selected by screening a simplified combinatorial L-tetrapeptide library of general core formula Y_1-X_2-X_3-X_4, containing all possible combinations of 12 L-amino acids representing different chemical spaces, at each position from X_1 to X_4 (see Table S1). This library, consisting of a total of 12^4 = 20,736 different L-tetrapeptides, harboring a C-terminal amide group and an N-terminal Fmoc-(β-Ala)$_2$ tag, was iteratively deconvoluted in four steps in ELISA GADD45β/MKK7 competition assays, using at each step, coated human (h)MKK7 fused to GST (GST-hMKK7), soluble biotin-labelled His$_6$-hGADD45β, and each of the 12 sub-libraries. Shown are the results from the screen and the core amino acid sequences of the peptide mixtures used in steps 1 to 4, using the standard three-letter amino acid code. Y_1, Y_2, Y_3 and Y_4, each of the 12 amino acids used in steps 1-4; X_2, X_3 and X_4, mixtures of the 12 amino acids used in steps 1-3. Arrows denote the peptide mixtures or pure peptides chosen for the subsequent steps of the screen.

(**B**) The most active peptide resulting from (**A**), namely Tyr-Asp-His-Phe (step 4, column 9), was used as precursor for the synthesis of an additional set of 24 L-tetrapeptides, in which the N-terminal Fmoc-(β-Ala)$_2$ tag was replaced with an acetyl (Ac) group, and amino acid residues excluded in (**A**) were also included (Table S2). These second-generation L-tetrapeptides were screened by ELISA GADD45β/MKK7 competition assays as in (**A**), yielding Ac-Tyr-Asp-His-Phe-NH_2 (Ac-LTP1) and Ac-Tyr-Glu-Arg-Phe-NH_2 (Ac-LTP2) (columns 1 and 8, respectively; black arrows), which were then used in subsequent studies. Peptide 24 (grey arrow) was excluded from further analyses because of low aqueous solubility (Table S2).

(**A**, **B**) Values express the percentage of inhibition of GADD45β binding to GST-MKK7 relative to the binding measured in the absence of peptide (-) and denote means ± SD (n=3).

Table S1, related to Figure 3. List of the tetrapeptide mixtures and individual peptides used for the initial ELISA screen

	amino acid sequence	% inhibition (ELISA)
Step 1	$QX_2X_3X_4$	3
	$SX_2X_3X_4$	4
	$RX_2X_3X_4$	44
	$AX_2X_3X_4$	40
	$YX_2X_3X_4$	100
	$PX_2X_3X_4$	48
	$MX_2X_3X_4$	35
	$CX_2X_3X_4$	49
	$FX_2X_3X_4$	58
	$LX_2X_3X_4$	55
	$HX_2X_3X_4$	56
	$DX_2X_3X_4$	55
Step 2	YQX_3X_4	26
	YSX_3X_4	16
	YRX_3X_4	28
	YAX_3X_4	20
	YYX_3X_4	52
	YPX_3X_4	42
	YMX_3X_4	54
	YCX_3X_4	27
	YFX_3X_4	39
	YLX_3X_4	52
	YHX_3X_4	53
	YDX_3X_4	96
Step 3	$YDQX_4$	24
	$YDSX_4$	16
	$YDRX_4$	93
	$YDAX_4$	2
	$YDYX_4$	29
	$YDPX_4$	29
	$YDMX_4$	17
	$YDCX_4$	11
	$YDFX_4$	41
	$YDLX_4$	34
	$YDHX_4$	99
	$YDDX_4$	41
Step 4	YDHQ	6
	YDHS	0
	YDHR	8
	YDHA	20
	YDHY	65
	YDHP	21
	YDHM	20
	YDHC	47
	YDHF (LTP1)	99
	YDHL	37
	YDHH	43
	YDHD	1

Shown is the core amino acid sequence of each of the peptide mixtures and individual peptides used for the ELISA screen depicted in Figure S3A. The simplified combinatorial library used for this screen consisted of a total of 12^4 = 20,736 L-tetrapeptides formed by all possible combinations of the following 12 L-amino acids: Gln (Q), Ser (S), Arg (R), Ala (A), Tyr (Y), Pro (P), Met (M), Cys(Acm) (C), Phe (F), Leu (L), His (H), Asp (D). Amino acids are identified by the standard single-letter amino acid code. X_2, X_3 and X_4 represent mixtures of the 12 amino acids listed above and used in steps 1 to 3 of the iterative screen (Figure S3A). Also shown are the results generated at each step of the library deconvolution process. Values express the percentage of inhibition of GADD45β binding to MKK7 obtained for each of the 12 peptide mixtures or individual peptides at the nominal concentration of 42 nM, relative to the binding measured in the absence of competitor and denote means (n=3).

Table S2, related to Figure 3. List of the tetrapeptides used for secondary screening

	amino acid sequence	MW	% inhibition (ELISA)
1	Ac-YDHF-NH_2 (Ac-LTP1)	621	94
2	Ac-YEHF-NH_2	635	52
3	Ac-WDHF-NH_2	644	28
4	Ac-WEHF-NH_2	658	35
5	Ac-YDRF-NH_2	640	45
6	Ac-YDKF-NH_2	612	34
7	Ac-YEKF-NH_2	626	64
8	Ac-YERF-NH_2 (Ac-LTP2)	654	93
9	Ac-WEKF-NH_2	649	65
10	Ac-WERF-NH_2	677	28
11	Ac-WDKF-NH_2	653	25
12	Ac-WDRF-NH_2	663	45
13	Ac-YDHW-NH_2	660	57
14	Ac-YEHW-NH_2	674	64
15	Ac-WDHW-NH_2	683	50
16	Ac-WEHW-NH_2	697	75
17	Ac-YDRW-NH_2	679	43
18	Ac-YDKW-NH_2	651	43
19	Ac-YEKW-NH_2	665	22
20	Ac-YERW-NH_2	693	27
21	Ac-WEKW-NH_2	688	58
22	Ac-WERW-NH_2	716	64
23	Ac-WDKW-NH_2	674	69
24	Ac-WDRW-NH_2	702	93

Shown are the 24 L-tetrapeptides of second generation used for the ELISA screen reported in Figure S3B. L-peptides were synthesized starting from the most active compound derived from step 4 of the screen shown in Figure S3A (column 9), namely Fmoc-(βAla)$_2$-YDHF-NH_2 (referred to as LTP1; Table S1), by replacing the Nterminal -Fmoc-(bAla)$_2$ tag with an acetyl (Ac) group and including additional L-amino acids not used for the synthesis of the L-tetrapeptides shown in Table S1 as follows: Tyr (Y) or Trp (W), in the first position; Asp (D) or Glu (E), in the second position; His (H), Arg (R) or Lys (K), in the third position; Phe (F) or Trp (W), in the fourth position. All peptides were C-terminally amidated and N-terminally acetylated, and were in the L configuration. Also shown are the results from the ELISA competition screen. Values express the percentage of inhibition of GADD45β binding to MKK7 obtained for each of the peptides at the concentration of 42 nM, relative to the binding measured in the absence of peptide and denote means (n=3). The most active L-tetrapeptides resulting from this screen were denoted as Ac-LTP1 (Ac-YDHF-NH_2) and Ac-LTP2 (Ac-YERF-NH_2) and were used for subsequent analyses. MW, molecular weight.

A

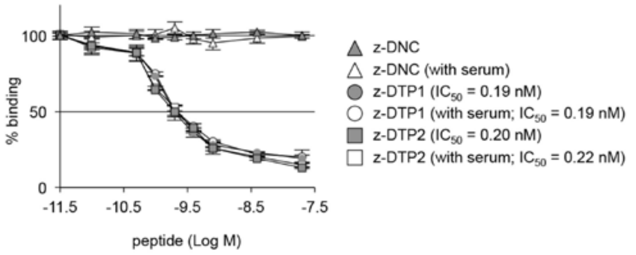

B

IC_{50} (ELISA)				
	Ac-DTP2	z-DTP2	Ac-DTP1	z-DTP1
without serum	0.22 nM	0.20 nM	0.19 nM	0.19 nM
with serum	0.22 nM	0.22 nM	0.20 nM	0.19 nM

C

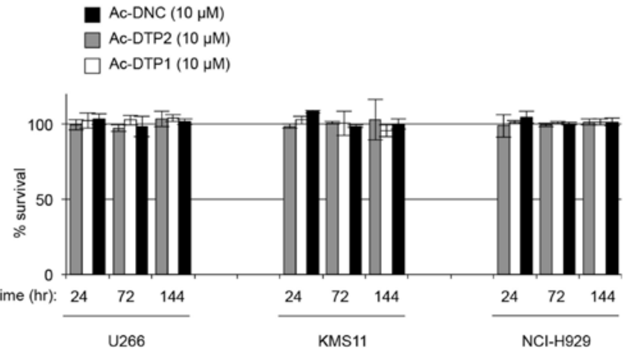

D

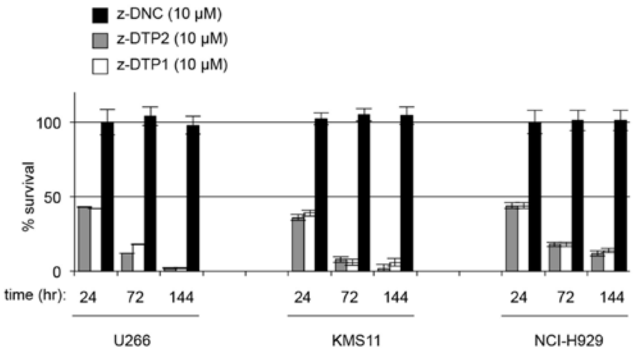

E

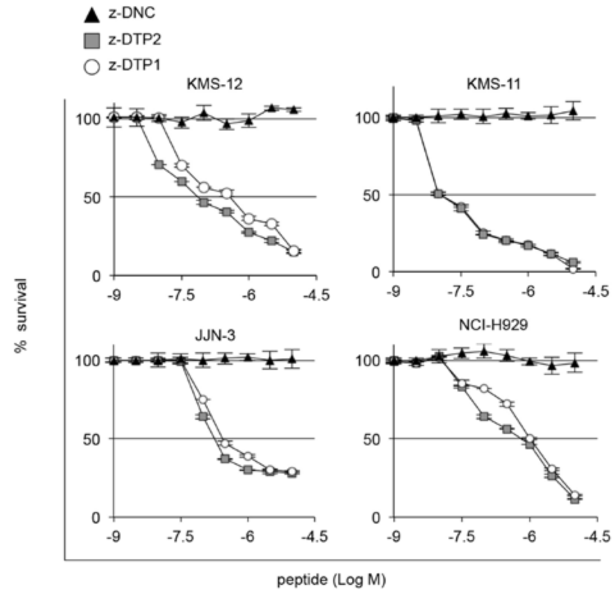

F

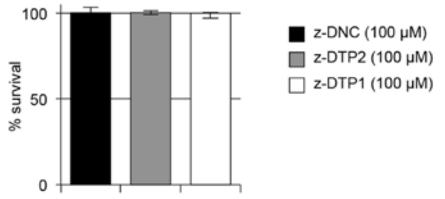

G

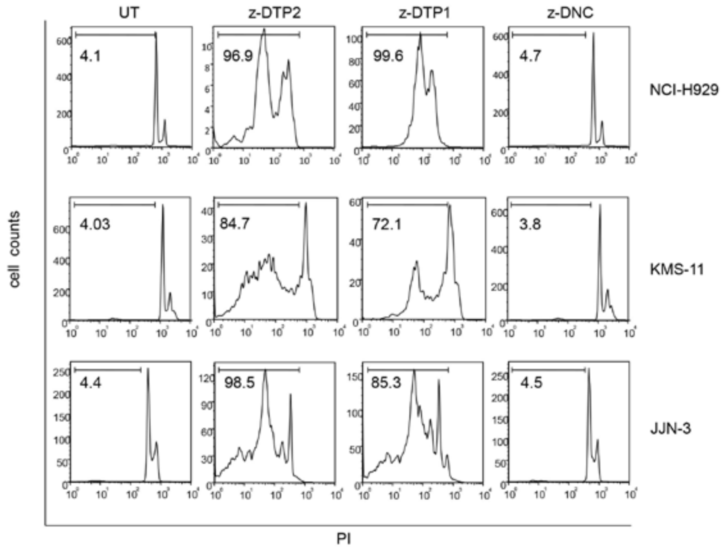
UT
z-DTP2
z-DTP1
z-DNC
NCI-H929
KMS-11
JJN-3
4.1
96.9
99.6
4.7
4.03
84.7
72.1
3.8
4.4
98.5
85.3
4.5
cell counts
PI

H

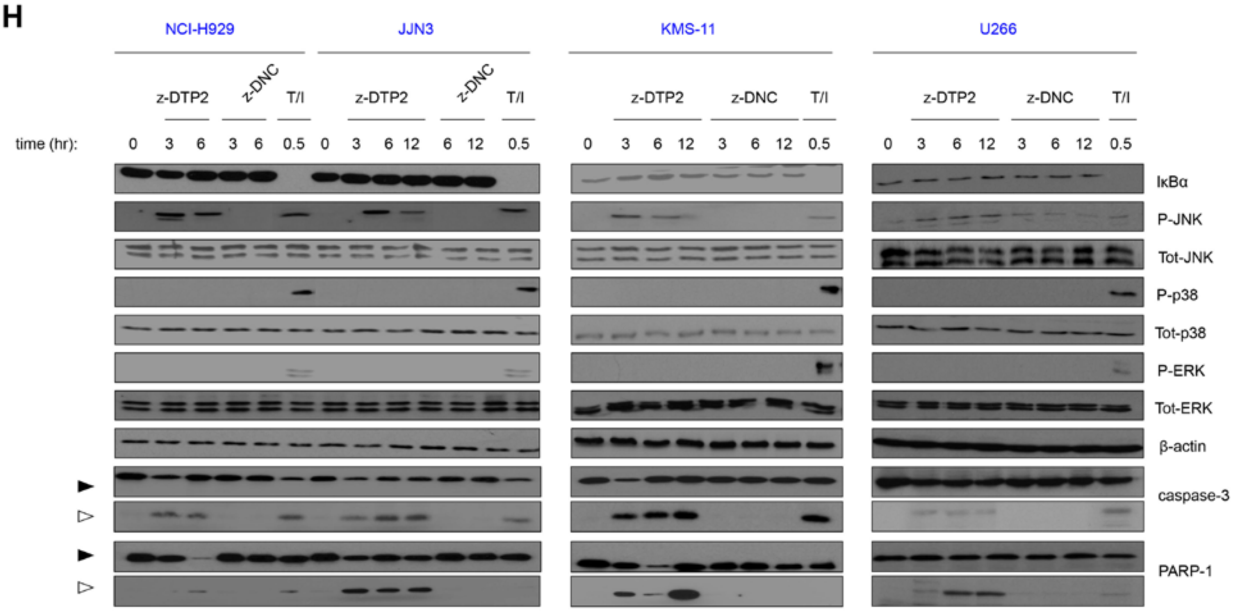
NCI-H929
JJN3
KMS-11
U266
z-DTP2
z-DNC
T/I
time (hr):
0 3 6 3 6 0.5 0 3 6 12 6 12 0.5
0 3 6 12 3 6 12 0.5
0 3 6 12 3 6 12 0.5
IκBα
P-JNK
Tot-JNK
P-p38
Tot-p38
P-ERK
Tot-ERK
β-actin
caspase-3
PARP-1

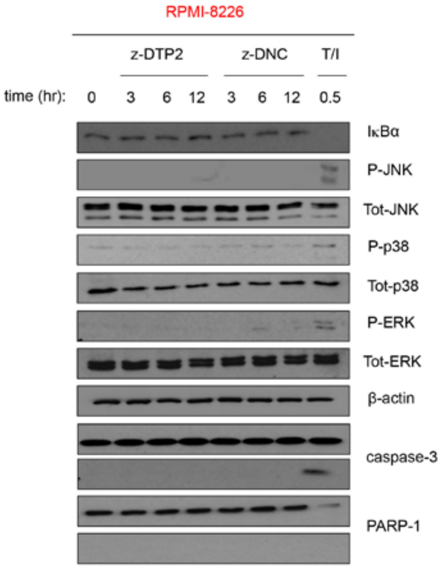
RPMI-8226
z-DTP2
z-DNC
T/I
time (hr):
0 3 6 12 3 6 12 0.5
IκBα
P-JNK
Tot-JNK
P-p38
Tot-p38
P-ERK
Tot-ERK
β-actin
caspase-3
PARP-1

I

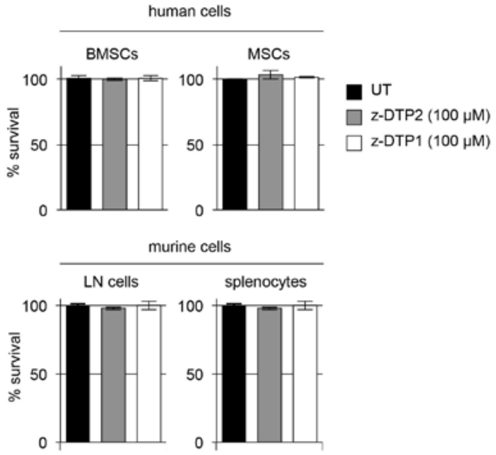
human cells
BMSCs
MSCs
murine cells
LN cells
splenocytes
% survival
100
50
0
UT
z-DTP2 (100 µM)
z-DTP1 (100 µM)

Figure S4, related to Figure 4. z-DTP1 and z-DTP2 exhibit high activity and stability, in vitro, and potent and selective capacity to kill MM cells by inducing JNK/MKK7-dependent apoptosis

(**A**) ELISA GADD45β/MKK7 competition assays showing the IC_{50} values of the Z-protected D-tetrapeptides, z-DTP1 and z-DTP2, before and after a 48-hr incubation with human serum at 37°C, as indicated. Values express the percentage of inhibition of GADD45β binding to MKK7 relative to the binding measured in the absence of peptide.

(**B**) Summary of the IC_{50} values of Ac-DTP1, Ac-DTP2, z-DTP1 and z-DTP2 in the ELISA competition assays shown in (**A**) and Figures 3A-B, in the presence or absence of a pre-incubation with serum (note the similar IC_{50} values given by each of the peptides before and after incubation with serum). Values denote means.

(**C**, **D**) [^{3}H]Thymidine incorporation assays showing the survival of representative MM cell lines after treatment with acetylated D-tetrapeptides (Ac-DTP1, Ac-DTP2, Ac-DNC; **C**) or Z-protected D-tetrapeptides (z-DTP1, z-DTP2, z-DNC; **D**) for the times indicated. (**C**) Ac-DNC, acetylated negative control D-tetrapeptide.

(**E**, **F**) [^{3}H]Thymidine incorporation assays showing the survival of representative sensitive (**E**) and resistant (KMM-1; **F**) MM cell lines after a 6-day treatment with the indicated concentrations of z-DTP1, z-DTP2 or z-DNC.

(**G**) PI nuclear staining assays showing apoptotic cells in representative MM cell lines that depend on GADD45β for survival after treatment with 10 μM of either z-DTP1, z-DTP2 or z-DNC for 6 days. The percentages of apoptotic cells are depicted. UT, untreated cells.

(**H**) Western blots showing total and phosphorylated (P) proteins in representative sensitive (blue) and resistant (red) MM cell lines after treatment with z-DTP2 (10 μM), z-DNC (10 μM), or TPA/ionomycin (T/I) for the times indicated. Filled and open arrowheads indicate the unprocessed forms and cleavage products, respectively, of caspase-3 and its proteolytic substrate, PARP-1. β-actin is shown as control.

(**I**) Shown is the survival of healthy, primary human bone marrow stromal cells (BMSCs), primary human mesenchymal stem cells (MSCs), and primary mouse splenocytes and lymph node (LN) cells after treatment with z-DTP1 or z-DTP2 for 72 hr (splenocytes, LN cells) or 144 hr (BMSCs, MSCs). Cell viability was measured using trypan blue exclusion assays (BMSCs) or [^{3}H]thymidine incorporation assays (MSCs, splenocytes, LN cells). Values express the percentage of live cells present in the

treated cultures relative to the live cells present in the respective untreated cultures, represented as 100%. UT, untreated.

(**A**, **C**, **D**, **E**, **F**, **I**) Values denote means ± SD (n=3).

(**A**, **D, E**, **F**, **G**) z-DNC, Z-protected negative control D-tetrapeptide.

(**C**, **D**, **E**, **F**) Values express the percentage of the cpm measured with the treated cultures relative to the cpm measured with the respective untreated cultures.

Table S3, related to Figure 4. List of the D-peptides used to improve cell penetration

amino acid sequence	MW	% inhibition (ELISA)	IC_{50} (ELISA)	IC_{50} (U266 cells)
z-YERF-NH_2 (z-DTP2)	746	95	0.19 nM	(*) 40.70 nM
z-YDHF-NH_2 (z-DTP1)	713	84	0.20 nM	(*) 40.70 nM
z-YDHQ-NH_2 (z-DNC)	694	12	> 10.00 nM	> 10,000.00 nM
z-YD(O-methyl)HF-NH_2	727	84	> 10.00 nM	N.D.
z-YD(O-methyl)HQ-NH_2	709	3	> 10.00 nM	N.D.
2Cl-z-YERF-NH_2	780	38	> 10.00 nM	N.D.
2Cl-z-YDHF-NH_2	747	38	> 10.00 nM	N.D.
2Cl-z-YDHQ-NH_2	729	40	> 10.00 nM	N.D.
Myr-YERF-NH_2	822	37	> 10.00 nM	> 10,000.00 nM
Myr-YDHF-NH_2	788	26	> 10.00 nM	> 10,000.00 nM
Myr-YDHQ-NH_2	770	28	> 10.00 nM	> 10,000.00 nM
Benzoyl-YERF-NH_2	717	24	> 10.00 nM	> 10,000.00 nM
Benzoyl-YDHF-NH_2	684	0	> 10.00 nM	> 10,000.00 nM
Benzoyl-YDHQ-NH_2	665	1	> 10.00 nM	> 10,000.00 nM
3-hydroxy-4-methoxy-benzyl-YERF-NH_2	755	86	< 10.00 nM	< 10,000.00 nM
3-hydroxy-4-methoxy-benzyl-YDHF-NH_2	722	81	< 10.00 nM	< 10,000.00 nM
3-hydroxy-4-methoxy-benzyl-YDHQ-NH_2	703	7	> 10.00 nM	> 10,000.00 nM
Fmoc-YERF-NH_2	834	60	< 10.00 nM	N.D.
Fmoc-YDHF-NH_2	802	58	< 10.00 nM	N.D.
Fmoc-YDFQ-NH_2	782	19	> 10.00 nM	N.D.
Ac-YERFK(εZ)-NH_2	916	6	> 10.00 nM	N.D.
Ac-YDHFK(εZ)-NH_2	883	8	> 10.00 nM	N.D.
Ac-YDHQK(εZ)-NH_2	865	2	> 10.00 nM	N.D.

Shown are the D-peptide derivatives designed to improve cell penetration and activity in myeloma cells. Also shown are the values obtained for each of these peptides in ELISA competition assays (reported both as percentage of GADD45β/MKK7 binding inhibition at the concentration of 10 nM and as IC_{50}), and in [^{3}H]thymidine incorporation or trypan blue exclusion assays in U266 myeloma cells (reported as IC_{50} at day 6. All peptides are in the D configuration). Z, benzyloxycarbonyl; 2Cl-Z, 2-chloro-benzyloxycarbonyl; Myr, myristyl; Fmoc, fluorenylmethoxycarbonyl; Ac, acetyl; MW, molecular weight; N.D., not determined. (*) Values are from Figure 4A.

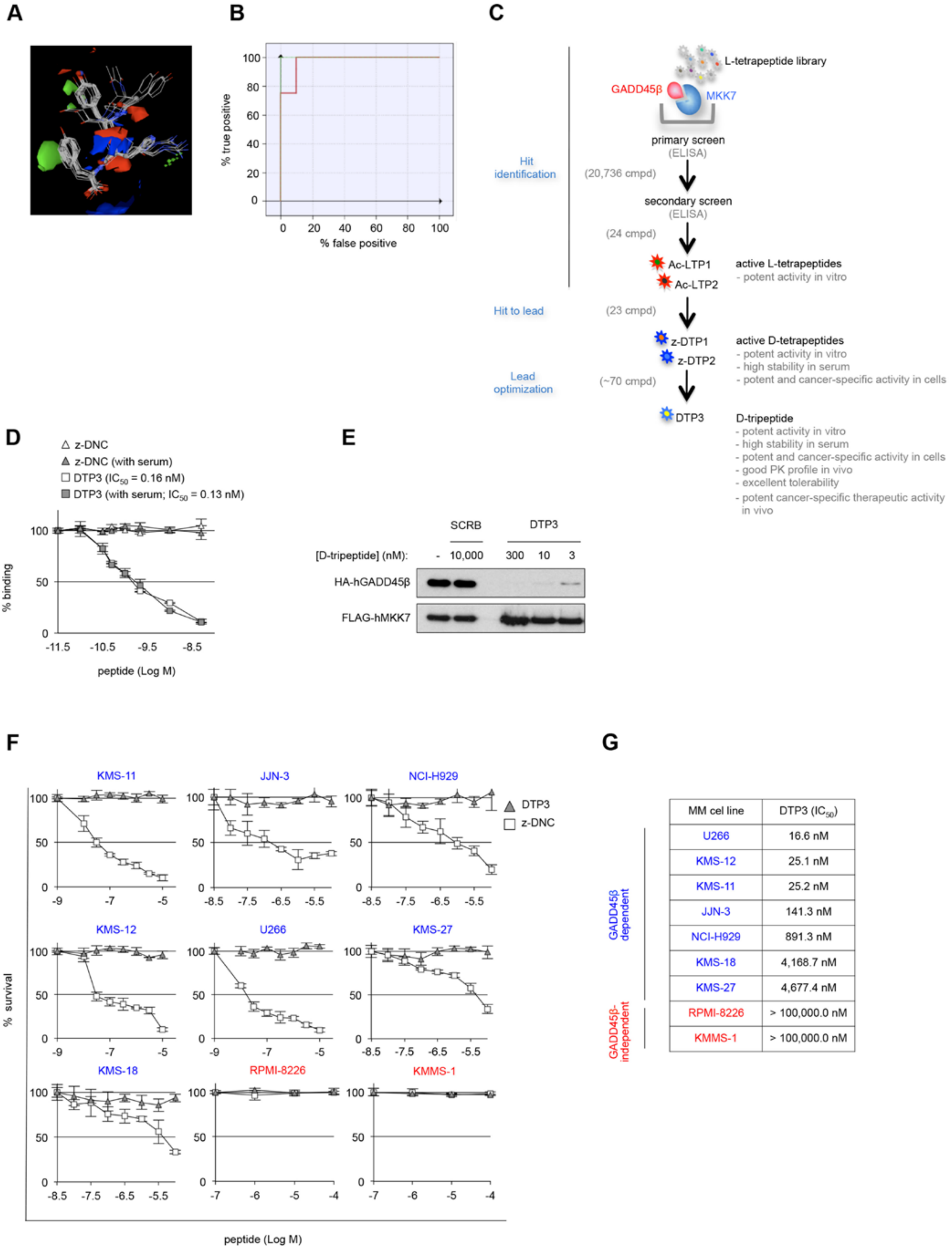

MM cel line	DTP3 (IC_{50})
U266	16.6 nM
KMS-12	25.1 nM
KMS-11	25.2 nM
JJN-3	141.3 nM
NCI-H929	891.3 nM
KMS-18	4,168.7 nM
KMS-27	4,677.4 nM
RPMI-8226	> 100,000.0 nM
KMMS-1	> 100,000.0 nM

H

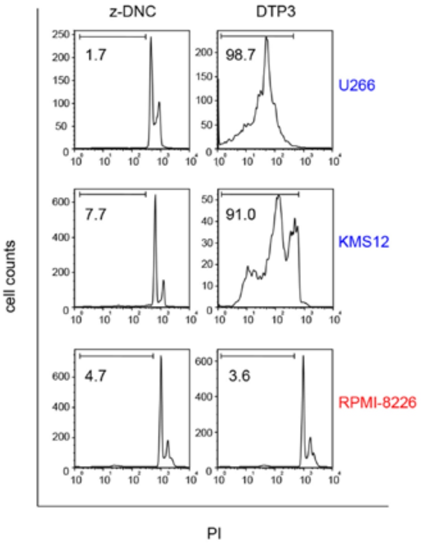
z-DNC
DTP3
1.7
98.7
U266
7.7
91.0
KMS12
4.7
3.6
RPMI-8226
cell counts
PI

I

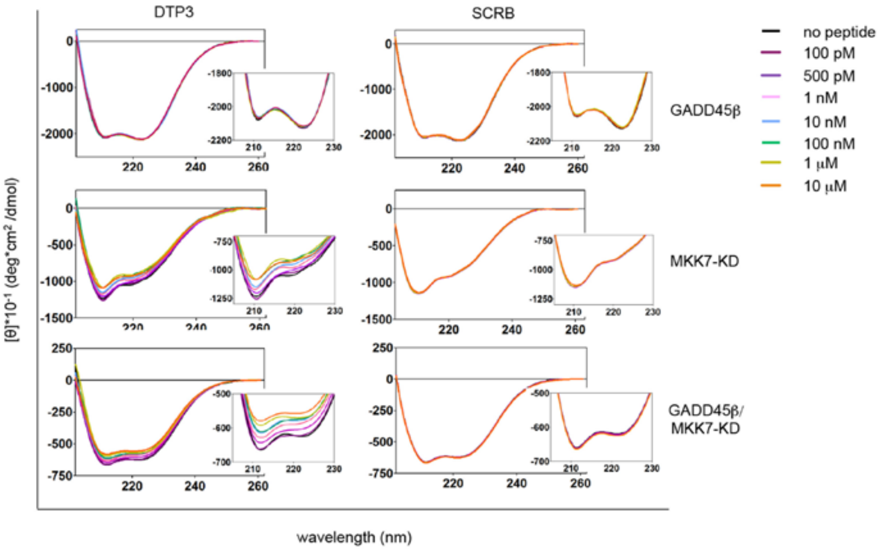
DTP3
SCRB
no peptide
100 pM
500 pM
1 nM
10 nM
100 nM
1 μM
10 μM
GADD45β
MKK7-KD
GADD45β/
MKK7-KD
[θ]*10⁻¹ (deg*cm² /dmol)
wavelength (nm)

J

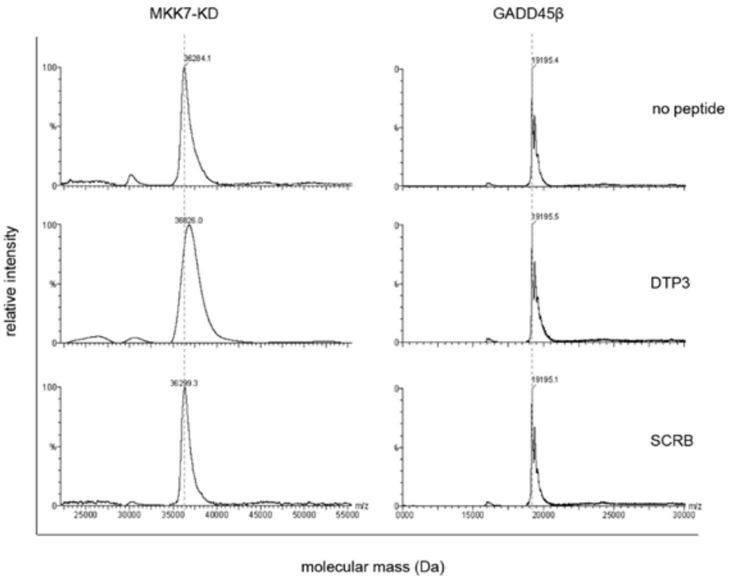
MKK7-KD
GADD45β
no peptide
DTP3
SCRB
relative intensity
molecular mass (Da)

K

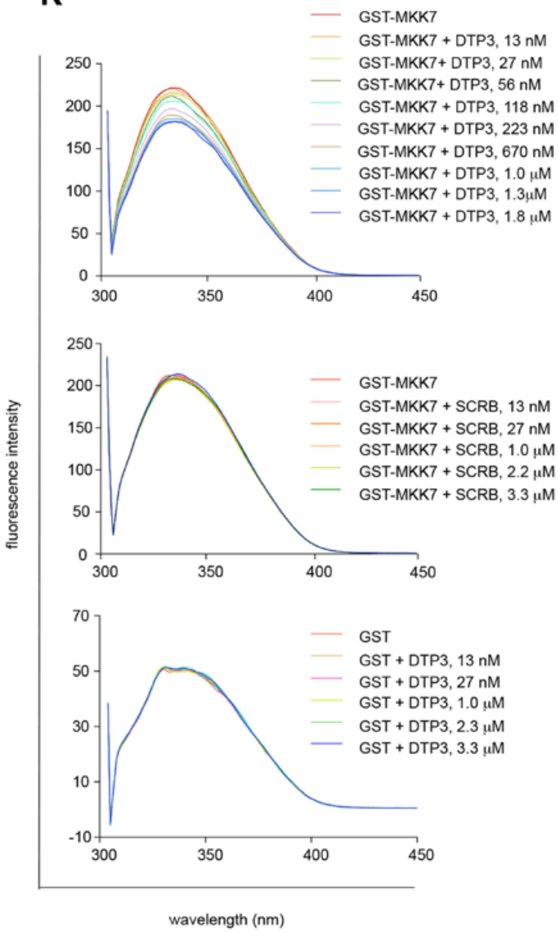
GST-MKK7
GST-MKK7 + DTP3, 13 nM
GST-MKK7+ DTP3, 27 nM
GST-MKK7+ DTP3, 56 nM
GST-MKK7 + DTP3, 118 nM
GST-MKK7 + DTP3, 223 nM
GST-MKK7 + DTP3, 670 nM
GST-MKK7 + DTP3, 1.0 μM
GST-MKK7 + DTP3, 1.3μM
GST-MKK7 + DTP3, 1.8 μM
GST-MKK7
GST-MKK7 + SCRB, 13 nM
GST-MKK7 + SCRB, 27 nM
GST-MKK7 + SCRB, 1.0 μM
GST-MKK7 + SCRB, 2.2 μM
GST-MKK7 + SCRB, 3.3 μM
GST
GST + DTP3, 13 nM
GST + DTP3, 27 nM
GST + DTP3, 1.0 μM
GST + DTP3, 2.3 μM
GST + DTP3, 3.3 μM
fluorescence intensity
wavelength (nm)

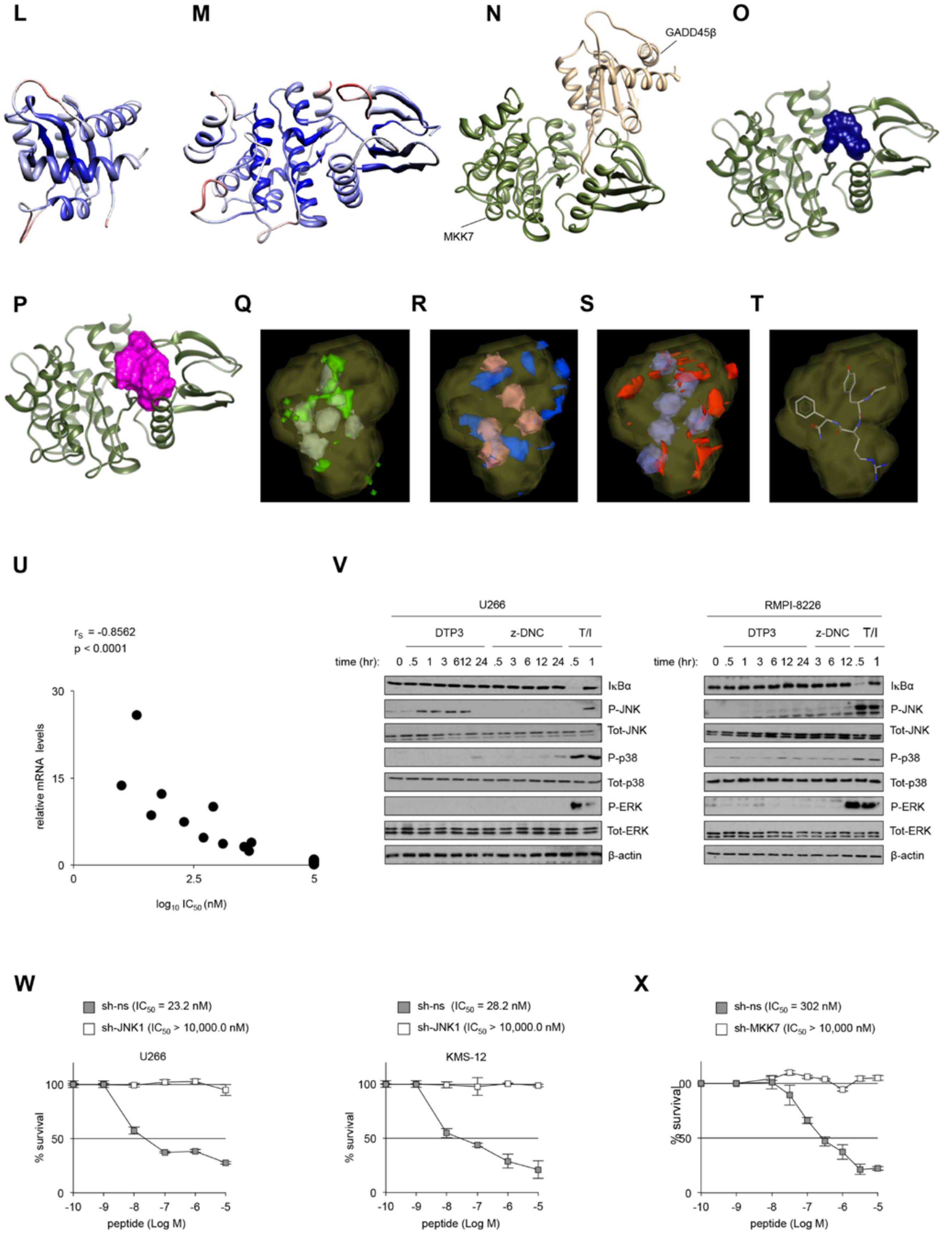

L
M
N
O
GADD45β
MKK7
P
Q
R
S
T
U
r_S = -0.8562
p < 0.0001
relative mRNA levels
30
15
0
2.5
5
log_{10} IC_{50} (nM)
V
U266
DTP3
z-DNC
T/I
time (hr): 0 .5 1 3 6 12 24 .5 3 6 12 24 .5 1
RMPI-8226
DTP3
z-DNC
T/I
time (hr): 0 .5 1 3 6 12 24 3 6 12 .5 1
IκBα
P-JNK
Tot-JNK
P-p38
Tot-p38
P-ERK
Tot-ERK
β-actin
W
sh-ns (IC_{50} = 23.2 nM)
sh-JNK1 (IC_{50} > 10,000.0 nM)
U266
sh-ns (IC_{50} = 28.2 nM)
sh-JNK1 (IC_{50} > 10,000.0 nM)
KMS-12
X
sh-ns (IC_{50} = 302 nM)
sh-MKK7 (IC_{50} > 10,000 nM)
% survival
100
50
0
-10
-9
-8
-7
-6
-5
peptide (Log M)

Figure S5, related to Figure 5. The development of DTP3: a potent and cancer-selective GADD45β/MKK7 inhibitor with improved bioavailability in vivo

(**A**) Shown is the putative bioactive pharmacophore model derived by FLAP (Baroni et al., 2007; Kozakov et al., 2001) using the structural superposition of 12 bioactive peptides (i.e. peptides with IC_{50} ≤10 nM in ELISA competition assays). Regions matching hydrophobic, hydrogen bond acceptor, and hydrogen bond donor features of the peptides are represented by green, red, and blue contours, respectively.

(**B**) Graph showing the discrimination capacity of the pharmacophore model for an additional set of active (bottom) and inactive peptides that were not used in (**A**) to generate this pharmacophore model. The plot shows the percentage of true positives (i.e. peptides inhibiting the GADD45β/MKK7 interaction by more than 60% at 42 nM in ELISA competition assays) as a function of the percentage of false positives (i.e. peptides inhibiting the GADD45β/MKK7 interaction by less than 30% at 42 nM in ELISA competition assays). The area under the curve for the plot is 0.92.

(**C**) Summary of the drug discovery strategy that led to the development of DTP3. cmpd, compounds.

(**D**) ELISA GADD45β/MKK7 competition assays showing the IC_{50} values of DTP3 and the scrambled control D-tripeptide, SCRB, before and after a 48-hr pre-incubation with human serum, at 37°C, as indicated. Values express the percentage of inhibition of GADD45β binding to MKK7 relative to the binding measured in the absence of peptide.

(**E**) Co-immunoprecipitation assays showing the disruption of the GADD45β/MMK7 complex by DTP3, but not by the SCRB control D-peptide, at the indicated concentrations. Co-immunoprecipitations (IP) were performed using an anti-FLAG antibody, as in Figure 3C; western blots were developed using an anti-HA or an anti-MKK7 antibody, as shown. -, incubation without D-tripeptide.

(**F**) [^{3}H]Thymidine incorporation assays showing the survival of GADD45β-dependent (blue) and GADD45β-independent (red) MM cell lines after a 6-day treatment with the indicated concentrations of DTP3 or a negative control D-peptide (z-DNC).

(**G**) Shown are IC_{50} values of DTP3 at 144 hr for the experiment reported in (**F**).

(**H**) PI nuclear staining assays showing apoptotic cells in representative GADD45β-dependent and GADD45β-independent MM cell lines from (**F**, **G**) after a 6-day treatment with 10 μM of either DTP3 or z-DNC. The percentages of apoptotic cells are depicted.

(**I**) Circular dichroism (CD) analyses showing the effects of DTP3 (left) and the SCRB peptide (right) at increasing concentrations, ranging from 0.1 nM to 10 μM, on the conformation of GADD45β (1.25 μM; top), the kinase domain (KD) of MKK7 (MKK7-KD, 1.25 μM; middle), and the GADD45β/MKK7-KD complex (1.25 μM of each protein; bottom). Insets depict magnifications of the CD spectra of the proteins within the 200-230 nm UV region (note the reduction in the intensity of the spectra of MKK7 [middle, left] and the complex [bottom, left] at the wavelengths of approximately 210 and 220 nm, denoting a conformational rearrangement of the proteins, in the presence of DTP3, but not of the SCRB D-peptide).

(**J**) MALDI-TOF mass spectrometry (MS) analysis showing the effects of DTP3 (middle) and the SCRB D-peptide (bottom) on the mass peak values of MKK7-KD (left) and GADD45β (right), as indicated. Proteins were incubated at the concentration of 5 μM in the presence or absence of an equimolar concentration (5 μM) of D-tripeptide, as shown. The experimental molecular weight (MW) of each of the proteins was in close agreement with that expected on the basis of the primary structures, which predict a MW of 19,195 Da for GADD45β and of 36,283 Da for MKK7-KD. Shown is the shift induced in the mass peak value of MKK7-KD (top, left) in the presence of DTP3 (middle, left), but not of the SCRB peptide (bottom, left), denoting the binding of this protein to the active D-tripeptide.

(**K**) Tryptophan fluorescence quenching analysis showing the effects of DTP3 (top, bottom) and the SCRB D-peptide (middle) on the fluorescence emission spectra of GST-hMKK7 (top, middle) and GST (bottom) (note the 2-nm blue shift of the fluorescence maximum of the emission spectrum of GST-MKK7 at the wavelength of 332-334 nm in the presence of DTP3 [top], but not of the SCRB D-peptide [middle]). GST-hMKK7 was used at the concentration of 1.25 μM and incubated in the presence or absence of increasing concentrations of DTP3 (top) or SCRB D-peptide (middle), ranging from 13 nM to 3.3 μM, as indicated. GST (1.25 μM) (bottom) was used as control. The dose-response curve of the -Δfluorescence values of GST-hMKK7 at the wavelength of 333 nm plotted against the concentration values of DTP3 for the experiment in the top panel is shown in Figure 5B. The stoichiometry of the peptide interaction with the protein on the basis of the values reported in the top panel was 1:1. The estimated equilibrium dissociation constant (K_D) value of this interaction was 64.81 ± 6.22 nM (Williamson, 2013).

(**L**) Shown is the model of GADD45β built by homology, using as template the crystal structure of human GADD45γ (PDB Id: 3FFM), which shares 58% amino acid sequence identity with GADD45β.

(**M**) The coordinates of MKK7 were rebuilt on the basis of the experimental structure factors deposited in the PDB database (PDB Id: 2DYL). The missing residues were modeled.

(**L**, **M**) Proteins are coloured according to the estimated residue error (Benkert et al., 2011), using a gradient ranging from blue (most reliable regions) to red (least reliable regions).

(**N**) The model of the interaction between GADD45β and MKK7 was obtained from the coordinates of the models shown in (**L**) and (**M**), using a rigid body docking approach and refining the resulting best model in explicit solvent. The final model of the complex is shown. Most of the protein-protein interactions involve the α4-β4 loop (residues 104-117) and β2-α3 connecting region (residues 63-67) of GADD45β and the region of MKK7 including the ATP-binding site and the basic residue-rich region, comprising β2 and β3, previously identified as the GADD45β-binding site (Papa et al., 2004; Papa et al., 2007). As shown, GADD45β occupies most of the cleft between the two lobes of MKK7 hosting the ATP-binding site, consistent with the mechanism by which GADD45β blocks the catalytic activity of the kinase (Papa et al., 2004; Papa et al., 2007). Green, MKK7; beige, GADD45β.

(**L**, **M**, **N**) The models of GADD45β (**L**), MKK7 (**M**), and the GADD45β/MKK7 complex (**N**) are shown in ribbon representation.

(**O**, **P**) Shown are the top scoring ligand-binding sites identified by FTSite (**O**) and FLAP (**P**) on MKK7 (depicted in blue and pink, respectively). As illustrated, these sites are located within the same region of the protein, near the predicted interface of the kinase with GADD45β within the complex. The orientation of MKK7 is the same as in (**M**).

(**Q**, **R**, **S**) Superposition of the molecular interaction fields (MIFs) of the putative binding site of MKK7 and the pharmacophore model as computed by FLAP, using an hydrophobic/hydrophobic (**Q**), H-bond donor/acceptor (**R**) and H-bond acceptor/donor (**S**) probe. Hydrophobic areas are shown in green; H-bond acceptor and donor areas are shown in red and blue, respectively. Lighter colours denote the pharmacophore model; darker ones denote the ligand-binding site of MKK7. The region of MKK7 identified as favourable for interacting with the pharmacophore model is shown with a brown contour (note the complementarities of the pharmacophore model with the cognate energy fields of the predicted binding site of MKK7). The physicochemical properties of the pharmacophore model and the putative ligand-binding pocket of the kinase are complementary in the areas in which there is a match between hydrophobic regions ([**Q**], dark and light green), and donors and acceptors of H bonds ([**R**, **S**], blue and red).

(**T**) For clarity purposes, the predicted orientation of the structure of DTP3 is shown in place of the pharmacophore model within the context of the putative binding region of MKK7, depicted as in (**Q**, **R**, **S**).

(**U**) Shown is the correlation plot of the relative *GADD45B* mRNA expression (qRT-PCR assays) and the $\log_{10}$ IC_{50}s of z-DTP2 at 144 hr ([^{3}H]thymidine incorporation assays) in a panel of cancer cell lines of different tissues of origin. r_S, Spearman correlation coefficient (r_S=-0.8562; p<0.0001). Similar results were obtained with z-DTP1 (data not shown).

(**V**) Western blots showing total and phosphorylated (P) proteins in representative sensitive (U266) and resistant (RPMI-8226) myeloma cell lines after treatment with DTP3 (10 μM), z-DNC (10 μM) or TPA/ionomycin (T/I) for the times indicated. β-actin is shown as control.

(**W**) [^{3}H]Thymidine incorporation assays showing the survival of sensitive U266 and KMS-12 MM cells expressing sh-ns or sh-JNK1 after a 6-day treatment with increasing concentrations of DTP3. Also depicted are the IC_{50} values of DTP3 in sh-ns- and sh-JNK1-expressing cells.

(**X**) [^{3}H]Thymidine incorporation assays showing the survival of ARH-77 B lymphoblastoid cells expressing sh-ns or sh-MKK7 sh-RNAs after a 72-hr treatment with the indicated concentrations of z-DTP2. The IC_{50} values of z-DTP2 in sh-ns- and sh-MKK7-expressing cells are also depicted. Similar results were obtained with z-DTP1 (data not shown).

(**D**, **F**, **W**, **X**) Values denote means ± SD (n=3).

(**F**, **W**, **X**) Values express the percentage of the cpm measured with the treated cultures relative to the cpm measured with the respective untreated cultures.

Table S4, related to Figure 5. List of the peptides used to generate and validate the pharmacophore model

amino acid sequence	IC_{50}s (ELISA)	% inhibition of (ELISA)	
Ac-YQRF-NH_2	5.00 nM	N.D.	active
Ac-YNRF-NH_2	10.00 nM	N.D.	
Ac-YLRF-NH_2	5.00 nM	N.D.	
Ac-YLF-NH_2	1.80 nM	N.D.	
Ac-YNF-NH_2	1.90 nM	N.D.	
Ac-YPF-NH_2	0.65 nM	N.D.	
Ac-YHF-NH_2	0.70 nM	N.D.	
H-YHF-NH_2	0.65 nM	N.D.	
Ac-YRY-NH_2	0.76 nM	N.D.	
H-YRY-NH_2	0.75 nM	N.D.	
Ac-YKF-NH_2	0.85 nM	N.D.	
Ac-YRF-NH_2 (DTP3)	0.16 nM	(#) 94	active
Ac-YERF-NH_2 (Ac-LTP2)	(*) 0.40 nM	(#) 93	
Ac-WDRW-NH_2	(*) < 42.00 nM	(#) 93	
Ac-WEHW-NH_2	(*) < 42.00 nM	(#) 75	
Ac-WERW-NH_2	(*) < 42.00 nM	(#) 69	
Ac-WDKW-NH_2	(*) < 42.00 nM	(#) 69	
Ac-WEKF-NH_2	(*) < 42.00 nM	(#) 65	
Ac-WEKW-NH_2	(*) < 42.00 nM	(#) 58	
Ac-YEKF-NH_2	(*) < 42.00 nM	(#) 64	
Ac-YEHW-NH_2	(*) < 42.00 nM	(#) 64	
Ac-WERF-NH_2	(*) > 42.00 nM	(#) 28	inactive
Ac-YERA-NH_2	> 10.00 nM	(##) 28	
Ac-WDHF-NH_2	(*) > 42.00 nM	(#) 28	
Ac-YEKW-NH_2	(*) > 42.00 nM	(#) 22	
Ac-WDKF-NH_2	(*) > 42.00 nM	(#) 25	
Ac-PERF-NH_2	> 10.00 nM	(##) 24	
Benzoyl-YERF-NH_2	(**) > 10.00 nM	(##) 24	
Ac-YDKW-NH_2	> 10.00 nM	(#) 43	
Ac-YARF-NH_2	> 10.00 nM	(#) 17	
Ac-YEAF-NH_2	> 10.00 nM	(#) 13	
Ac-YPRF-NH2	> 10.00 nM	(#) 13	
Ac-YEPF-NH_2	> 10.00 nM	(#) 13	
Ac-YERP-NH_2	> 10.00 nM	(#) 0	
Ac-AERF-NH_2	> 10.00 nM	(#) 7	
Benzoyl-YDHF-NH_2	(**) > 10.00 nM	(##) 1	
Benzoyl-YDHQ-NH_2	(**) > 10.00 nM	(##) 0	

Shown are the 12 bioactive peptides used in Figure S5A to derive the pharmacophore model (active [top]). Also shown are the 26 active (bottom) and inactive peptides used in Figure S5B to assess the pharmacophore classification ability. Reported are the percentages of GADD45β/MKK7 binding inhibition measured at the peptide concentration of either 10 or 42 nM, as shown, and the IC_{50} values obtained for each of the peptides in ELISA competition assays. Values in the right column express the percentages of inhibition of GADD45β binding to MKK7 obtained with each peptide at the indicated concentration relative to the binding measured in the absence of peptide and denote means. Amino

acids are identified by the standard single-letter amino acid code. N.D., not determined. * Values are from Table S2. ** Values are from Table S3. (#) Peptide tested at a concentration of 42.00 nM. (##) Peptide tested at a concentration of 10.00 nM.

Table S5, related to Figure 5. Broad kinase-panel profiling showing the absence of any significant effect of DTP3 on the activity of 142 human kinases

	% kinase activity with DTP3					% kinase activity with DTP3					% kinase activity with DTP3			
	at 1 μM		at 10 μM			at 1 μM		at 10 μM			at 1 μM		at 10 μM	
kinase ID	mean	SD	mean	SD	kinase ID	mean	SD	mean	SD	kinase ID	mean	SD	mean	SD
MKK7	104	8	91	6	BRSK1	102	5	102	5	TGFBR1	107	5	102	10
MKK1	91	3	96	0	BRSK2	88	8	101	13	Src	100	0	96	7
MKK2	111	4	110	9	MELK	100	2	99	13	Lck	108	16	109	9
MKK6	97	7	104	7	NUAK1	91	3	99	4	CSK	90	5	113	32
ERK1	103	0	93	11	SIK2	95	2	95	5	YES1	91	13	83	9
ERK2	102	3	96	9	SIK3	98	11	117	1	ABL	96	7	93	4
ERK5	105	2	90	3	TSSK1	104	7	106	5	BTK	101	6	103	4
JNK1	107	20	100	3	CK1γ2	82	8	72	3	JAK2	90	4	102	8
JNK2	106	2	99	1	CK1δ	95	4	92	11	SYK	75	0	78	5
JNK3	98	12	90	19	CK2	96	6	103	7	ZAP70	97	21	90	2
p38α MAPK	83	17	83	8	TTBK1	85	2	77	7	TIE2	86	15	102	9
p38β MAPK	115	4	109	14	TTBK2	101	1	102	1	BRK	124	15	94	7
p38γ MAPK	97	15	109	2	DYRK1A	98	3	108	14	EPH-A2	97	2	94	5
p38δ MAPK	97	2	103	1	DYRK2	94	3	92	21	EPH-A4	94	28	89	0
ERK8	89	13	85	16	DYRK3	95	8	100	13	EPH-B1	100	17	92	12
RSK1	97	9	110	5	NEK2a	111	13	102	1	EPH-B2	98	19	83	8
RSK2	103	12	91	12	NEK6	86	3	112	29	EPH-B3	95	5	101	17
PDK1	111	5	103	2	IKKβ	92	0	96	4	EPH-B4	91	3	90	4
PKBα	88	4	90	1	IKKε	97	13	92	11	FGF-R1	79	2	89	10
PKBβ	105	5	77	33	TBK1	99	3	101	5	HER4	95	2	95	10
SGK1	96	2	92	1	PIM1	91	0	90	7	IGF-1R	95	15	99	3
S6K1	98	2	93	8	PIM2	94	15	110	10	IR	87	24	96	18
PKA	92	4	99	2	PIM3	107	3	99	1	IRR	99	1	102	1
ROCK 2	115	20	74	30	SRPK1	98	2	96	3	TrkA	102	5	110	3
PRK2	90	11	92	0	EF2K	97	6	93	8	DDR2	93	1	88	9
PKCα	103	0	102	4	EIF2AK3	104	10	117	13	VEG-FR	85	1	78	8
PKCγ	105	6	114	5	HIPK1	105	2	109	4	PDGFRA	89	3	95	3
PKCζ	101	3	105	7	HIPK2	108	6	101	5	PINK	103	6	95	6
PKD1	94	13	89	11	HIPK3	103	5	84	2					
STK33	103	1	104	0	CLK2	122	28	97	12					
MSK1	94	0	102	0	PAK2	102	13	106	3					
MNK1	99	20	93	0	PAK4	97	13	98	12					
MNK2	119	7	130	27	PAK5	82	10	85	7					
MAPKAP-K2	94	1	106	6	PAK6	95	1	93	7					
MAPKAP-K3	104	1	100	4	MST2	103	6	106	2					
PRAK	96	9	102	18	MST3	123	2	121	17					
CAMKKβ	107	30	110	5	MST4	114	13	116	12					
CAMK1	108	12	96	3	GCK	102	13	93	7					
SmMLCK	105	3	97	2	MAP4K3	79	3	94	18					
PHK	118	15	109	7	MAP4K5	99	8	90	14					
DAPK1	107	6	100	9	MINK1	109	11	115	16					
CHK1	107	4	88	23	MEKK1	89	1	97	6					
CHK2	99	6	100	0	MLK1	99	3	105	7					
GSK3β	95	0	94	2	MLK3	90	19	87	5					
CDK2-Cyclin A	94	7	105	3	TESK1	103	15	88	1					
CDK9-Cyclin T1	96	3	107	2	TAO1	85	7	94	8					
PLK1	101	6	95	15	ASK1	106	12	93	1					
Aurora A	105	3	101	7	TAK1	100	4	109	1					
Aurora B	100	2	113	1	IRAK1	95	1	99	5					
TLK1	103	11	93	12	IRAK4	104	2	105	6					
LKB1	104	8	102	3	RIPK2	100	4	104	8					
AMPK	92	19	103	4	OSR1	112	7	102	4					
AMPK (hum)	108	10	116	2	TTK	96	5	100	3					
MARK1	123	0	110	16	MPSK1	102	14	108	4					
MARK2	109	3	96	13	WNK1	104	15	100	1					
MARK3	124	3	108	3	ULK1	103	2	95	2					
MARK4	107	4	100	1	ULK2	83	15	88	7					

DPT3 was tested in kinase assays at the concentration of either 1 or 10 μM, as indicated, at the International Centre for Kinase Profiling, MRC-Protein Phosphorylation Unit, Dundee (http://www.kinase-screen.mrc.ac.uk/). The kinase ID names are shown. Values express the percentage of kinase activity measured in the presence of DTP3 relative to the kinase activity measured in the absence of peptide and denote means ± SD (n=3).

A

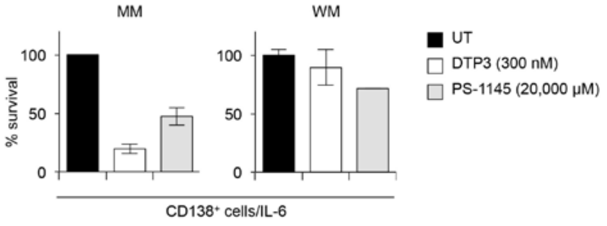

B

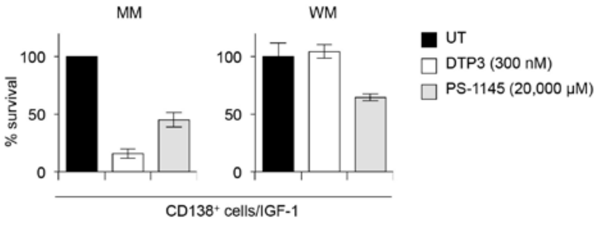

C

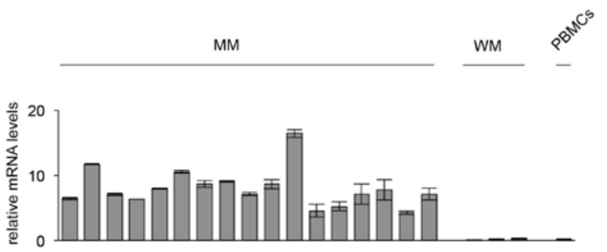

D

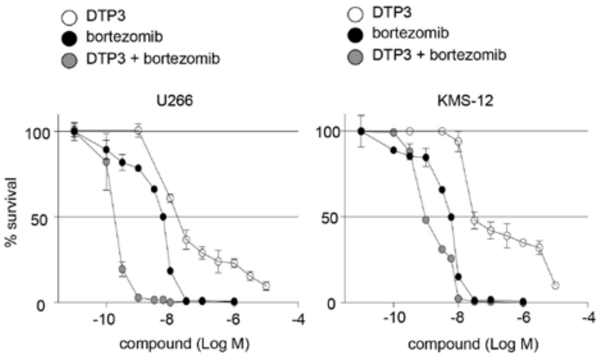

E

MM cell line	Fraction Affected (FA)	DTP3 (Concentration)		bortezomib (Concentration)		Combination Index (CI)	Description
		with bortezomib	alone	with DTP3	alone		
U266	0.5	3 nM	16.6 nM	0.19 nM	6 nM	0.21	strong synegism
KMS-12	0.5	10 nM	25.1 nM	1 nM	6 nM	0.56	synegism

F

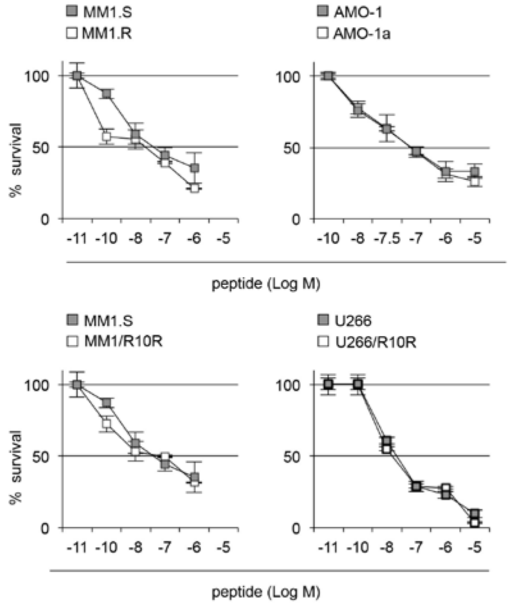

G

Drug	MM cell line	DTP3 (IC_{50})
Dexamethasone	MM1.S (parental)	457.1 nM
	MM1.R (resistant)	240.0 nM
Bortezomib	AMO-1 (parental)	87.1 nM
	AMO-1a (resistant)	77.6 nM
Lenalidomide	U266 (parental)	16.6 nM
	U266/R10R (resistant)	16.2 nM
	MM1.S (parental)	457.1 nM
	MM1/R10R (resistant)	660.7 nM

Figure S6, related to Figure 6. DTP3 exhibits more potent and selective activity than IKKβ inhibitors, synergizes with bortezomib, and kills drug-resistant MM cells

(**A**, **B**) Trypan blue exclusion assays showing the survival of primary $CD138^+$ cells from MM or Waldenström's macroglobulinemia (WM) patients after treatment with DTP3 (300 nM) or PS-1145 (20 μM) for 48 hr, in the presence of IL-6 (10 ng/mL; **A**) or IGF-1 (50 ng/mL; **B**) stimulation. Values express the percentage of live cells present in the treated cultures relative to the live cells present in the respective untreated cultures and denote means ± SD (WM; n=3) or SEM (MM; n=8). UT, untreated.

(**C**) qRT-PCR assays showing the relative mRNA expression of *GADD45B* in $CD138^+$ cells from MM patients, $CD138^+$ cells from WM patients, and healthy human PBMCs. Values represent individual patients and denote means ± SD (n=3).

(**D**) [^{3}H]Thymidine incorporation assays showing the survival of representative GADD45β-dependent MM cell lines after treatment with the indicated concentrations of DTP3 and bortezomib, used either as single agents or in combination. Treatments with DTP3 were for 6 days; bortezomib was added to the cell cultures 48 hr prior to the measurement of cell viability. For the combination treatment, DTP3 was used at the concentrations of 3 nM in U266 cells and of 10 nM in KMS-12 cells, whereas bortezomib was used at increasing concentrations, as shown. Based on the data shown, the IC_{50} value of bortezomib as single agent at 48 hr was 6 nM in each of the two MM cell lines. The survival curves of U266 and KMS-12 cells following treatment with DTP3 as single agent are from the experiment shown in Figure S5F.

(**E**) Shown is the combination index (CI) of DTP3 and bortezomib for the experiment shown in (**D**). Viability data from (**D**) were converted into values representing the fraction of cells affected (FA) equaling 0.5 in the drug-treated cultures compared with untreated cultures, and the interaction of DTP3 with bortezomib was analyzed according to the Chou-Talalay method (Chou, 2006). Also shown are the drug concentrations giving the FA value of 0.5 and the description of the CI. Based on the Chou-Talalay equation (Chou, 2006), synergy is present when the CI is less than 1.0 (where values between 0.3 and 0.7 denote synergism, and values between 0.1 and 0.3 denote strong synergism); the combination is additive when CI equals 1.0, and antagonistic when it is more than 1.0 (Chou, 2006).

(**F**) [^{3}H]Thymidine incorporation assays showing the survival of sensitive and drug-resistant MM cell lines following a 6-day treatment with the indicated concentrations of DTP3.

(**G**) Shown are IC_{50} values of DTP3 for the experiment in (**F**).

(**D**, **F**) Values express the percentage of the cpm measured with the treated cultures relative to the cpm measured with the respective untreated cultures and denote means ± SD (n=3).

(**F**, **G**) Matching pairs of sensitive (parental) and drug-resistant MM cell lines were as follows: MM1.S (parental) and MM1.R (dexamethasone-resistant); AMO-1 (parental) and AMO-1a (bortezomib-resistant); MM1.S (parental) and MM1/R10R (lenalidomide-resistant); U266 (parental) and U266/R10R (lenalidomide-resistant). Data with the parental U266 MM cell line were from the experiment shown in Figures S5F-G (5 out 7 DTP3 concentrations only). For clarity purposes, the same data for the parental MM1.S MM cell line are shown as control for the dexamethasone-resistant (MM1.R) MM cell line and the lenalidomide-resistant (MM1/R10R) MM cell line.

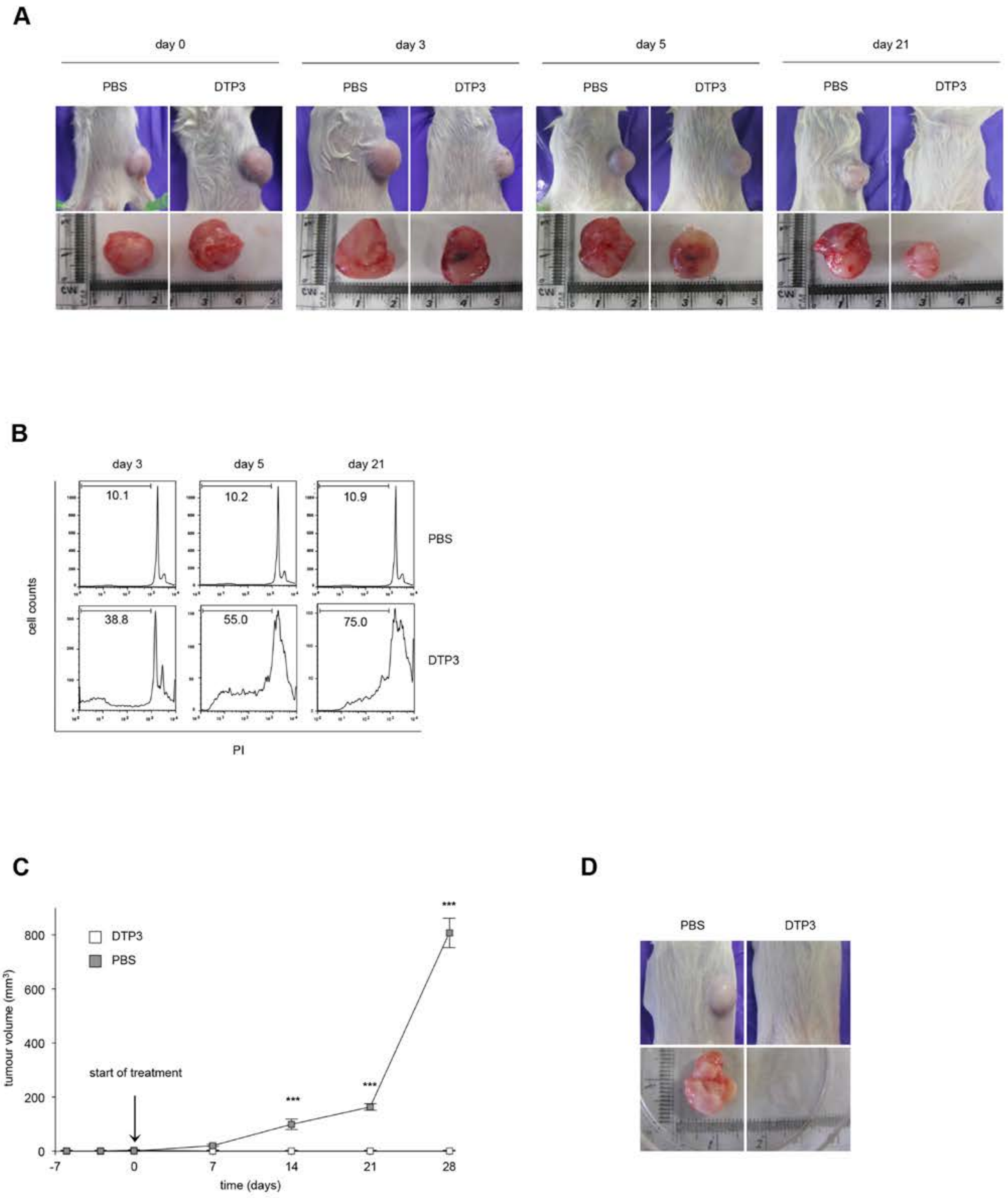
A
day 0
day 3
day 5
day 21
PBS
DTP3
B
day 3
day 5
day 21
10.1
10.2
10.9
PBS
38.8
55.0
75.0
DTP3
cell counts
PI
C
DTP3
PBS
tumour volume (mm3)
start of treatment
***
time (days)
D
PBS
DTP3

Figure S7, related to Figure 7. Treatment with DTP3 potently induces apoptosis in myeloma xenografts, in vivo, at doses that produce no apparent side effects

(**A**) Images of representative myeloma-bearing mice (top) and isolated U266 tumors (bottom) at different times after administration of DTP3 at the dose of 14.5 mg/kg/day, yielding a steady-state peptide plasma concentration of approximately 0.7 μM, or PBS.

(**B**) PI nuclear staining assays showing apoptotic cells in the tumor tissues of representative myeloma-bearing mice from (**A**). The percentages of apoptotic cells are depicted.

(**C**) Shown are the volumes of subcutaneous KMS-11 myeloma xenografts in mice treated by continual infusion of DTP3 at the dose of 14.5 mg/kg/day or PBS, as in Figures 7B-E, for the times indicated. Values denote means ± SEM (n=4). ***, $p<0.001$.

(**D**) Images of representative myeloma-bearing mice (top) and isolated tumors (bottom) from (**C**) at day 28 of the start of treatment.

Table S6, related to Figure 7. Summary of the pharmacokinetic profiles of DTP3 and z-DTP2

PK Paramenters		Values	Drug
In vitro	MW	525.38 Da	DTP3
	LogD	1.19 ± 0.29 (SD)	
	plasma stability (human)	> 48hrs	
	PPB (fraction unbound)	0.42 ± 0.01 (SD) (mouse)<br>0.933 ± 0.004 (SD) (human)	
	microsomal stability (human)	4.87 ± 1.24 µL/min/mg protein<br>$t_{½}$ = 285 min (SEM)	
	thermodynamic solubility (in PBS, pH 7.4, for 14 days at 37 ^{o}C)	169 ± 7.53 mg/ml (SD)	
In vivo	Toxicity - single i.v. (10 mg/kg)	no apparent toxicity	
	Toxicity - single s.c. (10 mg/kg)	no apparent toxicity	
	Toxicity - oral (100 mg/kg)	no apparent toxicity	
	Toxicity - continual s.c. dosing (14.5 mg/kg/day for 28 days)	no apparent toxicity	
	$t_{½}$	2.09 ± 0.07 hrs (SD)	z-DTP2
	CL	78.11 ± 11.67 mL/min/kg (SD)	
	V_d	14.58 ± 2.23 L/kg (SD)	
	AUC	2.062 ± 0.25 µg·hr/mL (SD)	

Shown are the values of the main in vitro pharmacokinetic (PK) parameters of DTP3, including distribution coefficient (LogD), plasma stability, plasma protein binding (PPB), microsomal stability, and thermodynamic solubility. Also shown is the excellent tolerability of DTP3 in mice after either a single intravenous (i.v.) injection, a subcutaneous (s.c.) injection, or a per os administration at the doses indicated. Additionally, shown are the excellent tolerability and lack of any apparent side effects of DTP3 after prolonged administration via osmotic pumps over a period of 28 days at the therapeutic dose of 14.5 mg/kg/day. Reported at the bottom are the values of the main in-vivo pharmacokinetic parameters of z-DTP2 after a single intravenous injection at the dose of 10 mg/kg, including terminal half-life (t1/2), plasma clearance (CL), volume of distribution (Vd), and area under the plasma concentration versus time curve (AUC). As also shown in Figure 7A, DTP3 exhibited somewhat shorter t1/2, but significantly lower CL and much lower Vd values than z-DTP2. As a result of these properties, the predicted doses of DTP3 and z-DTP2 required to achieve the therapeutic plasma concentration of 1 µM at the steady state, on the basis of the measured pharmacokinetic values and using the equation described in the Supplemental Experimental Procedures, were 0.81 mg/kg/hr and 3.61 mg/kg/hr, respectively, thus demonstrating the superior pharmacokinetic profile of DTP3 as compared with z-DTP2. Values denote means ± SD (n=3) or SEM (n=5), as indicated. MW, molecular weight.

SUPPLEMENTAL EXPERIMENTAL PROCEDURES

Antibodies, Reagents and Western Blots

Western blots were performed as described previously (Papa et al., 2008; Mauro et al., 2011). The antibodies used were: anti-IκBα (sc-1643; 1:5,000), anti-PARP-1 (sc-56197; 1:1,000), anti-β-actin (sc-1616; 1:3,000), anti-MEK-7 (sc-7104; 1:1,000), anti-HA (sc-805; 1:1,000), goat anti-rabbit IgG-HRP (sc-2030; 1:2,500), donkey anti-goat IgG-HRP (sc-2020; 1:5,000), goat anti-mouse IgG-HRP (sc-2005; 1:2,500), anti-GADD45β (sc-133606; 1:500) (Santa Cruz Biotechnology); anti-PARP-1 (5625P; cleaved product; 1:1,000), anti-caspase-3 (9662S; 1:1,000), anti-JNK (9258S 1:1,000), anti-P-JNK (9251S 1:1,000), anti-p38 (9212; 1:1,000), anti-P-p38 (9215S; 1:1,000), anti-ERK1/2 (4695S; 1:1,000), anti-P-ERK1/2 (4377S;1:1,000), anti-MKK7 (4172S;1:1,000), anti-MKK4 (9152S; 1:1,000) (Cell Signaling); anti-RelA (ADI-KASTF110; 1:5,000) (ENZO); anti-FLAG (F-7425;1:2,000) (Sigma-Aldrich). The anti-FLAG M2 Affinity Gel used for immunoprecipitations and kinase assays was from Sigma-Aldrich. The propidium iodide (PI; 50 µg/mL) and ribonuclease A (50 µg/mL) reagents used for the FACS analysis of apoptosis were also from Sigma-Aldrich. The reagents used for the cell treatments were: human TNFα (2,000 U/mL; Peprotech); 12-O-tetradecanoylphorbol-13-acetate (TPA; 50 ng/mL; Sigma-Aldrich); ionomycin (1 µM; Sigma-Aldrich); IL-6 (10 ng/mL; R&D Systems); IGF-1 (50 ng/mL; R&D Systems); PS-1145 dihydrochloride (20 µM; Sigma-Aldrich); SC-514 (30 µM; Calbiochem); SP600125 (10 µM; Calbiochem); Velcade (bortezomib; ranging from 0.01 nM to 10 µM; Janssen-Cilag).

Cell Culture and Primary Cell Purification

The human multiple myeloma (KMS-11, KMS-12, KMS-18, KMS-27, U266, JJN-3, NCI-H929, AMO-1, MM1.S, KMM-1, RPMI-8226 [parental]; U266/R10R, MM1/R10R, MM1.R, AMO-1a [drug resistant]), diffuse large B-cell lymphoma (DLBCL; LY3, SUDHL-6), T-cell lymphoma/leukaemia (CEM, HUT-78, Jurkat, MOLT-4, MT-2, MT-4, MT2-HTLV-I), Burkitt's lymphoma (BJAB, HS-Sultan, Namalva, RAJI, ST486), EBV-transformed B-lymphoblastoid (ARH-77), B-cell lymphoma (Karpas), chronic myelogenic leukaemia (K564), and promonocytic leukaemia (U937) cell lines were cultured in RPMI-1640 medium (Invitrogen) supplemented with 10% fetal bovine serum (FBS; Sigma-Aldrich), antibiotics (100 µg/mL penicillin, 100 µg/mL streptomycin), and 1 mM L-glutamine (Invitrogen). The human embryonic kidney (HEK-293T) and breast cancer cell lines (MCF-7, MDA-MD-231, MDA-MD-468) were cultured in high-

glucose Dulbecco’s modified Eagle’s medium (DMEM; with L-glutamine, without sodium pyruvate; Invitrogen) supplemented with 10% FBS (Sigma-Aldrich), antibiotics (100 µg/mL penicillin, 100 µg/mL streptomycin), and 1 mM L-glutamine (Invitrogen). Cells were cultured in a humidified incubator in 5% CO_2 at 37°C. The ARH-77, NCI-H929, RPMI-8226, MM1.S, MM1.R, and HEK-293T cell lines were purchased from the ATCC. The U266 and KMS-12 cell lines were purchased from the Istituto Zooprofilattico “Ugo Umbertini” (Brescia, Italy). The KMS-11, KMS-18, KMS-27, JJN-3, KMM-1, LY3, and SUDHL-6 cell lines were obtained from G. Inghirami (Turin, Italy). The lenalidomide-resistant U266/R10R and MM1/R10R cell lines were kindly provided by R. Orlowski (Houston, TX, USA) (Bjorklund et al., 2011; Bjorklund et al., 2014). The AMO-1 and bortezomib-resistant AMO-1a cell lines were from the C. Driessen’s laboratory (Ruckrich et al., 2009). All the other cell lines were from the G. Franzoso’s laboratory.

The human peripheral blood mononuclear cells (PBMCs) were purified from the blood of healthy volunteers by Ficoll-Hypaque (GE Healthcare) density separation, and cultured in RPMI-1640 medium supplemented with 10% FBS (Sigma-Aldrich), antibiotics, and 1 mM L-glutamine, as described previously (Piva et al., 2008). The human mesenchymal stem cells (MSCs) were provided by F. Dazzi (Vianello et al., 2010), and cultured in high-glucose DMEM (with L-glutamine, without sodium pyruvate; Invitrogen) supplemented with 10% FBS (Sigma-Aldrich), antibiotics (100 µg/mL penicillin, 100 µg/mL streptomycin), and 1 mM L-glutamine (Invitrogen). The human bone marrow stromal cells (BMSCs) were isolated from the bone marrow aspirates of patients with multiple myeloma, as reported elsewhere (Piva et al., 2008). Briefly, bone marrow cells were incubated in 100 mm^2 tissue culture dishes at 37°C, and, 24 hr later, non-adherent cells were removed by washing. Adherent cells with fibroblast morphology were cultured in Iscove's modified Dulbecco's medium (IMDM; Invitrogen) supplemented with 20% FBS (Sigma-Aldrich), antibiotics (100 µg/mL penicillin, 100 µg/mL streptomycin), and 2 mM L-glutamine (Invitrogen) to confluence, and then stored in aliquots in liquid nitrogen until they were used. $CD138^+$ plasma cells (PCs) were purified from the bone marrow aspirates of patients with multiple myeloma (MM), monoclonal gammopathy of undetermined significance (MGUS), Waldenström's macroglobulinemia (WM) – a lympho-proliferative disorder of B cells (Schuster et al., 2010; Kyle and Rajkumar, 2009) – or an unrelated disease without PC dyscrasia (normal) by Ficoll-Hypaque (GE Healthcare) density separation followed by positive selection with CD138 MicroBeads (Miltenyi Biotech), according to the manufacturer’s instructions. Purified PCs were cultured in RPMI-1640

medium (Invitrogen) supplemented with 10% fetal bovine serum (FBS; Sigma-Aldrich), antibiotics (100 µg/mL penicillin, 100 µg/mL streptomycin), and 1 mM L-glutamine (Invitrogen), and cell purity after MicroBeads separation was verified by flow cytometry using a FITC-conjugated anti-CD138 antibody (BD Pharmingen™).

Primary murine cells were isolated from the spleen or lymph nodes of C57BL/6 mice using standard protocols (Ahmad et al., 2011), after removal of the red blood cells with RBC lysis buffer (Sigma-Aldrich), and then seeded into wells of 96-well plates at a concentration of $6x10^4$ cells/well and cultured in RPMI-1640 medium supplemented with 10% FBS (Sigma-Aldrich), antibiotics (100 µg/mL penicillin and 100 µg/mL streptomycin) and 1 mM L-glutamine (Invitrogen).

Lentiviral Vectors

The DNA sequences of the targeting small hairpin (sh)-RNAs are listed below:

Gene and nucleotide position	targeting sequence
sh-GADD45β (262-280 bp)	3'-CAGAAGATGCAGACGGTGA-5'
sh-GADD45β-2 (352-371bp)	3'-CCAAGTTGATGAATGTGGA- 5'
sh-GADD45β-3 (436-454 bp)	3'-CAAATCCACTTCACGCTCA-5'
sh-RelA (538-556 bp)	3'-GCATCCAGACCAACAACAA-5'
sh-JNK1 (435669-435689 bp)	3'-CCTGACAAGCAGTTAGATGAA-5'
sh-MKK7 (331-349 bp)	3'-GCATTGAGATTGACCAGAA-5'
sh-MKK7-2 (1155-1173 bp)	3'-GATCACAGGAAGAGACCAA-5'
sh-ns	3'-CAGTCGCGTTTGCGACTGG-5'
sh-ns-2	3'-AAGTATGGTGAGCACGCGT-5'

The DNA oligonucleotides encoding the sh-RNAs specific for human RelA (sh-RelA), human MKK7 (sh-MKK7, sh-MKK7-2), human GADD45β (sh-GADD45β, sh-GADD45β-2, sh-GADD45β-3), or human JNK1 (sh-JNK1) were introduced between the XhoI and HpaI restriction sites of the lentiviral vector, pLentiLox3.7, as described previously (Mauro et al., 2011; Yang et al., 2006). The pLentiLox3.7 plasmids, sh-ns and sh-ns-2, expressing non-specific (ns) sh-RNAs were also described previously (Mauro et al., 2011).

Lentivirus Production and Infections

The production of high-titer lentiviral preparations in HEK-293T cells and lentiviral infections were performed as described previously (Mauro et al., 2011; Yang et al., 2006). Enhanced green

fluorescent protein (eGFP)$^+$ cells from myeloma cell lines infected with pLentiLox.3.7 lentivirus (expressing the targeting sh-RNAs and eGFP) were purified, where necessary, by FACS sorting using a BD FACSAria™ II cell sorter, and then rested in culture for at least 2 days prior to using them for further analysis.

Cell Proliferation, Cell Death, and Apoptosis Assays

[^{3}H]Thymidine incorporation assays were performed using standard protocols (Griffiths and Sundaram, 2011). Briefly, cell lines were seeded into wells of 96-well plates at a concentration of 1.0×10^4 cells/well and then left untreated or treated daily with the indicated concentrations of peptides and maintained in complete RPMI-1640 medium at 37°C in 5% CO_2, splitting them with medium as necessary. At 24, 72 or 144 hr, cells were incubated for an additional 16 hr with 0.037 MBq/well of [^{3}H]thymidine (Amersham), and then harvested onto glass fibre filter mats using a 96-well plate automated Tomtec cell harvester (Receptor Technologies) and analyzed by liquid scintillation spectroscopy with a LKB Wallac Trilux Microbeta 3 β-counter (PerkinElmer). Values were expressed as the percentage of the counts per minute (cpm) measured in the treated cultures relative to the cpm measured in the respective untreated cultures. The half-maximum inhibitory concentration (IC_{50}) values were calculated using either 5 or 7 concentrations of compound and were defined as the mean concentration of compound inducing 50% inhibition of [^{3}H]thymidine uptake relative to the uptake measured in untreated cells. Trypan blue exclusion assays were performed as reported previously (Mauro et al., 2011; Arnold et al., 2008). Briefly, cells from lentivirus-infected cell lines were seeded into wells of 48-well plates in complete medium at a concentration of 2.0×10^5 cells/well, and then cultured at 37°C in 5% CO_2, splitting them as necessary during the assays. Cell viability was monitored over a period of up to 8 days by cell counting using trypan blue, and the numbers of live infected cells in the cultures were extrapolated, where appropriate, from the cell counts by accounting for the percentages of eGFP$^+$ cells, using flow cytometry. Values were expressed as the percentage of live infected cells present in the cultures at the times indicated relative to the number of live infected cells present in the same cultures at day 0.

Apoptosis analyses were performed using propidium iodide (PI) nuclear labeling, as described previously (Riccardi and Nicoletti, 2006). Briefly, cells were seeded at a density of 2×10^5 cells/well into wells of either 24- or 48-well plates and cultured in the presence or absence of treatment, as shown.

Cells were then fixed in 70% ice-cold ethanol for 16 hr, washed in PBS, and treated with ribonuclease A (50 μg/mL) for 30 min at room temperature. Samples were finally stained with PI (50 μg/mL) in 0.1% sodium citrate buffer for 2 to 4 hr at 4°C in the dark, and data were acquired using a FACSCalibur automated system (Becton Dickinson) and analyzed with FlowJo.

The viability of primary $CD138^+$ cells from patients was assessed using trypan blue exclusion assays (Mauro et al., 2011; Arnold et al., 2008), performed as described above. Cells were seeded into wells of 96-well plates at a concentration of $3.0x10^4$ cells/well in complete RPMI-1640 medium, and then left untreated or treated daily with peptides or bortezomib for 48 hr. The IC_{50} values were calculated using at least 7 concentrations of each compound and defined as the mean concentration of compound inducing 50% of cell killing relative to the extent of cell death observed in the untreated cells (Arnold et al., 2008). For the analyses shown in Figure 6C, purified $CD138^+$ cells were seeded as above at a concentration of $3.0x10^4$ cells/well onto a monolayer of 50-60%-confluent BMSCs in 96-well plates and, then, left untreated or treated with DTP3 (300 nM) or PS-1145 (20 μM) for 48 hr (Piva et al., 2008). Where indicated, $CD138^+$ cells were treated with DTP3 or PS-1145 as above in the presence of IL-6 (10 ng/mL) or IGF-1 (50 ng/mL) and in the absence of BMSCs (Yang et al., 2007) (Figures S6A-B). Cell viability in the treated cultures was expressed as the percentage of cell survival relative to the survival observed in the respective untreated cultures, represented as 100%.

Human PBMCs, BMSCs and MSCs were left untreated or treated with D-peptide, PS-1145 or bortezomib and cultured in the manner described above for up to 144 hr. Splenocytes were treated with Ultra-pure lipopolysaccharide (LPS; 1 ng/mL; ENZO) for 16 hr, and, then, both splenocytes and lymph node cells were treated with D-peptide, PS-1145 or bortezomib for 72 hr. Cell viability was measured by using trypan blue exclusion or [^{3}H]thymidine incorporation assays performed in the manner described above (Mauro et al., 2011; Griffiths and Sundaram, 2011; Arnold et al., 2008), as indicated.

Drug Synergism

The activities of DTP3 and bortezomib in the U266 and KMS-12 myeloma cell lines, both as single agents and in combination, were determined using [^{3}H]thymidine incorporation assays, performed as described above in "Cell Proliferation, Cell Death, and Apoptosis Assays". The IC_{50} values of bortezomib as single agent were determined at 48 hr using 9 concentrations of drug, ranging from 0.1

to 1,000 nM. The IC_{50} values of DTP3 at day 6 as single agent were from the experiment in Figures S5F-G. The concentrations of DTP3 for the drug interaction studies with bortezomib were selected in pilot experiments. For these studies, U266 and KMS-12 MM cells were treated with DTP3 for 6 days at the concentrations of 3 nM and 10 nM, respectively, and bortezomib was added to the cultures at increasing concentrations, ranging from 0.1 to 1,000 nM, 48 hr prior to the measurement of cell viability by [^{3}H]thymidine incorporation. Viability data were converted into values representing the fraction of cells affected (FA) in the drug-treated cultures compared with untreated cultures, and the interaction between DTP3 and bortezomib was analyzed using the Chou-Talalay method (Chou, 2006). The combination index (CI) was calculated based upon this method according to the following equation:

$$CI = (D)_1 / (Dx)_1 + (D)_2 / (Dx)_2$$

Where $(D)_1$ and $(D)_2$ are the doses of drug 1 (DTP3) and drug 2 (bortezomib), respectively, having x effect when used in combination, and $(Dx)_1$ and $(Dx)_2$ are the doses of drug 1 and drug 2, respectively, having the same x effect when used alone. Synergy is present when the CI is less than 1.0 (where values between 0.3 and 0.7 denote synergism, and values between 0.1 and 0.3 denote strong synergism); the combination is additive when CI equals 1.0, and antagonistic when it is more than 1.0 (Chou, 2006).

Quantitative Real-Time Polymerase-Chain Reaction (qRT-PCR)

Total RNA was extracted with Trizol (Invitrogen) and purified using the RNAeasy Mini-Kit (Qiagen), according to the manufacturer's instructions. 1 µg of RNA was added as template to reverse-transcriptase (RT) reactions, performed using the GeneAmp RNA PCR Kit (Applied Biosystems) (Mauro et al., 2011). qRT-PCRs were carried out with the resulting cDNAs using the TaqMan® Gene Expression Assays kit (Applied Biosystems), appropriate gene-specific primer/probe sets (Applied Biosystems), and an ABI 7900 real-time PCR machine (Applied Biosystems), according the manufacturer's instructions. Experimental ΔΔCt values were normalized to 18s ribosomal RNA, *β-Actin* or *GUSB β-Glucuronidase*, and relative mRNA levels were calculated against a reference sample (i.e. mRNA from HEK-293T cells, for cell lines; mRNA from PBMCs or BMSCs, for primary cells).

Co-Immunoprecipitation and Kinase Assays

Co-immunoprecipitation assays were performed as described previously (Papa et al., 2004). Briefly, human HEK-293T cells were seeded at a density of 1.8-2.2x10^6 cells/dish in 100 mm^2 tissue-culture dishes and then transfected with pcDNA-FLAG-hMKK7 and pcDNA-HA-hGADD45β by using the calcium phosphate precipitation method (Papa et al., 2004). 48 hr after transfection, cells were washed with PBS and then lysed in lysis buffer supplemented with protease inhibitors (1 mM phenylmethylsulfonylfluoride, 10 µM chymostatin, 2 µg/mL aprotinin, 2 µg/mL leupeptin; Roche) for 30 min in ice. Cell lysates were cleared by centrifugation at 45,000 x *g* for 40 min and then used for co-immunoprecipitations with anti-FLAG M2 Affinity Gel (Sigma-Aldrich), binding to FLAG-tagged MKK7. Western blots were developed using an anti-HA (binding to HA-hGADD45β) or an anti-MKK7 antibody. For competition assays (Figure 3C, Figure S5E), the anti-FLAG immunoprecipitates were incubated for 2-4 hr at 4°C in the presence or absence of bioactive or control inactive D-peptides, before loading them onto SDS-PAGE gels.

MKK7 kinase assays were performed as described previously (Papa et al., 2008; Papa et al., 2004) (Figure 3D). Briefly, HEK-293T cells were transfected with pcDNA-FLAG-hMKK7 using the calcium phosphate precipitation method, as above. 36 hr after transfection, cells were treated with 12-O-tetradecanoylphorbol-13-acetate (TPA; 100 ng/mL) and ionomycin (1 µM) for 30 min at 37°C or left untreated and, then, lysed in lysis buffer and used for immunoprecipitations with anti-FLAG M2 Affinity Gel, as above. In order to measure MKK7 activity, immunoprecipitates were washed in lysis buffer and twice more in kinase buffer (10 mM HEPES, 5 mM $MgCl_2$, 1 mM $MnCl_2$, 12.5 mM β-glycerophosphate, 2 mM DTT, 4 mM NaF, 0.1 mM Na_3VO_4) and, then, incubated with recombinant GST-JNK1 (2 µM) in kinase buffer for 10 min at 30°C, followed by incubation with 5 µCi [γ-^{32}P]ATP (kinase reaction). Where indicated, anti-FLAG-MKK7 immunoprecipitates were pre-incubated with D-tetrapeptides for 10 min at 30°C and, then, incubated for another 10 min at 30°C in the presence or absence of recombinant GST-hGADD45β (5 µM), before setting up the kinase reaction as above. Reactions were stopped with Laemmli sample buffer, and ^{32}P-labelled proteins were resolved by 10% SDS-PAGE and revealed by autoradiography. Inactive MKK7 was immunoprecipitated from untreated HEK-293T cells. The amino acid sequences of the active and inactive control D-peptides used in the co-immunoprecipitation and/or kinase assays were as follows: Ac-DTP1, Ac-Tyr-Asp-His-Phe-NH_2; Ac-DTP2, Ac-Tyr-Glu-Arg-Phe-NH_2; Ac-DNC, Ac-Tyr-Asp-His-Gln-NH_2; Ac-DNC2, Ac-Tyr-Asp-His-Ala-NH_2; Ac-DNC3, Ac-Tyr-Glu-

Lys-Trp-NH_2; Ac-DNC4, Ac-Tyr-Asp-Lys-Trp-NH_2; DTP3, Ac-Tyr-Arg-Phe-NH_2; scrambled (SCRB), Ac-Arg-Phe-Tyr-NH_2. All peptides were in the D configuration.

JNK kinase assays were performed as described previously (Papa et al., 2008) (Figure 5D). MM cells ($1.0x10^6$/mL) were left untreated or treated with DTP3 (10 μM) or TNFα (2,000 U/mL) for the times indicated and then lysed in lysis buffer (Papa et al., 2004), as described above. Cell extracts were used for immunoprecipitations with anti-JNK antibody immobilized onto Protein A/G PLUS-agarose beads (sc-2003; Santa Cruz Biotechnology), and kinase assays were performed as described above, using recombinant GST-cJun (2 μM) as substrate.

Peptide Library Design and Synthesis

The combinatorial tetrapeptide libraries were synthesised in a simplified format using 12 L-amino acids (GL-Biochem; Novabiochem) occupying different chemical spaces, as reported previously (Sandomenico et al., 2012). The amino acids used were as follows: Asp (D), representing acidic amino acids; R (Arg) and His (H), representing basic amino acids, with His also representing aromatic residues; Gln (Q) and S-acetamydomethyl(Acm)-Cys (referred to as C), representing amidated side chains; Phe (F) and Tyr (Y), representing aromatic amino acids; Ser (S), representing amino acids containing hydroxyl groups; Leu (L) and Met (M), representing bulky hydrophobic amino acids; Ala (A), representing small hydrophobic amino acids. Pro (P) was used to generate peptides with a bent conformation. The L-tetrapeptides were joined to an N-terminal β-Ala_2 spacer linked to a fluorenylmethyloxycarbonyl (Fmoc) tag and were synthesised by the solid phase method using conventional Fmoc/tBu chemistry, as reported previously (Sandomenico et al., 2012). Random amino acid positions were filled in by using equimolar mixtures of the 12 L-amino acids in large excess, in order to suppress preferential acylations. The L-tetrapeptides contained a C-terminal amide group and were cleaved from the resin using trifluoroacetic acid (TFA)/tri-isopropylsilane (TIS)/water (90:5:5, v/v/v; Sigma-Aldrich) and purified to homogeneity by semi-preparative reverse phase (RP)-HPLC. Peptide identity and purity were determined using liquid chromatography-mass spectrometry (LC-MS), as described below. Peptides were then lyophilized and dissolved in dimethylsulphoxide (DMSO; Sigma-Aldrich) at a concentration of 10 mg/mL (stock solution) and stored in aliquots at -20°C until they were used.

The initial library contained a total of 20,736 (12^4) tetrapeptides, formed by all possible combinations of the 12 L-amino acids, and was iteratively deconvoluted in four steps by ELISA GADD45β/MKK7 competition assays, as described below in "Protein Purification and ELISA". Each of the peptide mixtures used for the subsequent steps of the screen was prepared in the same manner and on the same scale. Step 3 of the screen identified individual L-tetrapeptides (Table S1), the most active of which (i.e. Fmoc-β-Ala_2-Tyr-Asp-His-Phe-NH_2) was used as template for the synthesis of 24 second-generation L-tetrapeptides in which the N-terminal Fmoc-β-Ala_2 tag was replaced with an acetyl (Ac) group (Table S2). All individual peptides were prepared using the same solid-phase Fmoc/tBu methodology and, then, purified and characterized in the same manner as for the tetrapeptide libraries (see below).

N-terminal acetylation was carried out by treatment with 10% acetic anhydride in dimethylformammide (DMF; Sigma-Aldrich) containing 5% diisopropyl-ethylamine (DIEA; Sigma-Aldrich). As part of the structure-activity relationship (SAR) analysis, in order to improve their stability and cellular activity, peptides were synthesized in the D configuration using the same solid-phase method and Fmoc-protected D-amino acids (GL-Biochem; Novabiochem), as described above (Sandomenico et al., 2012). To increase cellular uptake, where indicated, D-peptides were modified by the introduction of various chemical groups, including a benzyloxycarbonyl (Z), O-methyl, 2-chloro-benzyloxycarbonyl (2Cl-Z), myristyl (Myr), benzoyl, 3-hydroxy-4-methoxybenzyl, or Fmoc group, as shown (Table S3). The Z group was introduced by on-resin treatment of the peptides with 0.5 M benzyloxycarbonyl-N-hydroxysuccinimide (Z-OSu; GL-Biochem) in DMF with 5% DIEA. The carboxylic acids used to derivatize the N-terminus of other peptides were pre-activated with 2-(1H-7-azabenzotriazol-1-yl)-1,1,3,3-tetramethyluronium hexafluorophosphate (HATU; GL-Biochem) and DIEA, and subsequently coupled to the resin.

Peptide Characterisation by Liquid Chromatography-Mass Spectrometry (LC-MS)

The identity and purity of each of the peptides were determined by liquid chromatography-mass spectrometry (LC-MS) using an ion trap LCQ Deca XP mass spectrometer (ThermoFisher), equipped with an Opton ESI source operated at 5 kV and 300 °C, coupled to an LC system comprising a Surveyor HPLC and a Biobasic 50x2 mm ID C18 column (ThermoFisher). The peptide spectra were acquired in the positive mode between 200 and 2,000 m/z, using approximately 50 ng of peptide and a

flow rate of 0.20 mL/min, by applying a gradient from 5% to 70% of solvent B (CH_3CN, 0.05% TFA) over solvent A (H_2O, 0.08% TFA), within a time window of 10 min. Where necessary, the gradient was changed in order to elute the compounds within a time window suitable for MS and diode array analyses. Peptide purity was assessed on the basis of the HPLC traces at 214 nm, by area integration of all the recorded peaks. Peptide identity was established on the basis of the experimental molecular weight (MW). The amino acid sequence, the calculated and experimental MW, and the purity level of each of the main peptides used are listed below:

amino acid sequence	$MW_{calcd, m*}$ (g/mol)	$MW_{exp, m*}$ (g/mol)	Purity, LC-MS (%)
Ac-YDHF-NH_2 (Ac-LTP1)	621.25	621.3	97
Ac-YEHF-NH_2	635.27	635.3	95
Ac-WDHF-NH_2	644.27	644.4	97
Ac-WEHF-NH_2	658.29	658.4	96
Ac-YDRF-NH_2	640.30	640.5	95
Ac-YDKF-NH_2	612.29	612.2	95
Ac-YEKF-NH_2	626.31	626.1	95
Ac-YERF-NH_2 (Ac-LTP2)	654.31	654.3	96
Ac-WEKF-NH_2	649.32	649.4	97
Ac-WERF-NH_2	677.35	677.2	95
Ac-WDKF-NH_2	653.31	653.1	96
Ac-WDRF-NH_2	663.31	663.3	97
Ac-YDHW-NH_2	660.27	660.2	95
Ac-YEHW-NH_2	674.28	674.4	97
Ac-WDHW-NH_2	683.28	683.4	> 99
Ac-WEHW-NH_2	697.30	697.2	97
Ac-YDRW-NH_2	679.31	679.4	95
Ac-YDKW-NH_2	651.30	651.3	96
Ac-YEKW-NH_2	655.32	655.3	> 99
Ac-YERW-NH_2	693.32	693.4	96
Ac-WEKW-NH_2	688.33	688.2	97
Ac-WERW-NH_2	716.34	716.4	97
Ac-WDKW-NH_2	674.32	674.3	97
Ac-WDRW-NH_2	702.32	702.2	97
z-YERF-NH_2 (z-DTP2)	746.35	746.3	97
z-YDHF-NH_2 (z-DTP1)	713.29	713.3	97

z-YDHQ-NH_2 (z-DNC)	694.27	694.4	96
z-YD(O-methyl)HF-NH_2	727.48	727.7	95
z-YD(O-methyl)HQ-NH_2	708.27	709.5	95
2Cl-z-YERF-NH_2	780.50	780.4	96
2Cl-z-YDHF-NH_2	747.26	747.3	96
2Cl-z-YDHQ-NH_2	728.23	728.3	97
Myr-YERF-NH_2	823.68	822.9	97
Myr-YDHF-NH_2	789.60	788.8	95
Myr-YDHQ-NH_2	770.63	769.9	97
Benzoyl-YERF-NH_2	717.90	717.0	97
Benzoyl-YDHF-NH_2	593.90	683.6	97
Benzoyl-YDHQ-NH_2	665.90	665.4	98
3-hydroxy-4-methoxy-benzyl-YERF-NH_2	755.30	755.2	95
3-hydroxy-4-methoxy-benzyl-YDHF-NH_2	722.24	722.2	95
3-hydroxy-4-methoxy-benzyl-YDHQ-NH_2	703.23	703.3	95
Fmoc-YERF-NH_2	834.37	834.2	99
Fmoc-YDHF-NH_2	801.31	801.2	99
Fmoc-YDFQ-NH_2	782.30	781.7	99
Ac-YERFK(εZ)-NH_2	916.32	916.2	97
Ac-YDHFK(εZ)-NH_2	882.60	882.8	97
Ac-YDHQK(εZ)-NH_2	864.42	864.5	96
Ac-YQRF-NH2	653.33	653.2	95
Ac-YNRF-NH2	639.31	639.5	95
Ac-YLRF-NH2	638.35	638.5	96
Ac-YLF-NH2	497.26	497.3	96
Ac-YNF-NH2	483.21	483.2	97
Ac-YPF-NH2	466.22	466.1	97
Ac-YHF-NH2	506.23	506.2	96
H-YHF-NH2	464.22	464.2	98
Ac-YRY-NH2	541.26	541.3	98
H-YRY-NH2	499.25	499.4	> 99
Ac-YKF-NH2	497.26	497.1	98
Ac-YRF-NH2 (DTP3)	525.27	525.3	> 99

calcd, m* = calculated monoisotopic
exp, m* = experimental value

The peptides were lyophilized and dissolved in dimethylsulphoxide (DMSO; Sigma-Aldrich) at a concentration of 10 mg/mL (stock solution) and then stored in aliquots at -20°C until they were used.

High-Resolution Mass Spectrometry (MS) Analysis of z-DTP1, z-DTP2, and DTP3

For a more accurate mass measurement, the lead peptides, z-DTP1, z-DTP2 and DTP3, were analyzed in the positive ion mode using an electrospray hybrid quadrupole orthogonal acceleration time-of-flight mass spectrometer (Q-TOF) fitted with a Z-spray electrospray ion source. The capillary source voltage and the cone voltage were set at 3,500 V and 35 V, respectively; the source temperature was maintained at 80 °C; nitrogen was used as the drying gas, at the flow rate of approximately 50 L/hr. The time-of-flight analyzer was calibrated using selected fragment ions resulting from the collision-induced decomposition of human (Glu1)-fibrinopeptide B (500 fmol/mL in CH_3CN:H_2O [50:50], 0.1% formic acid) at an infusion rate of 10 µL/min in the TOF MS/MS mode. The mass data were then collected by infusing the samples (3 pmol/µL in 1:1 CH_3CN:H_2O) into the system at a flow rate of 10 µL/min. Data acquisition and processing were carried out using MassLynx 4.1 software. The results of these analyses are reported below:

compound	calculated Mr* $[M+H]^+$	experimental Mr $[M+H]^+$	Δ (ppm)	Δ (Da)
z-DTP1	714.2888	714.2897	1.4	0.0009
z-DTP2	747.3466	747.3429	5.3	0.0037
DTP3	526.2778	526.2784	1.9	0.0006

* calculated considering the monoisotopic masses according to the Pure App Chem. 63(7), 975-90 (1991)

^{1}H-Nuclear Magnetic Resonance (NMR) Analysis of z-DTP1, z-DTP2, and DTP3

The identity and purity of the lead D-peptides, z-DTP1, z-DTP2 and DTP3, were also determined by ^{1}H-nuclear magnetic resonance (NMR) analysis. The NMR spectra of the peptides were acquired in D6-DMSO, at a concentration of 2 mg/mL, using a Varian 400 MHz NMR spectrometer. The measured chemical shifts are reported below:

compound	chemical shift values
z-DTP1	δ 5.02 - 4.91 ppm (m, 5H, Z group); δ 7.10, 7.12 ppm (d, 1H, H3 and H5 on Tyr side chain) and δ 6.68, 6.70 ppm (d, 1H, H2 and H6 on Tyr side chain); δ 3.12 – 3.02 ppm (m, 4H, βCH2 Asp, βCH2 His); δ 4.64 – 4.59 ppm (m, 1H, αCH His); δ 9.24 ppm (s, 1H, H2 on imidazole His); δ 8.93 ppm (s, 1H, H4 on imidazole His); δ 7.39 - 722

	ppm (m, 5H, H1-5 on the phenyl ring on the Phe side chain)
z-DTP2	δ 5.03 – 4.94 ppm (m, 5H, Z group); δ 7.12 – 7.14 ppm (d, 1H, H3 and H5 on Tyr side chain); δ 6.68 - 6.70 ppm (d, 1H, H2 and H6 on Tyr side chain); δ 4.30 – 4.21 ppm (m, 1H, αCH Phe); δ 4.51 – 4.45 ppm (m, 1H, αCH Tyr); δ 2.08 . 2.00 ppm (m, 2H, γCH2 Glu); δ 1.67 - 1.58 ppm (m, 2H, βCH2 Glu); δ 4.51 - 4.45 ppm (m, 1H, αCH Glu); δ 4.30 - 4.21 ppm (m, 3H, αCH Tyr, Phe, Arg); δ 7.43 ppm (s, 1H, Nε, Arg side chain); δ 6.68 – 6.70 ppm (d, 1H, Nη1, Arg side chain); δ 7.30 – 7.22 ppm (m, hydrogen on Phe side chains)
DTP3	δ 1.82 ppm (s, 3H, Acetyl); δ 7.10 – 7.08 ppm (d 1H, H3, H5 on Tyr side chain); δ 7.31 – 7.15 ppm (m, 5H, H1-5 on the phenyl ring on the Phe side chain);δ 4.51 – 4.45 ppm (m, 1H, αCH Arg); δ 3.01 – 3.06 ppm (m, 2H, βCH2 Arg); δ 2.90 – 2.84 ppm (m, 2H, γCH2 Arg); δ 2.70 – 2.64 ppm (m, 2H, δCH2 Arg); δ 6.67 – 6.69 ppm (d, 1H, Nη1, Arg side chain); δ 7.43 ppm (s, 1H, Nε, Arg side chain)

Protein Purification and ELISA

Recombinant proteins were purified as described previously (Tornatore et al., 2008). Briefly, full-length human (h)GADD45β was expressed as a protein fused to an N-terminal glutathione S-transferase (GST) or a poly-histidine (His_6) tag and purified by affinity chromatography using GST-Trap and His-Trap columns (GE Healthcare), respectively. The GST-GADD45β protein preparations were used for kinase assays; The His_6-GADD45β protein was used for ELISA, CD and MALDI-TOF-MS analyses. Full-length human (h)MKK7 was expressed as a protein fused to an N-terminal GST tag (GST-MKK7), purified using GST-Trap as above, and then used for ELISA and spectrofluorimetric analyses. Purified GST was used as a control in all the experiments. The kinase domain (KD) of hMKK7 (amino acids 101-405) was expressed as a protein fused to an N-terminal His_6 tag in E. coli (BL21[DE3] strain) using the pETDuet-1 vector (Novagen). The resulting His_6-hMKK7-KD protein was purified by affinity chromatography using a His-Trap column, followed by gel filtration, and utilised for CD and MALDI-TOF-MS analyses, following dilution in water or 1 mM ammonium bicarbonate, pH 7.0, as appropriate, from a stock solution prepared at 0.5 mg/mL (13.7 μM) in 25 mM TRIS, 150 mM NaCl buffer at pH 7.0.

The methods used for the ELISA binding and competition assays were described previously (Tornatore et al., 2008). Briefly, His_6-GADD45β was biotinylated using the EZ Link® NHS-LC-biotin kit (Pierce), according to the manufacturer's instructions (Tornatore et al., 2008). Human GST-MKK7 was coated onto wells of 96-well plates by incubation at 42 nM in ELISA buffer (25 mM Tris pH 7.5, 150 mM NaCl, 1 mM DTT, 1 mM EDTA) for 16 hr at 4°C, followed by blocking with 2% non-fat dry milk in PBS. Biotin-labelled His_6-hGADD45β was then added to the wells at increasing concentrations ranging from 5.2 to 168 nM and incubated in the dark for 1 hr at 37°C, followed by washing with Tween (T)-PBS.

Bound His$_6$-hGADD45β was revealed using horseradish peroxidase (HRP)-conjugated streptavidin (1:1,000) and the HRP chromogenic substrate, o-phenylenediamine (OPD). GST-coated wells were used as controls.

The ELISA competition screen used for the isolation of antagonists of the GADD45β/MKK7 complex was carried out using coated GST-hMKK7 (42 nM), soluble biotin-labelled His$_6$-hGADD45β (21 nM), and the competing tetrapeptides or tetrapeptide mixtures at the nominal concentration of 42 nM (Figures S3A-B). The library deconvolution strategy used for this screen was described previously (Sandomenico et al., 2012). General information about this type of screens can be found elsewhere (Houghten et al., 1999; Eichler and Houghten, 1995). Values were expressed as the percentage of inhibition of His$_6$-hGADD45β binding to GST-hMKK7 relative to the binding measured in the absence of competitor.

Dose-response, ELISA GADD45β/MKK7 competition assays were carried out under similar conditions, using coated GST-hMKK7 (42 nM), soluble biotin-labelled His$_6$-hGADD45β (21 nM), and the competing peptides at increasing concentrations, ranging from 0.001 to 21 nM (Figures 3A-B, Figure S4A, Figure S5D, Table S3, Tables S4). As part of the SAR analysis and lead optimization process, where appropriate, competitors were tested at a concentration up to 100 nM. In order to determine their stability in biological fluids, peptides were incubated at the concentration of 10 µM in human serum for 48 hr at 37°C and, then, used at increasing concentrations as above in ELISA GADD45β/MKK7 competition assays (Figure 3B, Figures S4A-B, Figure S5D). IC_{50} values were calculated using at least 7 concentrations of peptide and defined as the mean concentration of peptide inducing 50% inhibition of His$_6$-hGADD45β binding to GST-hMKK7 relative to the binding measured in the absence of competitor. Where indicated, values were expressed as the percentage of inhibition of His$_6$-hGADD45β binding to GST-hMKK7 relative to the binding measured in the absence of competitor. GST was used as a control in all the experiments. The amino acid sequence of the inactive D-tetrapeptide used as negative control in some of the ELISA and cellular assays was as follows: z-DNC, z-Tyr-Asp-His-Gln-NH_2.

Peptide Pull-Down Assays

For the peptide pull-down assays (Figure 5E), DTP3 and SCRB D-peptides were synthesized joined to an N-terminal β-Ala_2 spacer by the solid phase method, as described above (Sandomenico et al., 2012), without N-terminal acetylation in order to preserve a suitable anchoring amino group, and then immobilized onto Sepharose 4 Fast Flow resin (GE Healthcare). Upon synthesis, the peptides were cleaved from the Rink Amide resin used for the solid phase methodology and purified to homogeneity by RP-HPLC. Peptides were then dissolved at a concentration of 1.5 mg/mL in 2.0 mL of 25 mM phosphate buffer, pH 7.0, and incubated for 48 hr at room temperature with 1 mL of Sepharose 4 Fast Flow resin, which had been previously pre-activated with N-hydroxy-succinimide (NHS) and washed twice with the same buffer at 4°C. Coupling reactions were monitored by RP-HPLC analysis of the reaction solutions, and residual NHS groups were finally deactivated by treatment with 2.0 mL of 0.5 M TRIS pH 9.0, following the removal of the reaction solutions by washing. With each of the peptides, the efficiency of immobilization was approximately 90%, yielding peptide preparations containing approximately 2.7 mg of bound peptide per mL of Sepharose 4 Fast Flow resin. The blank resin was prepared under similar conditions, without including the peptide. The resins were stored at 4°C in 20% ethanol until they were used.

For the pull down of endogenous MKK7 from MM cells, 100 μL of resin coated with DTP3, SCRB peptide or no peptide (blank) were washed twice with 3 volumes of a buffer containing 20 mM Tris pH 7.5, 150 mM NaCl, 1 mM DTT, 1 mM EDTA, and 0.007% Triton X-100, supplemented with protease inhibitors (Roche), and subsequently incubated for 1.5 hr at room temperature with 300 μg of cell extract prepared from MM cells, as described above for co-immunoprecipitation assays. The resins were then washed three times with the same buffer, containing 0.1% of Triton X-100, and bound proteins were resolved by SDS-PAGE and revealed by western blotting using an anti-MKK7 antibody.

Circular Dichroism (CD) Analyses

CD analyses were performed using a JASCO J-710 spectropolarimeter (JASCO Corp) equipped with a Peltier temperature control system and a 110-QS quartz cuvette with 1.0-cm path length, as described previously (Tornatore et al., 2008; Sandomenico et al., 2013). Following optimisation in pilot experiments, the analyses were conducted at a pH of approximately 7.0 and a concentration of His_6-hGADD45β and His_6-hMKK7-KD of 1.25 μM, whether the proteins were used alone or in combination. In order to minimize salt interference from the solution buffers in the far-UV region of the spectrum,

proteins were diluted from their stock aliquots in water. The CD spectra of GADD45β, MKK7-KD and the GADD45β/MKK7-KD complex were acquired in the presence or absence of either DTP3 or SCRB D-peptide at increasing concentrations, ranging from 0.1 nM to 10 μM, as shown, in water, under a constant N_2 flow and using the following settings: wavelength range, 190-280 nm; scanning speed, 20 nm/min; data pitch, 0.2 nm; band width, 1 nm; response time, 4 s. The recorded spectra were then signal-averaged from at least five independent readings and smoothed, and the baseline values were corrected by subtracting the contributions of the peptides, at each of the concentrations ranging from 0.1 nM to 10 μM. Data were analyzed for conformational changes of the proteins in the near-UV region, between 200 and 280 nm.

MALDI-TOF Mass Spectrometry (MS) Analyses

MALDI-TOF-MS analyses were performed in the positive linear reflectron mode using a MALDI-TOF micro MX analyzer (Waters Co., Manchester, UK), equipped with a pulsed nitrogen laser (λ=337 nm). The instrument was calibrated using a mixture (10 pmol/mL) of insulin, cytochrome c, and trypsinogen as standard proteins (Sigma, MO, USA) and the recommended polynomial equation, according to the manufacturer’s instructions. Spectra were processed and analyzed using MassLynx 4.0 software. The instrument source voltage was set at 12 kV. The pulse and detector voltages were set at 1,999 V and 5,200 V, respectively. Measurements were carried out using a suppression mass gate set at 5,000 m/z and an extraction delay of 600 ns. Data were acquired by measuring and averaging at least 10 spectra, chosen randomly over the well surface, and the spectra were subsequently smoothed and overlaid. Spectra acquisition was optimized by performing a scouting procedure prior to the analyses, using saturated sinapinic acid (10 mg/mL in acetonitrile/trifluoroacetic acid 0.1%, 2:3, v:v) and α-cyano-4-4-hydroxycinnamic acid matrix solution (10 mg/ml in acetonitrile/trifluoroacetic acid 0.1%, 1:1, v:v) as ionizing matrices. To acquire the spectra, 1 μL of the matrix solution, 1 μL of the protein solution in 1 mM of ammonium bicarbonate, pH 7.0, and 1 μL of the matrix were sequentially spotted onto the target plate, letting the solvent evaporate at each of the steps. For the analyses shown in Figure S5J, His_6-hMKK7-KD and His_6-GADD45β were used at the concentration of 5 μM, following incubation for 10 min at room temperature in the presence or absence of a 5-μM concentration of DTP3 or SCRB D-peptide. Peptides were dissolved in water in order to minimize the salt contents of the samples.

Spectrofluorimetric Analyses

The stoichiometry of the interaction of DTP3 with MKK7 was determined by using tryptophan fluorescence quenching analysis. Spectra were acquired with a Cary Eclipse Spectrofluorimeter analyzer (Varian Inc.), equipped with Peltier thermostating system, and a 1-cm quartz cuvette. The analyses were carried out using 1.25 μM of GST-hMKK7 or GST dissolved in 10 mM of phosphate buffer, pH 7.5, and increasing concentrations of DTP3 or control SCRB D-peptide dissolved in the same buffer. The data were acquired at 25.0 °C, using an excitation wavelength of 295.0 nm, in order to avoid interference with tyrosine residues, and a fluorescence emission wavelength ranging from 300 nm to 450 nm. The excitation and emission slits were set at 5 nm; the scan rate was 120 nm/min; the data interval was 1.00 nm; the averaging time was set at 0.500 s; the PMT voltage was set at “high.” All the spectra were smoothed using the same algorithm. The fluorescence values recorded at 333 nm were extracted, transformed to -ΔFluorescence and, then, plotted against the peptide concentration.

Fluorescence data were fitted using a single-site binding model (equation 1):

$$PL = P + L \qquad (1)$$

Accordingly, the equilibrium dissociation constant (K_D) of the DTP3/MKK7 interaction was given by equation 2:

$$K_D = [P][L]/[PL] \qquad (2)$$

Where [P], [L] and [PL] are the equilibrium concentrations of the protein, the ligand and the protein-ligand complex, respectively. For each measurement, the observed fluorescence value, F_{obs}, was given by the population-weighted combination of the fluorescence values in the unbound and bound protein states:

$$F_{obs} = P_b F_b + P_u F_u = P_b(F_b - F_u) + F_u \qquad (3)$$

Where P_u and P_b are the populations of the unbound and bound states, respectively, and F_u and F_b are the intrinsic fluorescence values for these two states. Note that $P_u + P_b = 1$. P_b can be expressed in terms of [P], [L], [PL] and K_D according to:

$$P_b = \frac{[PL]}{[P]+[PL]} = \frac{[P][L]}{K_D\left([P]+{[P][L]}/{K_D}\right)} \qquad (4)$$

The fitting formula employed here is the combination of equations 3 and 4:

$$\Delta F = F_{obs} - F_u = \frac{[P][L]}{K_D\left([P] + {[P][L]}/{K_D}\right)}(F_b - F_u) \quad (5)$$

The quality of the data fitting suggested that a single site model, i.e. a 1 to 1 stoichiometry of binding, was a highly appropriate model to account for the measured florescence trends, resulting in a R square (R^2) of 0.985 and a chi square (X^2) of 1.436. Based on this assay, the estimated K_D value of the interaction of DTP3 with MKK7 was 64.81 +/- 6.22 nM. Data were fitted using a non-linear regression algorithm (Origin, version 6.0) (Williamson, 2013).

The emission spectra of the D-peptide solutions were acquired under the same conditions, using up to 300 µM of DTP3 or SCRB D-peptide, in order to exclude interference from tyrosine fluorescence.

GADD45β and MKK7 Modeling

The structure of human GADD45β was built by homology modeling. The 14 N-terminal amino acids of this protein were predicted by the GeneSilico MetaDisorder server (verified to be the best performing method in preliminary CASP8 and CASP9 blind procedures) (Kozlowski and Bujnicki, 2012) to be disordered and were, therefore, excluded from subsequent analyses. The GADD45β amino acid region spanning residues 15-160 was predicted to fold as a globular domain, with a defined pattern of secondary structure, and was used to search the Protein Data Bank (PDB) with the HHpred server (Söding et al., 2005). The top scoring hit resulting from this search corresponded to the crystal structure of human GADD45γ (PDB ID: 3FFM, chain A), covering the amino acid region from residue 15 to 157, which shares a 58% amino acid sequence identity with GADD45β. The probability of this being a true positive was nearly 100% (E-value = 5.3×10^{-52}). The homology model of GADD45β was subsequently built from the structure of GADD45γ using Modeller (Eswar et al., 2007). We generated 50 models of the GADD45β structure and selected the final model according to the Modeller objective function. Hydrogen atoms were added using HAAD software (Li et al., 2009). The selected model was then refined using the FG-MD molecular dynamics algorithm (Zhang et al., 2011). The quality of this refined model was assessed using the QMEAN (Benkert et al., 2011) and ProQ model quality assessment programs (Wallnerand and Elofsson, 2003). The QMEAN score of this model was 0.78 (within a range from 0 to 1, with 1 indicating a perfect model); the ProQ scores were as follows:

MaxSub = 0.74; LGscore = 5.47 (with MaxSub >0.5 and LGscore >4, corresponding to very good and extremely good model quality, respectively) (Figure S5L).

The deposited crystal structure of the kinase domain of human MKK7 (PDB ID: 2DYL) was re-refined by the use of autoBUSTER-TNT 1.11 (Blanc et al., 2004), because portions of this protein had been incorrectly traced. The phase distribution obtained from this analysis was subjected to density modification as implemented by Parrot (Cowtan, 2010). The resulting map was used for model building by ARP/warp (Cohen et al., 2008). The missing residues were modeled by using the loop tool option available in Modeller. The QMEAN score of the final model of MKK7 was 0.70; the ProQ scores were as follows: MaxSub = 0.6; LGscore = 6.0 (Figure S5M).

GADD45β/MKK7 Complex Modeling

The model of the mode of interaction of GADD45β with the kinase domain of MKK7 was built from the models of GADD45β and MKK7, obtained as described above, by applying the protein-protein docking procedure implemented in the ClusPro 2.0 web server (http://cluspro.bu.edu) (Kozakov et al., 2010) with default parameters. Published data from site-directed mutagenesis and in vitro binding studies (Papa et al., 2007) were taken into account by providing the following amino acid residues as restraints: MKK7, residues Val134, Trp135, Lys136, Arg138, Lys149, and Arg162; GADD45β, residues Glu63, Glu64, Asp67, Asp68, His74, Gln109, Thr111, Trp112, Glu113, Arg115, and Leu117. The centroid of the largest cluster of solutions – two solutions were considered within the same cluster when the root mean square deviation of their backbone atoms was below 9 Å – was selected as the model of the complex, based on established criteria (Kozakov et al., 2010). This selected model was finally refined in explicit solvent using the HADDOCK web server (Van Dijk and Bonvin, 2010) (http://haddock.science.uu.nl/services/HADDOCK/haddockserver-refinement.html) (Figure S5N).

Binding Site Prediction

The FTSite (Ngan et al., 2012) and FLAP (fingerprints for ligands and proteins) (Carosati et al., 2004) algorithms were used to identify potential ligand-binding sites on the experimental structure of MKK7. The strategy of FTSite consists in exploring the potential interactions of the surface regions of a protein with 16 small organic molecules, which vary in size and shape, and has been shown to be effective in detecting ‘hot spots’ involved in the binding of proteins to drug-size ligands (Kozakov et al., 2011)

(Figure S5O). FLAP, which was also used for the building of the pharmacophore model and the docking of this model to the putative ligand-binding region of MKK7 (Figure S5A, Figures S5Q-S; see also Figure S5T), relies on the GRID molecular interaction fields (MIFs) (Baroni et al., 2007) and takes into account shape, hydrophobic interactions, H-bond donor, and H-bond acceptor interactions (Figure S5P).

Pharmacophore Model Building

The pharmacophore model was generated using FLAP software (Figure S5A) (Baroni et al., 2007), starting from 12 bioactive peptides exhibiting an IC_{50} value less than or equal to 10 nM (Table S4). The three-dimensional structure of the peptides was built using the MarvinSketch server (http://www.chemaxon.com/marvin/sketch/index.jsp) and subsequently minimized. 100 conformers were generated for each of the peptides used as input for FLAP. The pharmacophore predictive capacity was subsequently tested by using an additional set of 10 active and 16 inactive peptides, defined on the basis of their activity in ELISA GADD45β/MKK7 competition assays as follows: active peptides, exhibiting a percentage of binding inhibition of more than 60%; inactive peptides, exhibiting a percentage of binding inhibition of less than 30% (Table S4; see also Figure S5B).

Broad-Panel Kinase Profiling

DTP3 was tested for activity at the concentration of either 1 or 10 μM against a panel of 142 human kinases. The kinases used in the assay and their activities in the presence or absence of DTP3 are listed in Table S5. The profiling study was outsourced to the International Centre for Kinase Profiling, MRC-Protein Phosphorylation Unit, Dundee (http://www.kinase-screen.mrc.ac.uk/), and performed according to in-house protocols therein.

Pharmacokinetic Analyses

In vitro and in vivo pharmacokinetic analyses were outsourced to Cyprotex (http://www.cyprotex.com/home/) (Figure 7A, Tables S6). In vitro pharmacokinetic assays were performed according to established, in-house protocols and included: plasma stability (human), thermodynamic solubility (PBS pH 7.4, at 37°C for 14 days), microsomal stability (human), plasma protein binding (PPB; mouse and human), and distribution coefficient (LogD). In vivo pharmacokinetic

analyses were also performed at Cyprotex according to established, in-house protocols. Briefly, the peptides were dissolved in PBS and administered by single intravenous injection to CD1 male mice at the dose of 10 mg/kg (25-30 g of total body weight; n=3 mice per time point per compound). Blood samples were collected in heparinized tubes at up to 7 time points after this injection (i.e. 0.08, 0.25, 0.5, 1, 2, 4 and 8 hr), and the plasma concentrations of the test peptide were determined at each time point by LC-MS/MS, using a five-point standard curve covering a peptide concentration range from 3 to 3,000 ng/mL. Standard curves were prepared using blank plasma matrices and treated in the same manner as the test samples. The main pharmacokinetic parameters determined in these analyses were: terminal half-life ($t_{1/2}$), plasma clearance (CL), volume of distribution (V_d), and area under the plasma concentration versus time curve (AUC) (Figure 7A, Table S6). Values were calculated from the data of the peptide plasma concentration versus time curves using a non-compartmental model analysis, as described elsewhere (Groulx, 2006; Landaw and Di Stefano 3rd, 1982). The doses of z-DTP2 and DTP3 predicted to achieve the therapeutic plasma concentration of 1 µM at the steady state were calculated from the measured pharmacokinetic values using the following equation: $k_0 = k_1 \times V_d \times C_p$ (where V_d is expressed in L/kg; C_p is the plasma concentration at the steady state expressed in mg/L; k_0 is the zero order input expressed in mg/kg/hr; k_1 is the first order elimination constant [$0.693/t_{1/2}$]). The calculated k_0 values of DTP3 and z-DTP2 on the basis of this equation were 0.81 mg/kg/hr and 3.61 mg/kg/hr, respectively. The measured plasma concentration of DTP3 after continual administration via osmotic pumps was very similar to the predicted plasma concentration of 1 µM.

Mouse Xenografts

Mice were housed in the animal facilities at the Hammersmith Campus, Imperial College London, and experiments were conducted under the Home Office Authority (PPL 70/6874). The PPL was reviewed by the Imperial College Ethical Review Process (ERP) and the Home Office. For experiments in the subcutaneous xenograft models (Figures 7B-E, Figures S7A-B, Figures S7C-D), 6 to 8-week old male NOD/SCID mice (NOD.CB17-Prkdc^{scid}/IcrCrl; Charles River) were injected intraperitoneally with anti-CD122 monoclonal antibody (TM-β1 clone; 200 µg/mouse) (Tanaka et al., 1991) and, 24 hr later, subcutaneously in the left flank with 1.0×10^7 U266 or KMS-11 human MM cells resuspended in 200 µL of sterile PBS and Matrigel (1:1) (BD Biosciences), using a 1-mL Luer-Lok™ Tip syringe (BD Biosciences) with a 25-gauge needle (Bhutani et al., 2007). U266 and KMS-11 cells were harvested

from exponentially growing cultures, washed once with serum-free medium, and resuspended in PBS immediately before injection (Bhutani et al., 2007). The anti-CD122 antibody was affinity purified using HiTrap Protein G HP columns (GE Healthcare), according to the manufacturer's instructions. One week after injection of the cells, when tumor volumes had reached approximately 100 mm^3 (U266 cells) or tumors had yet to be palpable (KMS-11 cells), mice were randomized into treatment groups (n=16 or n=4 per group, respectively; day 0) (Bhutani et al., 2007) and, then, treated by continual infusion of DTP3 at a dose of 14.5 mg/kg/day, yielding a plasma concentration of 0.70 ± 0.09 μM (SEM; n=16), or PBS. Treatments were administered using Alzet osmotic pumps (model 1004; 28-day delivery at the rate of 0.11 μL/hr; Charles River), implanted surgically subcutaneously into the right flank of each mouse, according to the manufacturer's instructions (Gullapalli et al., 2012). For systemic administration, DTP3 was dissolved at the concentration of 147 mg/mL in PBS, and mice were euthanized on day 28 of the start of treatment with CO_2. Tumor diameters were measured at the indicated times using a vernier caliper, and volumes were estimated from caliper measurements using the following equation: volume = $AxB^2/2$ (where A is the larger diameter, and B is the smaller diameter of the tumor) (Mauro et al., 2011). Results were reproduced at least twice. The plasma concentration of DTP3 was measured at the steady state by LC-MS/MS at Cyprotex, as described above.

For the in vivo analyses of apoptosis and JNK activation (Figures 7D-E, Figure S7B), mice were randomized into treatment groups when tumor volumes were approximately 200 mm^3 and, then, treated with DTP3 or PBS as above. At the times indicated, mice were sacrificed with CO_2, and tumors were isolated for analysis. For PI nuclear staining analyses, tumors were minced with a scalpel and incubated for 40 min at 37^oC with collagenase D (5 mg/mL; Roche) in 5 mL of complete RPMI-1640 medium, as described previously (Robinson et al., 2003). Single cell suspensions were prepared by passing the digested tumor tissue through a 70-μm cell strainer. Cells were then washed with PBS, stained with PI ($5.0x10^5$ cells/sample), and analyzed by flow cytometry with a FACSCalibur automated system (Becton Dickinson) and FlowJo software, as described above for cell lines (Riccardi and Nicoletti, 2006). TUNEL assays were performed on paraffin-embedded sections of the tumor tissue as reported previously (Papa et al., 2008), using the In Situ Cell Death Detection kit (Roche), according to the manufacturer's instructions, and images were acquired using a Leica SP5 confocal microscope (Leica). Western blots were performed using tumor homogenates prepared in lysis buffer as described previously (Papa et al., 2008).

For experiments in the medullary xenograft model (Figure 7F), 6 to 8-week old male NOD/SCID mice (NOD.CB17-Prkdc^{scid}/IcrCrl; Charles River) were sub-lethally irradiated (3.50 Gy) and, then, injected intraperitoneally with purified anti-CD122 monoclonal antibody (200 µg/mouse), as above. 24 hr later, mice were injected intravenously with KMS-12-BM human MM cells (1.0x10^7 cells/mouse) resuspended in 100 µL of PBS, as described previously (Rabin et al., 2007). As for subcutaneous xenografts, KMS-12-BM cells were harvested from exponentially growing cultures, washed once with serum-free medium, and resuspended in PBS immediately before the injection into mice. Three days after injection of the cells, mice were randomized into two treatment groups (n=8 per group; day 0), as shown in Figure 7F. Treatments were maintained for a period of 8 weeks, and the experiment was stopped on day 161 of treatment start. DTP3 was administered intermittently by infusion using Alzet osmotic pumps (model 1002; 14-day delivery at the rate of 0.25 µL/hr; Charles River) implanted subcutaneously, as described above, two-week on/two-week off, at the dose of 29.0 mg/kg/day. PBS in the control group was also administered for 8 weeks. Animals were monitored daily for weight loss and other clinical signs of medullary disease, such as hind limb paralysis and behavioural changes, and euthanized with CO_2 on day 161 of treatment start or when the disease severity reached any of the end-points stated in the PPL. Results were reproduced at least twice.

Patients

Patients were recruited at several Italian Institutions under the umbrella of the “Gruppo Italiano Malattie Ematologiche dell’Adulto” (GIMEMA) and at the Haematology Clinic at Hammersmith Hospital (Imperial College Healthcare NHS Trust), in accordance with ethics committee approval reference numbers VMP-VMPT trial 163 and 11/LO/1628, respectively. The studies were approved by the Ethics Committee of the “Ospedale San Giovanni Battista” (Turin, Italy) or the London Harrow Research Ethics Committee. Informed written consent was obtained from all the subjects involved in the studies. For the analyses shown in Figure 1A, the relative *GADD45B* mRNA expression was determined by qRT-PCR in purified monoclonal CD138$^+$ cells from 101 MM patients, monoclonal CD138$^+$ cells from 14 patients with monoclonal gammopathy of undetermined significance (MGUS), and polyclonal CD138$^+$ cells from 10 patients with unrelated diseases and without PC dyscrasia. The monoclonality or polyclonality of the isolated PC populations was verified by flow cytometry, as described previously (Rawstro et al., 2008). The analyses shown in Figures 1B-C were conducted on the 69 of the 101 MM

patients in Figure 1A who were enrolled in the VMP arm of the VMP-VMPT trial (clinicaltrial.gov NCT01063179) (Palumbo et al., 2010). The inclusion and exclusion criteria for this trial are described elsewhere (Palumbo et al., 2010). All the patients included in the analysis shown in Figures 1B-C were transplant-ineligible and uniformly treated with nine cycles of velcade-melphalan-prednisone (VMP). The median follow up was of 49 months. PCs were purified at diagnosis from bone marrow aspirates of these patients and stored in the tissue bank of the Hematology Department at the University of Turin. The median age of the patients at diagnosis was 71 years (range 65-86 years), with a female/male ratio of 33/36. The median albumin level was 3.85 g/dL (range 2.2-5.0 g/dL); the median β2-microglobulin level was 4.7 mg/L (range 1.9-12.1 mg/L); and the disease stage according to the International Staging System (Greipp et al., 2005) was as follows: 15 patients (21%), stage 1; 26 patients (37%), stage 2; 15 patients (22%), stage 3; 13 patients (19%), undetermined stage. Based on their FISH profile, 45 patients (66%) were standard risk, and 23 patients (34%) were high risk.

The difference in *GADD45B* mRNA expression between PCs from MGUS and MM patients was calculated using unpaired, two-tailed t-test (Figure 1A). The treatment response of the 69 patients shown in Figures 1B-C was assessed using the standard International Myeloma Working Group (IMWG) Uniform Response Criteria (Durie et al., 2006). Patients were stratified into two groups on the basis of the *GADD45B* mRNA levels in $CD138^+$ cells at diagnosis, as determined by qRT-PCR, using as cut-off the median *GADD45B* expression value as follows: low *GADD45B*, bottom 50th percentile; high *GADD45B*, top 50th percentile. The progression free survival (PFS) and overall survival (OS) of these patients (Figures 1B-C) were defined according to the standard IMWG Uniform Response Criteria (Durie et al., 2006) and calculated using the Kaplan-Meier method. The comparisons between the two groups were analyzed using the log-rank test. At the measured endpoint (i.e. 70.7 months), the PFS was 52% for the patients with low *GADD45B* mRNA expression as compared with the PFS of 17% for the patients with high *GADD45B* mRNA expression. The OS at the same endpoint was 72% for the patients with low *GADD45B* mRNA expression, and it was 42% for those with high *GADD45B* mRNA expression. Results were considered significant with a p value <0.05.

All the other subjects, including seventeen additional MM patients, three patients with Waldenström's macroglobulinemia, and one healthy volunteer, were recruited at the Haematology Clinic at the Hammersmith Hospital, adopting the same diagnostic criteria used above (Durie et al., 2006).

For external validation of the data shown in Figures 1B-C, we examined two independent gene expression datasets of newly diagnosed patients with MM (Figures S1A-B). The first dataset was obtained from the MRC Myeloma IX study performed on 274 patients using the Affymetrix Gene-Chip Human Genome U133 Plus 2.0 Array platform and deposited in the National Center for Biotechnology Information (NCBI) Gene Expression Omnibus (GEO) database, under the accession number, GSE15695 (Dickens et al., 2010; Wu et al., 2011). The trial consisted of two treatment pathways: an intensive treatment pathway, comprising high dose melphalan (HDM) and autologous stem cell transplantation after induction with CTD (cyclophosphamide, thalidomide, dexamethasone) or CVAD (cyclophosphamide, vincristine, doxorubicin, dexamethasone), for younger and fitter patients, and a non-intensive treatment pathway, comprising either attenuated CTD (CTDa) or MP (melphalan, prednisolone), for older and less fit patients (Dickens et al., 2010; Wu et al., 2011). The second dataset was obtained from the Dutch-Belgian/German HOVON-65/GMMG-HD4 study performed on 320 MM patients using the same Affymetrix platform and deposited in the GEO database under the accession number, GSE19784 (Broyl et al., 2010). This trial consisted of two treatment arms: a bortezomib based treatment arm (bortezomib, adriamycine, dexamethasone) (PAD), and a vincristine based treatment arm (vincristine, adriamycine, dexamethasone) (VAD), as induction therapy, followed by HDM treatment with autologous stem cell rescue and maintenance treatment with thalidomide (VAD arm) or bortezomib (PAD arm) (Broyl et al., 2010, Sonneveld et al., 2008).

Gene expression values in the two datasets were both normalized using robust multi-array average (RMA) (Irizarry et al., 2003). In order to obtain homogeneous datasets, patients were separated according to therapeutic arm. Analyses were performed on the 155 patients present in the intensive arm of the MRC Myeloma IX study (GSE15695), and the 149 patients present in the VAD arm of the HOVON-65/GMMG-HD4 study (GSE19784). Sample purity was as reported previously (Broyl et al., 2010, Dickens et al., 2010). For each study, patients were stratified using k-mean clustering based on the *GADD45B* expression values (Hartigan and Wong, 1979). Survival analysis was performed in R using package survival (http://www.R-project.org/). Differences between curves were tested for statistical significance using the log-rank test (Harrington and Fleming, 1982), with p value < 0.05 taken as the level of significance.

Statistical Analyses

Results were expressed as the mean of the indicated number of samples. The statistical error was calculated as either standard deviation (SD) or standard error of the mean (SEM), as indicated, according to the typology of the experiments. Unless otherwise stated, the statistical significance of the values was determined using the Student's t-test. A value of $p<0.05$ was considered statistically significant. In Figure 1A, the difference in *GADD45B* mRNA expression between PCs from MGUS and MM patients was calculated using unpaired, two-tailed t-test. The PFS and OS of MM patients in Figures 1B-C and Figures S1A-B were calculated using the Kaplan-Meier method, and comparisons between the cohorts of patients were analyzed using the log-rank test. The correlations between *GADD45B* mRNA expression and D-peptide cellular activity were analyzed by calculating the Spearman correlation coefficient (r_S) and the corresponding p value (two-tailed) (Figure 5C, Figure S5U). The median OS for the cohorts of tumor-bearing mice shown in Figure 7F was calculated using GraphPad software, and comparisons between the cohorts were analyzed using the log-rank test. The sample size for the analyses shown was not pre-determined. No samples or animals were excluded from the analyses. No specific method of randomization was used for the animal studies. The animal experiments were not blinded.

SUPPLEMENTAL REFERENCES

Ahmad, F., Mani, J., Kumar, P., Haridas, S., Upadhyay, P., and Bhaskar, S. (2011). Activation of anti-tumor immune response and reduction of regulatory T cells with *Mycobacterium indicus pranii* (MIP) therapy in tumor bearing mice. PLoS One *6*, e25424.

Arnold, A.A., Aboukameel, A., Chen, J., Yang, D., Wang, S., Al-Katib, A., and Mohammad, R.M. (2008). Preclinical studies of Apogossypolone: a new nonpeptidic pan small-molecule inhibitor of Bcl-2, Bcl-x_L and Mcl-1 proteins in Follicular Small Cleaved Cell Lymphoma model. Mol. Cancer. *14*, 7-20.

Baroni, M., Cruciani, G., Sciabola, S., Perruccio, F., and Mason, J.S. (2007). A common reference framework for analyzing/comparing proteins and ligands. Fingerprints for Ligands and Proteins (FLAP): theory and application. J. Chem. Inf. Model. *47*, 279-294.

Benkert, P., Biasini, M., and Schwede, T. (2011). Toward the estimation of the absolute quality of individual protein structure models. Bioinformatics *27*, 343-350.

Bhutani, M., Pathak, A.K., Nair, A.S., Kunnumakkara, A.B., Guha, S., Sethi, G., and Aggarwal, B.B. (2007). Capsaicin Is a Novel Blocker of Constitutive and Interleukin-6 -Inducible STAT3 Activation. Clin. Cancer Res. *13*, 3024-3032.

Blanc, E., Roversi, P., Vonrhein, C., Flensburg, C., Lea, S.M., and Bricogne, G. (2004). Refinement of severely incomplete structures with maximum likelihood in BUSTER-TNT. Acta Crystallogr. Section D: Biol. Crystallogr. *60*, 2210-2221.

Carosati, E., Sciabola, S., and Cruciani, G. (2004). Hydrogen bonding interactions of covalently bonded fluorine atoms: from crystallographic data to a new angular function in the GRID force field. J. Med. Chem. *47*, 5114-5125.

Cohen, S.X., Ben Jelloul, M., Long, F., Vagin, A., Knipscheer, P., Lebbink, J., Sixma, T.K., Lamzin, V.S., Murshudov, G.N., Perrakis, A. (2008). ARP/wARP and molecular replacement: the next generation. Acta Crystallogr. Section D: Biol. Crystallogr. *64*, 49-60.

Cowtan, K. (2010). Recent developments in classical density modification. Acta Crystallogr. Section D: Biol. Crystallogr. *66*, 470-478.

Durie, B.G., Harousseau, J.L., Miguel, J.S., Bladé, J., Barlogie, B., Anderson, K., Gertz, M., Dimopoulos, M., Westin, J., Sonneveld, P., et al. (2006). International uniform response criteria for multiple myeloma. Leukemia *20*, 1467-1473.

Eichler, J., and Houghten, R.A. (1995). Generation and utilization of synthetic combinatorial libraries. Mol. Med. Today *1*, 174.

Eswar, N., Webb, B., Marti-Renom, M.A., Madhusudhan, M.S., Eramian, D., Shen, M.Y., Pieper, U., and Sali, A. (2007). Comparative protein structure modeling using MODELLER. Curr. Protoc. Protein Sci. Chapter 2, Unit 2.9.

Greipp, P.R., San Miguel, J., Durie, B.G., Crowley, J.J., Barlogie, B., Bladé, J., Boccadoro, M., Child, J.A., Avet-Loiseau, H., Kyle, R.A., et al. (2005). International staging system for multiple myeloma. J. Clin. Oncol. *23*, 3412-3420.

Griffiths, M., and Sundaram, H. (2011). Drug design and testing: profiling of antiproliferative agents for cancer therapy using a cell-based methyl-[^{3}H]-thymidine incorporation assay. Methods Mol. Biol. *731*, 451-465.

Groulx, A. (2006). Introduction to Pharmacokinetics. Scian. News. *9*, 1-5.

Gullapalli, R., Wong, A., Brigham, E., Kwong, G., Wadsworth, A., Willits, C., Quinn, K., Goldbach, E., and Samant, B. (2012). Development of ALZET® osmotic pump compatible solvent compositions to solubilize poorly soluble compounds for preclinical studies. Drug Deliv. *19*, 239-246.

Harrington, D.P., and Fleming, T. R. (1982). A class of rank test procedures for censored survival data. Biometrika *69*, 553-566.

Irizarry, R.A., Hobbs, B., Collin, F., Beazer-Barclay, Y.D., Antonellis, K.J., Scherf, U., and Speed, T.P. (2003). Exploration, normalization, and summaries of high density oligonucleotide array probe level data. Biostatistics *4*, 249-264.

Kozakov, D., Hall, D.R., Beglov, D., Brenke, R., Comeau, S.R., Shen, Y., Li, K., Zheng, J., Vakili, P., Paschalidis, ICh., and Vajda, S. (2010). Achieving reliability and high accuracy in automated protein docking: ClusPro, PIPER, SDU, and stability analysis in CAPRI rounds 13-19. Proteins *78*, 3124-3130.

Kozakov, D., Hall, D.R., Chuang, G.Y., Cencic, R., Brenke, R., Grove, L.E., Beglov, D., Pelletier, J., Whitty, A., and Vajda, S. (2011). Structural conservation of druggable hot spots in protein-protein interfaces. Proc. Natl. Acad. Sci. U.S.A. *108*, 13528-13533.

Kozlowski, L.P, and Bujnicki, J.M. (2012). MetaDisorder: a meta-server for the prediction of intrinsic disorder in proteins. BMC Bioinformatics *24*, 13.

Landaw, E.M., and Di Stefano 3rd, J.J. (1982). Multiexponential, multicompartmental, and noncompartmental modeling. II. Data analysis and statistical considerations. Am. J. Physiol. Regul. Integr. Comp. Physiol. *243*, 1-6.

Li, Y., Roy, A., and Zhang, Y. (2009). HAAD: A quick algorithm for accurate prediction of hydrogen atoms in protein structures. PLoS One *4*, e6701.

Ngan, C.H., Hall, D.R., Zerbe, B., Grove, L.E., Kozakov, D., and Vajda, S. (2012). FTSite: high accuracy detection of ligand binding sites on unbound protein structures. Bioinformatics. *28*, 286-287.

Rawstron, A.C., Orfao, A., Beksac, M., Bezdickova, L., Brooimans, R.A., Bumbea, H., Dalva, K., Fuhler, G., Gratama, J., Hose, D., et al. (2008). Report of the European Myeloma Network on multiparametric flow cytometry in multiple myeloma and related disorders. Haematologica *93*, 431-438.

Riccardi, C., and Nicoletti, I. (2006). Analysis of apoptosis by propidium iodide staining and flow cytometry. Nat. Protoc. *1*, 1458-1461.

Robinson, S.C., Scott, K.A., Wilson, J.L., Thompson, R.G., Proudfoot, A.E., and Balkwill, F.R. (2003). A chemokine receptor antagonist inhibits experimental breast tumor growth. Cancer Res. *63*, 8360-8365.

Sandomenico, A., Severino, V., Chambery, A., Focà, A., Focà, G., Farina, C., and Ruvo, M. (2013). A comparative structural and bioanalytical study of IVIG clinical lots. Mol. Biotechnol. 54, 983-995.

Schuster, S.R., Rajkumar, S.V., Dispenzieri, A., Morice, W., Aspitia, A.M., Ansell, S., Kyle, R., and Mikhael, J. (2010). IgM multiple myeloma: disease definition, prognosis, and differentiation from Waldenström's macroglobulinemia. Am. J. Hematol. *85*, 853-855.

Söding, J., Biegert, A., and Lupas, A.N. (2005). The HHpred interactive server for protein homology detection and structure prediction. Nucleic Acids Res. *1*, 33 (Web Server issue): W244-8.

Sonneveld, P., van der Holt, B., Schmidt-Wolf, I. G.H., Bertsch, U., el Jarari, L. Salwender, H.J., Zweegman, S., Vellenga, E., Schubert, J., Blau, I.W., et al. (2008). First analysis of HOVON-65/GMMG-HD4 randomized Phase III trial comparing bortezomib, adriamycine, dexamethasone (PAD) vs VAD as induction treatment prior to high dose melphalan (HDM) in patients with newly diagnosed multiple myeloma (MM). Blood *112*, Abstract 653 [ASH Annual Meeting Abstracts].

Tanaka, T., Tsudo, M., Karasuyama, H., Kitamura, F., Kono, T., Hatakeyama, M., Taniguchi, T., and Miyasaka, M. (1991). A novel monoclonal antibody against murine IL-2 receptor beta-chain. Characterization of receptor expression in normal lymphoid cells and EL-4 cells. J. Immunol. *147*, 2222-2228.

Van Dijk, M., and Bonvin, A.M. (2010). The HADDOCK web server for data-driven biomolecular docking. Nat. Protoc. *5*, 883-897.

Vianello, F., Villanova, F., Tisato, V., Lymperi, S., Ho, KK., Gomes, A.R., Marin, D., Bonnet, D., Apperley, J., Lam, E.W., and Dazzi, F. (2010). Bone marrow mesenchymal stromal cells non-selectively protect chronic myeloid leukemia cells from imatinib-induced apoptosis via the CXCR4/CXCL12 axis. Haematologica *95*, 1081-1089.

Wallner, B., and Elofsson, A. (2003). Can correct protein models be identified? Protein Sci. *12*, 1073-1086.

Wu, P., Walker, B.A., Brewer, D., Gregory, W.M., Ashcroft, J., Ross, F.M., Jackson, G.H., Child, A.J., Davies, F.E., Morgan, G.J. (2011). A gene expression-based predictor for myeloma patients at high risk of developing bone disease on bisphosphonate treatment. Clin Cancer Res *17*, 6347-6355.

Yang, H., Bocchetta, M., Kroczynska, B., Elmishad, A.G., Chen, Y., Liu, Z., Bubici, C., Mossman, B.T., Pass, H.I., Testa, J.R., Franzoso, G., and Carbone, M. (2006). TNF-inhibits asbestos-induced cytotoxicity via a NF-κB-dependent pathway, a possible mechanism for asbestos-induced oncogenesis. Proc. Natl. Acad. Sci. U S A *103*, 10397-10402.

Yang, J., Zhang, X., Wang, J., Qian, J., Zhang, L., Wang, M., Kwak, L.W., and Yi, Q. (2007). Anti-β2-microglobulin monoclonal antibodies induce apoptosis in myeloma cells by recruiting MHC class I to and excluding growth and survival cytokine receptors from lipid rafts. Blood *110*, 3028-3035.

Zhang, J., Liang, Y., and Zhang, Y. (2011). Atomic-level protein structure refinement using fragment-guided molecular dynamics conformation sampling. Structure *19*, 1784-1795.
